# Unraveling the structure–property relationships in fluorescent phenylhydrazones: the role of substituents and molecular interactions†

**DOI:** 10.1039/d4ra07856j

**Published:** 2025-01-17

**Authors:** Paulina Sobczak, Tomasz Sierański, Marcin Świątkowski, Agata Trzęsowska-Kruszyńska, Jolanta Kolińska

**Affiliations:** a Institute of General and Ecological Chemistry, Lodz University of Technology Zeromskiego 116 Lodz 90924 Poland paulina.sobczak@dokt.p.lodz.pl; b Łukasiewicz – Lodz Institute of Technology M. Sklodowskiej-Curie 19/27 90570 Lodz Poland; c Institute of Polymer and Dye Technology, Lodz University of Technology Stefanowskiego 16 Lodz 90537 Poland

## Abstract

This study investigates the structure–property relationships of a series of phenylhydrazones bearing various electron-donating and electron-withdrawing substituents, such as methoxy, dimethylamino, morpholinyl, hydroxyl, chloro, bromo, and nitro groups. The compounds were synthesized, and their structures were characterized using single-crystal X-ray diffraction, powder X-ray diffraction, FTIR spectroscopy, NMR spectroscopy, and DSC. Three-dimensional excitation–emission matrix (3D-EEM) fluorescence spectroscopy and UV-Vis spectroscopy were employed to elucidate the complex interplay between the molecular skeleton, substituents, and the resulting photophysical properties. Quantum mechanical calculations provided further insights into the electronic structure and excited-state dynamics of the investigated compounds. The phenylhydrazones exhibited emission wavelengths ranging from 438 to 482 nm, with the molecular backbone playing a crucial role in determining the emission wavelength. The incorporation of electron-donating substituents, such as methoxy and dimethylamino groups, led to enhanced fluorescence intensity, while the presence of nitro groups, resulted in complete fluorescence quenching. This comprehensive study establishes rational design principles for the development of highly emissive phenylhydrazones with tuned photophysical properties and highlights the significance of the molecular skeleton in dictating the fluorescence behavior of this class of compounds.

## Abstract 2

The fluorescence of phenylhydrazones: understanding the critical role of substituents in influencing the intensity of fluorescence and the evolution of emissive materials.

## Graphical abstract

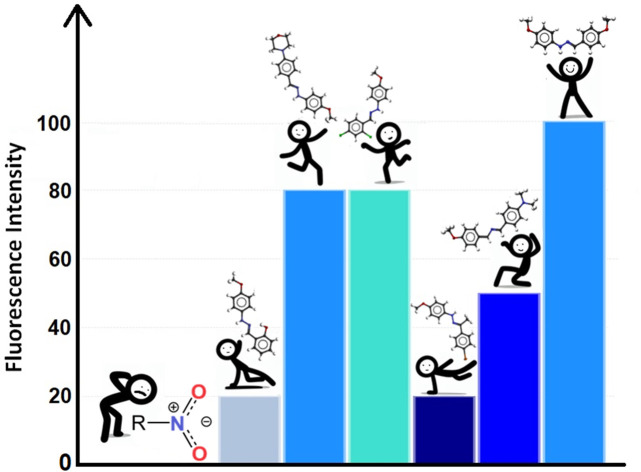

## 1 Introduction

Fluorescence, a phenomenon involving the emission of light from electronically excited states following the absorption of electromagnetic radiation, has attracted significant attention across various scientific disciplines, including physics,^1^ chemistry,^2–4^ materials science,^5,6^ biology,^7–11^ and medicine.^12–17^ Of particular interest are organic compounds that exhibit fluorescent properties in the solid state, owing to their potential applications in optoelectronics,^18,19^ forensics,^20,21^ and biomedicine.^22,23^

Hydrazones represent a particularly promising class of such compounds, exhibiting a remarkable range of applications. Beyond their established therapeutic roles in pharmaceutical sciences,^24–28^ these compounds have gained significant recognition for their fluorescent properties. Their unique photophysical characteristics and structural versatility make them especially valuable in various applications where fluorescence-based detection and sensing are crucial.^29^ The exceptional molecular design of hydrazones enables their diverse functionality in fluorescence-based applications, from biological sensing to materials science. These compounds serve as efficient fluorescent probes for detecting various metal ions in biological systems, with notable success in tracking magnesium, copper, and mercury ions within cellular environments. Additionally, they have proven effective in monitoring mitochondrial viscosity fluctuations, providing crucial insights into diverse disease mechanisms.^30–33^ Their molecular architecture enables selective interactions with specific functional groups, making them valuable tools in both analytical chemistry and organic synthesis, especially as selective fluorescent sensors for Fe(iii) ions, acid vapors, and environmental pollutants such as *p*-nitrophenol.^34–36^ In materials science and optoelectronics, fluorescent hydrazone derivatives demonstrate remarkable versatility, serving as active components in light-emitting diodes for display technologies, efficient materials in dye-sensitized solar cells for renewable energy applications, and key elements in advanced polymer-based electronic devices.^37,38^ Their photoswitch properties enable controlled fluorescence modulation through light stimulation, leading to the development of sophisticated “off–on” or dual-state fluorescent switches essential for next-generation optical data storage systems.^39^ In the field of forensics and security applications, these compounds have revolutionized document authentication and anti-counterfeiting measures through their unique solid-state fluorescent properties. Their ability to produce tunable emissions in the solid state has led to the development of advanced security inks and sophisticated encoding systems for polychromic images, providing robust solutions for document security that are simultaneously difficult to counterfeit yet simple to verify.^40,41^ The growing interest in these compounds extends to environmental monitoring, where paper-based sensors coupled with smartphone technology enable rapid and visual detection of pollutants.^42^ Their exceptional molecular recognition capabilities and ability to form specific metal complexes further enhance their potential in biomedicine, particularly in areas requiring highly selective and sensitive detection methods for disease diagnosis and monitoring.^43^

Given the broad spectrum of applications and the importance of hydrazones in various fields, there is a growing need to develop compounds with precisely controlled properties, including their fluorescent characteristics. This requires a thorough understanding of structure–property relationships and the ability to fine-tune the photophysical characteristics through molecular design. In this context, the rational modification of molecular structure emerges as a key strategy for optimizing the fluorescent properties of these compounds.

The rational design and modification of organic compounds to achieve tunable and switchable fluorescent materials have become a central focus in the development of solid-state emissive organic chromophores. One promising strategy involves the minimization of π⋯π stacking interactions through the introduction of judiciously selected functional groups into the molecular structure.^44–47^ The nature and position of these substituents, classified as electron-donating (EDS) or electron-withdrawing (EWS), play a crucial role in modulating the fluorescent properties by altering the electron density distribution, extending the conjugation in the aromatic system, and influencing the molecular geometry.^48–50^

The incorporation of substituents into the molecular framework can also affect the preferred conformation of the fluorophore, which in turn modifies the energy gap between the highest occupied molecular orbital (HOMO) and the lowest unoccupied molecular orbital (LUMO), ultimately impacting the fluorescent behavior.^51,52^ Moreover, the ability of substituents to form intramolecular hydrogen bonds (*e.g.*, O–H⋯N) and engage in non-covalent weak interactions (*e.g.*, C–H⋯O or C–H⋯N) can significantly influence the conformation and, consequently, the photophysical properties of the fluorophore.^53^

Phenylhydrazones, a class of organic compounds characterized by an azomethine bond (C=N), have emerged as promising candidates for solid-state fluorescence applications. Extensive research efforts have been dedicated to explaining the effects of EDS and EWS on the fluorescence properties of phenylhydrazones.^54,55^ While the introduction of EDS has been reported to enhance fluorescence intensity and quantum yield, the incorporation of EWS has been shown to quench emission. These contrasting effects are attributed to the stabilization or destabilization of the HOMO–LUMO energy gap by EDS and EWS, respectively.^52,56,57^ Furthermore, EDS tend to increase the planarity and rigidity of hydrazone molecules, whereas EWS often promote twisted conformations and enhanced molecular packing. The interplay between these structural factors and the specific nature and position of the substituents adds complexity to the fluorescence behavior of phenylhydrazones.^52,58^

Among the various functional groups, the nitro group (–NO_2_) stands out as a particularly intriguing substituent in the context of fluorescence.^59^ As a strong electron-withdrawing group, –NO_2_ significantly reduces the electron density within aromatic rings and enhances their polarity, leading to an increased HOMO–LUMO energy gap and a consequent reduction in fluorescence intensity and quantum yield in the solid state. While in most cases, the nitro group acts as a fluorescence quencher, it's worth noting that in specific molecular structures and environments, its effect can be more complex, occasionally even leading to fluorescence enhancement.^59–61^

In addition to the nature and position of substituents, the molecular skeleton of the fluorophore plays a vital role in determining its photophysical properties. The extent of conjugation, rigidity, and symmetry of the molecular backbone can significantly influence the electronic structure, excited-state dynamics, and intermolecular interactions. For instance, the incorporation of fused aromatic rings or the extension of the conjugated system can lead to bathochromic shifts in absorption and emission, while the introduction of flexible or bulky moieties can disrupt the planarity and affect the fluorescence quantum yield.^62–66^

In light of these considerations, it is hypothesized that the careful selection of the molecular skeleton, in combination with the strategic incorporation of EDS and EWS, will enable the fine-tuning of the fluorescent properties of phenylhydrazones. By systematically investigating the structure–property relationships, the aim is to establish rational design principles for the development of highly emissive functional materials with tuned emission characteristics.

The objectives of this research are diverse. Firstly, it seeks to synthesize a series of phenylhydrazones with close-up molecular skeletons and different substituent patterns, encompassing a range of EDS and EWS. Secondly, it aims to clarify the complex relationships between the molecular structure, crystal packing, and the resulting fluorescence behavior, employing a multidisciplinary approach that includes X-ray diffraction, spectroscopic techniques, and quantum mechanical calculations. Thirdly, efforts were made to discover the mechanisms underlying fluorescence quenching or enhancement in phenylhydrazones, with a particular emphasis on the role of nitro groups and other strong electron-withdrawing substituents. Finally, it seeks to establish structure–property correlations that will guide the rational design of advanced functional materials with tuned photophysical properties.

By achieving these objectives, this research is expected to contribute significantly to the fundamental understanding of the fluorescence behavior of phenylhydrazones and provide valuable insights for the development of novel emissive materials with potential applications in optoelectronics, sensing, bioimaging, and beyond. The knowledge gained from this study will not only develop the field of solid-state fluorescence but also pave the way for the design of next-generation organic functional materials with enhanced performance and tuned properties.

## 2 Experimental section

### 2.1 Synthesis

**Scheme 1:**
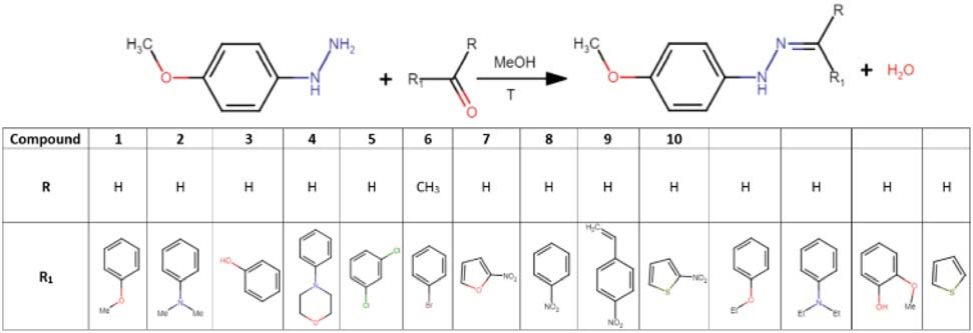
Synthesis of the studied compounds.

Phenylhydrazones were synthesized through a classical condensation reaction, where aldehydes or ketones react with a hydrazine group.^67^ Equimolar amounts (1 mmol) of 4-methoxyphenylhydrazine hydrochloride and the appropriate aldehydes and ketones were used: 4-methoxybenzaldehyde, 4-dimethylaminobenzaldehyde, salicylaldehyde, 4-(4-morpholinyl)benzaldehyde, 2,4-dichlorobenzaldehyde, 4-bromoacetophenone, 5-nitro-2-furaldehyde, 4-nitrobenzaldehyde, *trans*-4-nitrocinnamaldehyde, 5-nitro-2-thiophenecarboxaldehyde, 4-ethoxybenzaldehyde, 4-diethylaminobenzaldehyde, 4-hydroxy-3-methoxybenzaldehyde, and 2-thiophenecarboxaldehyde (Scheme 1). Each substrate mixture was dissolved in methanol (80 cm^3^) and heated at 65 °C for 60 minutes with continuous stirring under ambient atmosphere. After completion of the reaction, the mixture was left to slowly evaporate at room temperature in a partially covered crystallization dish. The crystals appeared within 1–2 weeks and after collection were washed with methanol (3 × 5 mL, 15 °C). The products were stored in a dark, dry place. The purity of the compounds was confirmed by IR, NMR, and powder X-ray diffraction analyses (see Section 3.1). This method proved successful in 10 out of 14 attempted syntheses, yielding the expected crystalline products.

### 2.2 Instrumental studies

#### X-ray diffraction studies

The high-quality crystals of phenylhydrazones (1–10) were chosen and the single crystal X-ray diffraction measurements were carried out on an XtaLAB Synergy Dualflex Pilatus 300 K diffractometer at 100.0(1) K. The structures were solved and refined using SHELXT^68^ and SHELXL,^69^ respectively. All non-hydrogen atoms were refined anisotropically. The N- and O-bound hydrogen atoms were located on the difference Fourier map and refined freely. The remaining hydrogen atoms were positioned geometrically and refined using the riding model. Details regarding crystal data and structural refinement can be found in Table S1.† Supplementary crystallographic information for this study is available under CCDC 2350542–2350551.

The powder X-ray diffraction patterns were recorded in reflection mode using a PANalytical X'Pert Pro MPD diffractometer, equipped with CuKα radiation (*λ* = 1.54059 Å), at room temperature (Fig. S1–S10†).

#### IR spectroscopy

FT-IR spectra were recorded on a JASCO FT/IR-6200 spectrophotometer within the wavenumber range 4000–400 cm^−1^ (Fig. S11–S20†). The samples were prepared as KBr pellets, in which the mass ratio of the analyzed substance to KBr was 1 : 100.

#### NMR

NMR spectra (Fig. S21–S30†) were acquired on a Bruker AVANCE DPX instrument, running at 250 MHz. Chemical shifts (*δ*) are reported in ppm relative to residual proton signals of (CD_3_)_2_S=O.

#### DSC

A differential scanning calorimeter was used to determine the melting or decomposition temperature in an N_2_ atmosphere at a heating/cooling rate of 20 K min^−1^. Samples for DSC measurements were placed in closed aluminum crucibles.

#### Results of FTIR, NMR and DSC measurements

##### (*E*)-1-(4-Methoxybenzylidene)-2-(4-methoxyphenyl)hydrazine (1)

Yield 70%, IR (KBr, cm^−1^) 3308 (*ν*N–H); 2959, 2928, 2900, 2836 (*ν*C_ring_–H); 1607 (*ν*C=N); 1568, 1507 (*ν*C–C_ring_); 1463, 1441 (*ν*C–C_ring_), (*δ*C–H); 1354 (*ν*C–N), (*δ*C–H); 1296 (*ν*_asym_C–O–C), (*ν*C_ring_–O); 1246, 1182 (*δ*C–H); 1135 (*ν*N–N), 1030 (*ν*C(CH_3_)–O); 889, 855 (*ν*_sym_C–O–C); 774, 666 (*δ*C_ring_–H); 596, 530 (*δ*O–C=O). ^1^H NMR (DMSO-d_6_*δ*H) ppm: 9.85 (s, 1H, N–N–H), 7.72 (s, 1H, N=C–H), 7.52–7.49 (m, 2H, Ar–H), 6.95–6.89 (m, 4H, Ar–H), 6.79–6.77 (m, 2H, Ar–H), 3.73 (s, 3H, CH_3_), 3.64 (s, 3H, CH_3_). DSC mp 113 °C.

##### (*E*)-4-((2-(4-Methoxyphenyl)hydrazineylidene)methyl)-*N*,*N*-dimethylaniline (2)

Yield 65%, IR (KBr, cm^−1^) 3298 (*ν*N–H); 2995, 2899, 2837, 2804 (*ν*C_ring_–H); 1611 (*ν*C=N); 1510 (*ν*C–C_ring_); 1440 (*ν*C–C_ring_), (*δ*C–H); 1360 (*ν*C–N), (*δ*C–H); 1240 (*ν*_asym_C–O–C), (*ν*C_ring_–O); 1183 (*δ*C–H); 1131 (*ν*N–N); 1031 (*ν*C(CH_3_)–O); 918, 825 (*ν*_sym_C–O–C); 768, 645 (*δ*C_ring_–H); 604, 528 (*δ*O–C=O). ^1^H NMR (DMSO-d_6_*δ*_H_) ppm: 9.64 (s, 1H, N–N–H), 7.67 (s, 1H, N=C–H), 7.40 (d, *J* = 5.5 Hz, 2H, Ar–H), 6.91 (d, *J* = 5.5 Hz, 2H, Ar–H), 6.77 (d, *J* = 5.5 Hz, 2H, Ar–H), 6.68 (d, *J* = 5.5 Hz, 2H, Ar–H), 3.64 (s, 3H, CH_3_), 2.89 (s, 6H, N(CH_3_)_2_). DSC disintegration 185 °C.

##### (*E*)-2-((2-(4-Methoxyphenyl)hydrazineylidene)methyl)phenol (3)

Yield 63%, IR (KBr, cm^−1^) 3331 (*ν*N–H) (*ν*O–H); 3006, 3047, 2968, 2834 (*ν*C_ring_–H); 1619 (*ν*C=N); 1598, 1587, 1565, 1508 (*ν*C–C_ring_), 1355 (*ν*C–N), (*δ*C–H); 1262 (*δ*O–H); 1238 (*ν*_asym_C–O–C), (*ν*C_ring_–O); 1177 (*δ*C–H); 1156 (*ν*N–N), 1101 (*ν*O–C); 1033 (*ν*C(CH_3_)–O); 863, 814 (*ν*_sym_C–O–C); 743, 661 (*δ*C_ring_–H); 607, 514 (*δ*O–C=O). ^1^H NMR (DMSO-d_6_*δ*_H_) ppm: 10.56 (s, 1H, N–N–H), 8.04 (s, 1H, N=C–H), 7.44 (d, *J* = 5 Hz, 1H, Ar–H), 7.10–7.10 (m, 1H, Ar–H), 6.88–6.82 (m, 6H, Ar–H), 3.65 (s, 3H, CH_3_). DSC mp 133 °C.

##### (*E*)-4-(4-((2-(4-Methoxyphenyl)hydrazineylidene)methyl)phenyl)-morpholine (4)

Yield 61%, IR (KBr, cm^−1^) 3306 (*ν*N–H); 3006, 2960, 2897, 2831 (*ν*C_ring_–H); 1603 (*ν*C=N); 1510 (*ν*C–C_ring_); 1442 (*ν*C–C_ring_), (*δ*C–H); 1382 (*ν*C–N), (*δ*C–H); 1297 (*ν*_asym_C–O–C), (*ν*C_ring_–O); 1262, 1226, 1183, 1180 (*δ*C–H); 1120 (*ν*N–N), 1068 (*δ*C–O–C); 1036 (*ν*C(CH_3_)–O); 866, 828 (*ν*_sym_C–O–C); 760, 659 (*δ*C_ring_–H); 555, 528 (*δ*O–C=O). ^1^H NMR (DMSO-d_6_*δ*_H_) ppm: 9.78 (s, 1H, N–N–H), 7.69 (s, 1H, N=C–H), 7,44 (d, *J* = 5.5 Hz, 2H, Ar–H), 6.93–6.89 (m, 4H, Ar–H), 6.79–6.76 (m, 2H, Ar–H) 3.71–3.69 (m, 4H, O–morpholinyl–H), 3.11–3.09 (m, 4H, N–morpholinyl–H), 3.64 (s, 3H, CH_3_). DSC mp 163 °C.

##### (*E*)-1-(2,4-Dichlorobenzylidene)-2-(4-methoxyphenyl)hydrazine (5)

Yield 68%, IR (KBr, cm^−1^) 3303 (*ν*N–H); 2954, 2930, 2834 (*ν*C_ring_–H); 1587 (*ν*C=N); 1546, 1519 (*ν*C–C_ring_); 1464, 1437 (*ν*C–C_ring_), (*δ*C–H); 1382 (*ν*C–N), (*δ*C–H); 1300 (*ν*_asym_C–O–C), (*ν*C_ring_–O); 1246, 1180 (*δ*C–H); 1145 (*ν*N–N); 1033 (*ν*C(CH_3_)–O); 893, 861 (*ν*_sym_C–O–C); 784, 705, 670 (*δ*C_ring_–H), (*ν*C–Cl); 555, 522 (*δ*O–C=O). ^1^H NMR (DMSO-d_6_*δ*_H_) ppm: 10.57 (s, 1H, N–N–H), 8.03 (s, 1H, N=C–H), 7.96–7.93 (m, 1H, Ar–H), 7.56–7.55 (m, 1H, Ar–H), 7.38–7.36 (m, 1H, Ar–H), 7.00–6.98 (m, 2H, Ar–H), 6.83–6.81 (m, 2H, Ar–H), 3.66 (s, 3H, CH_3_). DSC mp 81 °C.

##### (*E*)-1-(1-(4-Bromophenyl)ethylidene)-2-(4-methoxyphenyl)-hydrazine (6)

Yield 57%, IR (KBr, cm^−1^) 3344 (*ν*N–H); 2995, 2965, 2930, 2840 (*ν*C_ring_–H); 1611 (*ν*C=N); 1592, 1513 (*ν*C–C_ring_); 1481, 1440 (*ν*C–C_ring_), (*δ*C–H); 1396 (*ν*C–N), (*δ*C–H); 1297 (*ν*_asym_C–O–C), (*ν*C_ring_–O); 1246, 1180 (*δ*C–H); 1145 (*ν*N–N), 1033 (*ν*C(CH_3_)–O); 822 (*ν*_sym_C–O–C); 787, 749, 708 (*δ*C_ring_–H); 659 (*ν*C–Br); 555, 520 (*δ*O–C=O). ^1^H NMR (DMSO-d_6_*δ*_H_) ppm: 9.10 (s, 1H, N–N–H), 7.68–7.66 (m, 2H, Ar–H), 7.51–7.49 (m, 2H, Ar–H), 7.14–7.12 (m, 2H, Ar–H), 6.81–6.79 (m, 2H, Ar–H), 3.65 (s, 3H, CH_3_), 2.16 (s, 3H, CH_3_). DSC mp 149 °C.

##### (*E*)-1-(4-Methoxyphenyl)-2-((5-nitrofuran-2-yl)methylene)-hydrazine (7)

Yield 85%, IR (KBr, cm^−1^) 3298 (*ν*N–H); 2960, 2831 (*ν*C_ring_–H); 1557 (*ν*C=N); 1508 (*ν*C–C_ring_); 1494 (*ν*C–C_ring_), (*ν*N–O); 1450, 1417 (*ν*C–C_ring_), (*δ*C–H); 1368 (*ν*C–N), (*δ*C–H); 1322 (*ν*N–O); 1279 (*ν*_asym_C–O–C), (*ν*C_ring_–O); 1243 (*δ*C–H); 1229 (*ν*C–O); 1186 (*δ*C–H); 1158 (*ν*N–N), 1016 (*ν*C(CH_3_)–O); 872, 822 (*ν*_sym_C–O–C); 795, 730 (*δ*C_ring_–H); 561 (*δ*O–C=O). ^1^H NMR (DMSO-d_6_*δ*_H_) ppm: 11.00 (s, 1H, N–N–H), 7.72 (d, *J* = 2.5 Hz 1H, NO_2_–furan–H), 7.68 (s, 1H, N=C–H), 7.04–7.02 (m, 2H, Ar–H), 6.92 (d, *J* = 2.5 Hz 1H, NO_2_–furan–H), 6.87–6.84 (m, 2H, Ar–H), 3.61 (s, 3H, CH_3_). DSC disintegration 190 °C.

##### (*E*)-1-(4-Methoxyphenyl)-2-(4-nitrobenzylidene)hydrazine (8)

Yield 79%, IR (KBr, cm^−1^) 3285 (*ν*N–H); 3006, 2840 (*ν*C_ring_–H); 1598 (*ν*C=N); 1562, 1529 (*ν*C–C_ring_); 1502 (*ν*C–C_ring_), (*ν*N–O); 1445, 1409 (*ν*C–C_ring_), (*δ*C–H); 1335 (*ν*C–N), (*δ*C–H), (*ν*N–O); 1265 (*ν*_asym_C–O–C), (*ν*C_ring_–O); 1232 (*δ*C–H), 1174 (*δ*C–H); 1147 (*ν*N–N), 1022 (*ν*C(CH_3_)–O); 847, 822 (*ν*_sym_C–O–C); 746, 691 (*δ*C_ring_–H); 602, 525 (*δ*O–C=O). ^1^H NMR (DMSO-d_6_*δ*_H_) ppm: 10.71 (s, 1H, N–N–H), 8.17–8.14 (m, 2H, Ar–H), 7.82–7.78 (m, 3H, N=C–H, Ar–H), 7.06–7.03 (m, 2H, Ar–H), 6.86–6.83 (m, 2H, Ar–H), 3.67 (s, 3H, CH_3_). DSC mp 182 °C.

##### (*E*)-1-(4-Methoxyphenyl)-2-(3-(4-nitrophenyl)propylidene)hydrazine (9)

Yield 74%, IR (KBr, cm^−1^) 3306 (*ν*N–H); 2960, 2829 (*ν*C_ring_–H); 1617 (*ν*C=N); 1590, 1557, 1524 (*ν*C–C_ring_); 1502 (*ν*C–C_ring_), (*ν*N–O); 1461 (*ν*C–C_ring_), (*δ*C–H); 1338 (*ν*C–N), (*δ*C–H); 1297 (*ν*_asym_C–O–C), (*ν*C_ring_–O); 1273 (*ν*N–O); 1232 (*δ*C–H), 1183 (*δ*C–H); 1147 (*ν*N–N), 1030 (*ν*C(CH_3_)–O); 872, 831 (*ν*_sym_C–O–C); 746, 689 (*δ*C_ring_–H); 596, 528 (*δ*O–C=O). ^1^H NMR (DMSO-d_6_*δ*_H_) ppm: 10.40 (s, 1H, N–N–H), 8.12 (d, *J* = 5.5 Hz, 2H, Ar–H), 7.73 (d, *J* = 5.5 Hz, 2H, Ar–H), 7.66 (d, *J* = 5.5 Hz, 2H, Ar–H), 7.20–7.18 (m, 1H, C=C–H), 6.95–6.93 (m, 2H, Ar–H), 6.82–6.78 (m, 1H, C=C–H), 3.65 (s, 3H, CH_3_). DSC mp 173 °C.

##### (*E*)-1-(4-Methoxyphenyl)-2-((5-nitrothiophen-2-yl)methylene)-hydrazine (10)

Yield 71%, IR (KBr, cm^−1^) 3276 (*ν*N–H); 2954, 2905, 2831 (*ν*C_ring_–H); 1546 (*ν*C=N); 1505 (*ν*C–C_ring_), (*ν*N–O); 1461, 1434 (*ν*C–C_ring_), (*δ*C–H); 1363 (*ν*C–N), (*δ*C–H); 1300 (*ν*_asym_C–O–C), (*ν*C_ring_–O); 1243 (*ν*N–O); 1224 (*δ*C–H), 1183 (*δ*C–H); 1156 (*ν*N–N), 1036 (*ν*C(CH_3_)–O); 883, 820 (*ν*_sym_C–O–C); 730 (*δ*C_ring_–H); 691 (*ν*C–S) 588, 536 (*δ*O–C=O). ^1^H NMR (DMSO-d_6_*δ*_H_) ppm: 10.95 (s, 1H, N–N–H), 8.00 (d, *J* = 2.75 Hz, 1H, NO_2_–thiophene–H), 7.91 (s, 1H, N=C–H), 7.18 (d, *J* = 2.75 Hz, 1H, NO_2_–thiophene–H), 7.01–6.99 (m, 2H, Ar–H), 6.86–6.84 (m, 2H, Ar–H), 3.70 (s, 3H, CH_3_). DSC mp 150 °C.

#### UV-Vis spectroscopy

The UV-Vis diffuse reflectance spectra (Fig. S31–S50†) were recorded on a Jasco V-660 spectrometer, in the spectral range 200–800 nm, using Spectralon as a standard with 100% reflectance.

#### Fluorescence spectroscopy

The solid-state three-dimensional excitation–emission matrix (3D-EEM) fluorescence spectra were recorded on a JASCO FP-6500 spectrophotometer. All spectra were recorded in the same range of excitation (*λ*_ex_) and emission (*λ*_em_) wavelengths: *λ*_ex_ 220–640 nm, *λ*_em_ 220–745 nm.

#### Fluorescence quantum yields

The absolute fluorescence quantum yields were measured in the solid state using an Edinburgh Instruments FLS920 spectrofluorometer equipped with an integrating sphere, with solid barium sulfate (BaSO_4_) serving as a blank. Each compound was excited at its respective excitation wavelength corresponding to the emission maxima and the values are summarized in Table 1. Prior to the measurements, the compound samples were finely ground to ensure uniformity in physical characteristics, such as grain size and texture.

**Table 1:** Values of maximum fluorescence intensities appearing in fluorescence spectra (*λ*_ex_-excitation wavelength, *λ*_em_-emission wavelength, Int-fluorescence intensity, *ϕ*_f_-fluorescence quantum yield, π–π orbital, n – non-bonding orbital, * – an antibonding orbital)

Compound	*λ*_ex_ (nm)	Emission maxima									Orbital transition
		I		II		III		IV			
		*λ*_em_ (nm)	Int	*λ*_em_ (nm)	Int	*λ*_em_ (nm)	Int	*λ*_em_ (nm)	Int	*ϕ*_f_	
1	415	446	107	467	111					0.12	n(O_methoxy_)/π → π*
	364					446	87	465	89	0.24	n(O_methoxy_)/π → π*
2	428	450	45							0.05	n(O_methoxy_)/π → π*
	359			453	44					0.13	n(O_methoxy_)/π → π*
3	468	482	20							<0.01	n(O_methoxy_)/π → π*
4	424	457	79							0.08	n(O_methoxy_)/π → π*
	366			457	62					0.18	n(O_methoxy_)/π → π*
5	431	476	83							0.07	n(Cl)/n(O_methoxy_)/π → n(Cl)/π*
	359			475	77					0.19	n(Cl)/n(O_methoxy_)/π → n(Cl)/π*
6	434	445	24							<0.010	n(O_methoxy_)/π → π*
	364			438	18					<0.010	n(Br)/n(O_methoxy_)π → n(Br)/π*

### 2.3 Quantum-mechanical calculations

Excited states of the studied systems (1–10) were initially calculated using time-dependent density functional theory (TD-DFT) based on coordinates determined by X-ray diffraction measurements. Initial models were constructed using Mercury software, version 2022.3.0.^70^ To standardize these models, positions of hydrogen atoms were adjusted along their bond vectors to match the average X–H bond length as determined from neutron diffraction data. TD-DFT calculations were performed using Gaussian 09, revision E.01,^71^ utilizing the B3LYP^72^ functional with a 6-31g(2d,2p) basis set.^73,74^ The number of calculated transitions was set to 400. However, for some compounds, particularly 7–10, significant discrepancies were observed between the theoretical absorption spectra and experimental data, especially for bands above 400 nm. To address these discrepancies, additional calculations were performed considering multiple molecules (ranging from 2 to 4) depending on the compound. The selection of molecules was made using Mercury software, taking into account dominant intermolecular interactions empirically calculated by the Mercury software. Specifically, for compounds 1, 3, 4, 5, and 6, calculations were performed using 3 molecules; for compounds 7–10, 4 molecules were used; and for compound 2, 2 molecules were considered. These larger systems were then subjected to TD-DFT calculations using the same basis set and functional as in the single-molecule calculations. For these complex systems, the number of transitions calculated was increased to 800. Correlation between computed excited maxima and experimental fluorescence emission maxima was established by comparing excitation energies and oscillator strengths/intensities of the corresponding maxima. Analysis of the character of orbital excitations was conducted using orbital contour plots generated by Chemissian software, version 4.67.^75^

## 3 Results and discussion

### 3.1 Synthesis results

**Fig. 1:**
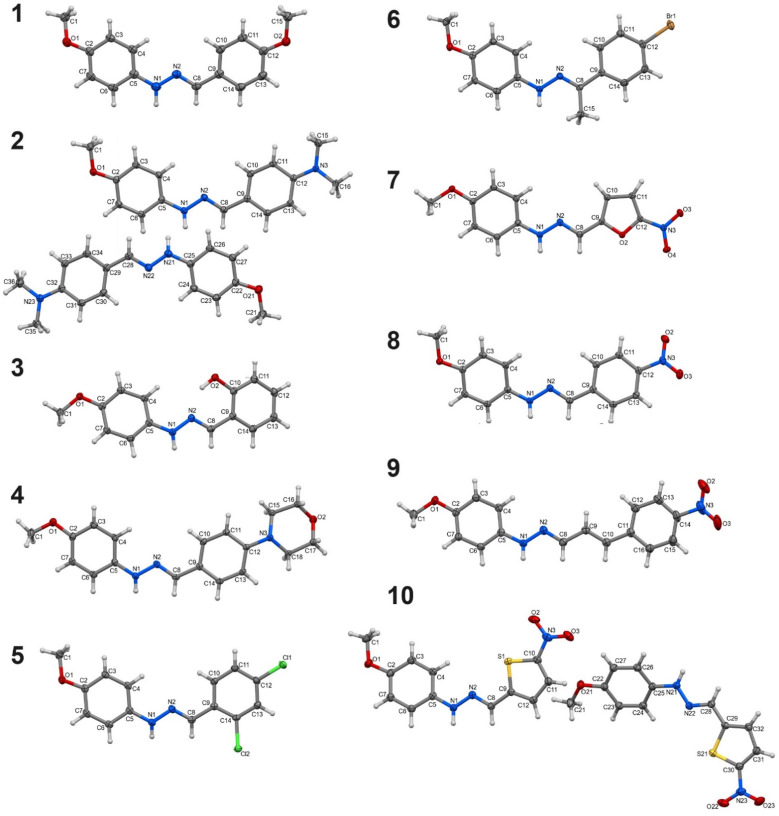
Molecular structures of phenylhydrazones (plotted with 50% probability of displacement ellipsoids).

A total of 14 syntheses were performed in the course of the research. Of these, 10 successfully yielded the desired products in crystalline form, which were structurally characterized and labelled 1–10 (Fig. 1). The remaining 4 reactions (with 4-ethoxybenzaldehyde, 4-diethylaminobenzaldehyde, 4-hydroxy-3-methoxybenzaldehyde, 2-thiophenecarboxaldehyde) resulted in semi-liquid, oily substances, which were not suitable for further characterization in this study. Despite multiple attempts at recrystallization, these semi-liquid products could not be converted into solid forms. The synthesized compounds' phase purity in the bulk sample was validated through PXRD measurements. The experimental PXRD patterns displayed a high degree of consistency with the calculated patterns (generated from CIF files), suggesting the absence of significant crystalline impurities (Fig. S1–S10†).

### 3.2 Crystal and molecular structure analysis

The analysis of bond lengths and angles within the hydrazone moiety –CH=N–NH– reveals that they are typical for this class of compounds^76^ and comparable among the studied molecules 1–10 (Tables S2 and S3†). However, the spatial arrangement of the 5- and 6-membered rings and functional groups around the imine bond varies, leading to different overall conformations. Based on the analysis of the interplane angles between the planes of the rings and the torsion angles within the hydrazone moiety, it can be concluded that compounds 1, 6, 7, and 9 exhibit the most planar geometry (Table S4†). In the remaining compounds, the values of the torsion angle C5–N1–N2–C8 are below 170° (or above −170°) and/or the interplanar angles exceed 10°, indicating a more twisted conformation. The most distorted geometry is observed in one of the two symmetry-independent molecules of compound 2, where the aforementioned angles are −155° and 28.5°, respectively. The orientation of the methoxy group relative to the N1–N2 bond adopts a cis conformation in compounds 1, 2, 5, 6, 8, and 10, while a *trans* conformation is observed in the remaining compounds. In compound 4, the morpholinyl ring adopts a chair conformation.

The studied compounds exhibit a diverse range of intermolecular interactions. In all compounds, except for compound 3, the nitrogen atom from the hydrazone moiety (N1) is the only donor of classical hydrogen bonds. In compounds 1, 5, 6, and 9, this nitrogen atom lacks an acceptor, whereas in the other compounds, it typically forms N–H⋯O hydrogen bonds (Table S5†). In compounds 7 and 10, the nitro group serves as the acceptor. In compound 7, a dimeric R_2_^2^(18) synthon is created, while in compound 10, a bifurcated hydrogen bond forms, where the hydrogen from the N1 nitrogen atom interacts with both oxygen atoms of the nitro group (Fig. S51 and S52†). Despite each molecule having a methoxy group, a hydrogen bond involving the oxygen of this group is formed only in compound 8 (Fig. S53†). In compound 4, the acceptor of the hydrogen bond is the oxygen atom of the morpholinyl group. The resulting C(7) chain synthon arranges the molecules into a right-handed helix (Fig. S54†), leading to the chiral space group *P*2_1_2_1_2_1_ for this compound. In compound 3, in addition to the hydrazone nitrogen, the hydroxyl group is the second donor of hydrogen bonds. It forms an intramolecular hydrogen bond with the N2 nitrogen atom resulting in S(7) motif. It also acts as an acceptor in a bifurcated hydrogen bond with the hydrazone nitrogen N1 (Fig. S55†). A rather unusual R_2_^2^(4) homosynthon formed between two hydrazone NH groups occurs in compound 2. It is the reason for the largest distortion of the molecules of this compound, which increases the accessibility of the amino groups to each other. Moreover, each NH group form the second hydrogen bond with another nitrogen from the hydrazone moiety, resulting in R_2_^3^(5) (Fig. S56†).

The occurrence of π–π stacking interactions is exclusively observed in compounds that incorporate nitro groups (7–9), highlighting the pivotal contribution of these groups in the facilitation of such interactions (Table S6†). The molecules organize themselves into head-to-tail motifs. In compound 7, π⋯π stacking interactions propagate along the [010] crystallographic axis, forming supramolecular stacks, while in compounds 8 and 9, only π⋯π stacking dimers are formed (Fig. S57 and S58†).

Non-classical hydrogen bonds of various types, such as C–H⋯O, C–H⋯N, C–H⋯Cl, C–H⋯S, as well as C–H⋯π interactions, are common in all studied compounds. They are particularly numerous in compounds where neither classical hydrogen bonds nor π⋯π stacking interactions occur, *i.e.*, in compounds 1, 5, and 6. Anion⋯π interactions can also be observed between the nitro group and the thiophene ring in compound 10, or between the chlorine substituent at *ortho* position and the phenyl ring in compound 5 (Fig. S59 and S60†).

### 3.3 Analysis of absorption properties of phenylhydrazones

The theoretical UV-Vis spectra and experimental spectra demonstrate good agreement (Fig. S31–S50, Table S7†). This concordance is particularly evident in calculations considering the dominant chemical environment, where 2–4 molecules were used to account for predominant crystal structure interactions, (Fig. S41–S50†). Theoretical spectra obtained for single molecules showed satisfactory agreement in the higher energy range (shorter wavelengths, Fig. S31–S40†). However, they exhibited a lack of transitions for lower energy radiation (higher wavenumbers). Upon examining the spectra of individual compounds, several maxima can be distinguished, typically localized within the range of 200–500 nm. Compounds 1 and 4 are exceptions to this pattern, as their spectra primarily exhibit a single broad maximum within this range, without clear differentiation of individual bands.

Comparing the theoretical spectra calculated for single molecules with those for several molecules reveals interesting insights. For compounds 1 and 2, no significant differences in spectral patterns were observed between calculations using single molecules and those using multiple molecules. However, for these more complex systems (*i.e.*, calculations involving multiple molecules), a greater number of orbital transitions (oscillators) became apparent, although this did not result in additional visible bands in the spectra. Compound 3, in its complex system, showed a markedly improved match with the experimental spectrum, with numerous oscillators visible and transitions appearing around 400 nm, which were absent in the single-molecule calculations. Similar improvements were observed for compounds 4 and 5. For compound 6, the spectrum of the complex system was decidedly better, revealing transitions above 400 nm that were not visible in the simple system. Compound 7's theoretical spectrum significantly improved with calculations for the complex system, excellently reproducing the experimental spectrum. Notably, transitions in the 500–600 nm range appeared, which were absent in the simple system, along with many more visible oscillators. Similar improvements in spectral representation were observed for compounds 8 and 9. For compound 10, whose single-molecule spectrum significantly deviated from the experimental one due to a broad maximum extending to the end of the measurement range (800 nm), the theoretical spectrum for the complex system now shows much better agreement with experimental data. The theoretical spectrum for the complex system of compound 10 shows transitions up to 800 nm, accurately reflecting the experimental observations.

The orbital transitions in the studied compounds are predominantly π′′π* transitions (Fig. S61–S70†). Importantly, some transitions also exhibit Intermolecular Charge Transfer (ICT) characteristics, where the orbital electron density of the acceptor orbital is located on a different molecule of the compound. This phenomenon explains why calculations incorporating multiple molecules yield significantly improved theoretical spectra compared to single-molecule calculations. The inclusion of additional molecules in the computational model allows for the detection and accurate representation of these ICT transitions, which are crucial for understanding the complete electronic behavior of the system. Single-molecule calculations, by their nature, cannot capture these intermolecular effects, leading to the omission of important spectral features, particularly in the lower energy regions of the spectrum. Thus, the multi-molecule approach provides a more comprehensive and realistic picture of the electronic transitions occurring in these compounds, resulting in better agreement with experimental spectra and offering deeper insights into the underlying photophysical processes. Analyzing the overall absorption spectra, the contribution of ICT transitions in compounds 1 and 2 appears to be less dominant compared to the other studied systems, with this effect being particularly noticeable for compound 2. Nevertheless, ICT transitions are still observable for these compounds, especially at wavelengths above 300 nm. In the case of compound 3, the contribution of ICT transitions is more pronounced and easily identifiable across the entire analyzed spectral range, suggesting a stronger influence of these interactions on its optical properties. Here, the electron density of the acceptor orbital can extend even beyond the neighboring molecules. ICT transitions are also visible for the remaining systems (4–10). For compound 5, these transitions are particularly distinct and well-defined, with the electron density of the acceptor orbital localizing on the ring containing chlorine substituents rather than being distributed across the entire molecule. Notably, for compounds 4–6, ICT transitions occur mainly for excitations above 400 nm, while for compounds 7–10, these transitions also occur at lower wavelengths.

In reality, as clearly demonstrated by theoretical calculations, each absorption band corresponds to multiple orbital transitions, which are represented as oscillator strengths. This phenomenon underscores the complex nature of electronic transitions in these molecules and highlights the importance of theoretical computations in interpreting experimental UV-Vis spectra. The improved agreement between complex system calculations and experimental spectra further emphasizes the significance of considering intermolecular interactions in theoretical models, particularly in understanding ICT processes.

### 3.4 Analysis of fluorescent properties of phenylhydrazones

**Fig. 2:**
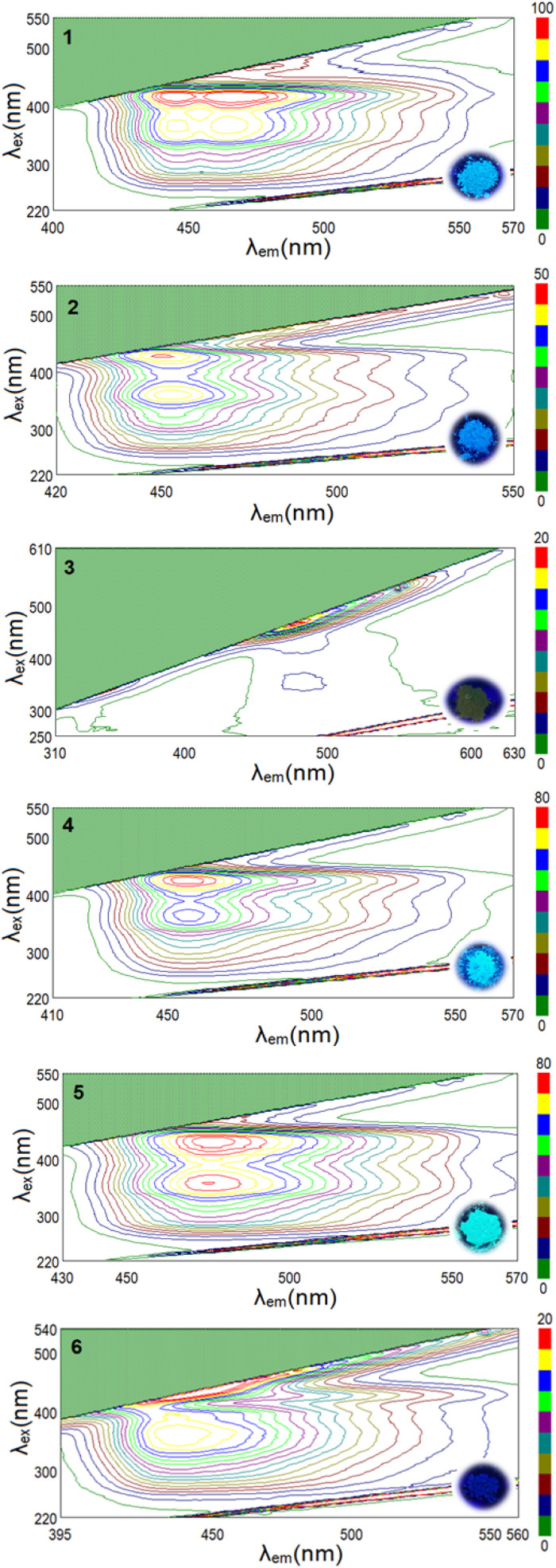
3D-EEM fluorescence spectra of the phenylhydrazones with the images of samples taken under UV lamp (*λ* = 365 nm). The fluorescence intensity colour code is displayed on the right side.

The fluorescent properties of the synthesized phenylhydrazones (1–10) were investigated using 2D and 3D solid-state fluorescence spectroscopy, revealing complex relationships between the molecular skeleton, the nature and position of substituents, and the emission characteristics (Fig. 2 and S71–S77†). The compounds exhibit diverse fluorescence behaviors, with emission wavelengths ranging from 438 to 482 nm, corresponding to the blue region of the electromagnetic spectrum. The molecular skeleton of phenylhydrazones plays a crucial role in determining the emission wavelength. Despite the introduction of various electron-donating and electron-withdrawing substituents, the position of the emission maximum showed only subtle changes, suggesting that the structure of the fluorophore core is the main factor influencing the emission wavelength. Notably, compounds containing nitro groups (7–10) proved to be an exception, displaying complete fluorescence quenching. While the core structure primarily affects the emission wavelength, substituents significantly influence the fluorescence intensity, which varies across a wide range (Table 1). The substituents' impact on fluorescence intensity and other detailed spectroscopic data are further discussed in the following sections of this paper.

**Fig. 3:**
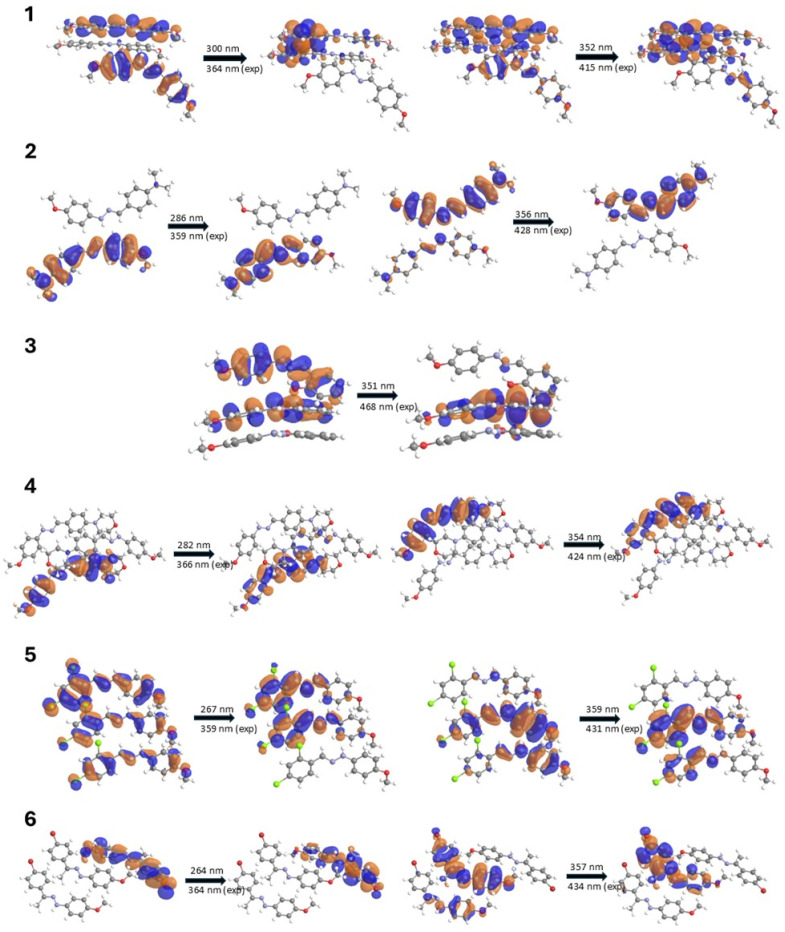
Calculated molecular orbital transition in the studied compounds (1–6), corresponding to the fluorescence emission maxima, is depicted with numerical values above the arrows indicating the calculated excitation maxima (nm) associated with each orbital transition.

To better understand the photophysical properties of the studied compounds in the solid state, quantum-mechanical calculations were performed for complex systems consisting of a molecule of the compound linked to neighboring molecules *via* dominant non-covalent interactions (Fig. 3 and S61–S70†). Analysis of the packing diagrams revealed significant intermolecular interactions, including π-ring overlap and interactions between electron-donating and electron-withdrawing groups. These interactions may indicate the possibility of intermolecular charge transfer (ICT), which could potentially influence the observed absorption and emission spectra. Calculations for multi-molecular systems revealed distinct spectral characteristics compared to single-molecule analyses. Compounds consisting of more than one molecule exhibited different absorption profiles, particularly in the higher wavelength region (400–800 nm). The computational results demonstrated that multi-molecular assemblies are characterized by altered spectral features, especially noticeable in the visible spectrum range. Such effects are present to varying degrees in all the studied phenylhydrazones but are particularly evident in compounds containing nitro groups. Analysis of the frontier orbital distribution revealed that both intramolecular and intermolecular charge transfer (ICT) occur in the studied compounds, contributing to their diverse fluorescence effects.

Compounds 1 and 5 exhibit particularly pronounced intramolecular charge transfer (ICT) effects in their fluorescence-related excitations, which correlate with their high fluorescence intensity. Analysis of the orbital electron density associated with these fluorescent transitions reveals interesting patterns. In compound 1, containing two methoxy groups, the electron density undergoes significant redistribution upon excitation, shifting towards the edges of the molecule where the methoxy groups are located. This donor effect enhances the stabilizing action of ICT in the excited state, minimizing non-radiative losses and favoring photon emission. Notably, compound 1 exhibited intense emission and four distinct fluorescence maxima, which is a remarkable feature among organic fluorophores. This behavior can be attributed to its unique molecular symmetry, facilitated by the presence of two methoxy groups on the phenyl core, and is reflected in the orbital electron density distribution associated with its fluorescent properties.

In compound 5, characterized by the presence of two chlorine atoms in the *para* and *ortho* positions on one ring and a methoxy group on the other, a similar effect is observed but directed mainly towards the chlorine-substituted ring. The chlorine atoms, acting as strong electron acceptors, further enhance charge separation during ICT, which favors the maintenance of fluorescence despite the electron-withdrawing nature of the substituents. The absence of strong hydrogen bonds in compounds 1 and 5 may be another factor contributing to their high fluorescence intensity. This is important because strong hydrogen bonds can induce an excited-state intramolecular proton transfer (ESIPT) mechanism, which leads to fluorescence quenching. The absence of this mechanism in compounds 1 and 5 may be one of the key reasons for their higher fluorescence compared to the other studied compounds.

For compounds 2–4 and 6, the electron density distribution in HOMO and LUMO encompasses the entire molecule, indicating a more delocalized character of the excited states. The presence of electron-donating groups, such as dimethylamino (2) and morpholinyl (4), increases the electron density in the π system, stabilizing the excited state and further promoting fluorescence. Despite their large, spatially demanding nature, these substituents enhance fluorescence by increasing the electron density in the π system of the molecule, stabilizing the excited state. Moreover, large groups restrict intramolecular rotation, reducing non-radiative deexcitation processes and favoring photon emission.

Compound 3, possessing a hydroxyl group, demonstrates the influence of intramolecular hydrogen bonding on fluorescence properties. The formation of an intramolecular hydrogen bond activates the excited-state intramolecular proton transfer (ESIPT) mechanism, leading to a characteristic bathochromic shift and longer emission wavelength. However, despite the observed Stokes shift, compound 3 exhibited the weakest fluorescence intensity among compounds 1–6, with only one distinct emission maximum.

The presence of electron-withdrawing groups, such as chloro (5) and bromo (6) substituents, introduced intriguing complexities in the fluorescence behavior. Compound 5 exhibited good fluorescence intensity despite the electron-withdrawing nature of its *ortho*- and *para*-chloro substituents. This observation can be rationalized by the redistribution of electron density in the chloro-substituted ring and the imine bond, enhancing fluorescence through anion⋯π interactions. In contrast, the heavy bromine atom in compound 6 led to a decrease in fluorescence intensity.

For compounds 7–10, containing strong electron-withdrawing nitro groups, fluorescence is completely quenched. The nitro substituents, acting as strong electron acceptors, significantly alter the electron density distribution in the molecule. Quantum-mechanical calculations for multi-molecular systems revealed a significant shift of electron density from the π system towards the nitro groups, which is particularly evident compared to calculations for single molecules. This uneven distribution of electrons destabilizes the excited state and promotes non-radiative deactivation pathways. Additionally, the presence of π⋯π interactions between the phenylene rings in compounds 7–9 might contribute to the quenching effect through an aggregation-caused quenching (ACQ) mechanism. It is worth noting that while ICT generally enhances fluorescence in compounds 1–6, in the case of compounds with nitro groups (7–10), the same process leads to the formation of states that prefer non-radiative deactivation. This is due to a combination of the strong electron-withdrawing properties of nitro groups, the possibility of rapid internal conversion, increased spin–orbit coupling (SOC),^59^ and the potential formation of “dark” excited states that effectively compete with the fluorescence emission process. In nitro compounds, the presence of the nitro group enhances SOC, which in turn increases the rate of intersystem crossing (ISC). ISC can quench fluorescence by facilitating the transition of molecules from the singlet excited state to the triplet excited state. Once in the triplet state, fluorescence emission is inhibited, as the triplet excited state typically has a longer lifetime and can undergo other deactivation processes. Collectively, these processes lead to the dissipation of excitation energy through non-radiative pathways, resulting in fluorescence quenching. Analysis of the charge distribution in compounds 7 and 9, particularly in the context of =N–N– bonds, reveals significant differences compared to other molecules, with larger charge differences on the nitrogen atoms (Δ*q* = 0.330 for compound 7 and Δ*q* = 0.261 for compound 9). These observations highlight the complexity of electronic interactions in the studied compounds and their impact on fluorescence properties. The fluorescence properties of the studied phenylhydrazones result from the complex interplay of multiple factors, including the structure of the molecular skeleton, the nature and position of substituents, ICT effects (both intra- and intermolecular), ESIPT mechanisms, and intermolecular interactions.

The fluorescence quantum yield values, presented in Table 1, show systematic variations among the studied compounds. Compound 1 demonstrates the highest quantum yield (*ϕ*_f_ = 0.24) when excited at 364 nm, which is notably higher than its quantum yield at 415 nm excitation (*ϕ*_f_ = 0.12). Similarly, good quantum yields were observed for compounds 2 (*ϕ*_f_ = 0.13), 4 (*ϕ*_f_ = 0.18), and 5 (*ϕ*_f_ = 0.19) when excited in the UV region. The quantum efficiency under UV excitation maintains higher values throughout the series, following the expected relationship between excitation energy and emission efficiency. As typically observed in such systems, excitation at higher energies leads to more efficient population of excited states and fewer non-radiative deactivation pathways compared to longer wavelength excitation. These hydrazone derivatives exhibit notable solid-state quantum yields, comparable to previously reported analogues,^77,78^ despite common fluorescence quenching effects typically observed in solid state.

## 4 Conclusions

Our comprehensive investigation into the synthesis, structural characterization, and photophysical properties of a series of phenylhydrazones has revealed complex relationships between molecular structure, substituent effects, and the resulting fluorescence behavior. The molecular skeleton of phenylhydrazones plays a pivotal role in determining emission wavelengths, with the core structure being the primary factor influencing photophysical properties. The introduction of various electron-donating and electron-withdrawing substituents has a limited impact on the position of emission maxima, which for the studied compounds falls within the blue light range (438–482 nm). However, substituents do affect fluorescence intensity and quantum yields. The absolute quantum yield values demonstrate excellent correlation with relative fluorescence intensities, confirming the reliability of the observed photophysical trends.

UV-Vis spectroscopy, combined with quantum mechanical calculations, proved instrumental in elucidating the absorption properties of the studied compounds. However, this work highlights a crucial finding: calculations for isolated molecules failed to yield satisfactory results, underscoring the critical role of the chemical environment in accurately predicting molecular absorption characteristics. Our study reveals that incorporating dominant intermolecular interactions into the calculations significantly improved the agreement between theoretical and experimental UV-Vis spectra. This refined approach enabled precise identification of π → π* orbital transitions and intramolecular charge transfer (ICT) processes. Notably, it provided insights into the pronounced ICT effects in compounds 1 and 5, correlating with their high fluorescence intensity, and elucidated the more delocalized character of excited states in compounds 2–4 and 6. The methodology employed here offers a pragmatic alternative to complex and computationally expensive periodic calculations, which are often impractical for systems like those presented in this work. By considering the chemical environment, we gained a deeper understanding of how electron-donating groups stabilize excited states and promote fluorescence, while also explaining the complete fluorescence quenching in nitro-containing compounds (7–10) due to non-radiative deactivation pathways. This approach proves particularly valuable when computational results are unsatisfactory, especially in the higher wavelength range. It demonstrates that accounting for the dominant intermolecular interactions can significantly enhance our ability to predict and interpret the photophysical properties of complex molecular systems without resorting to more advanced computational techniques. For compounds 2–4 and 6, the electron density distribution in relevant donor and acceptor orbitals encompasses the entire molecule, indicating a more delocalized character of excited states. The presence of electron-donating groups increases electron density in the π system, effectively modifying the energies and spatial distribution of donor orbitals. This modification stabilizes the excited state and promotes fluorescence. The effect is particularly pronounced in compounds containing substituents such as dimethylamino and morpholinyl groups, which not only enhance fluorescence by altering the electronic structure but also restrict intramolecular rotation, further contributing to emission intensity. Compounds containing a combination of electron-donating and electron-withdrawing substituents exhibit diverse fluorescence properties. The unique molecular symmetry and efficient excited state stabilization, facilitated by the presence of methoxy groups on the phenyl core, result in intense emission and multiple distinct fluorescence maxima. These observations can be attributed to the complex interplay between donor and acceptor orbitals, leading to efficient charge transfer processes. Intramolecular hydrogen bonding activates the excited-state intramolecular proton transfer (ESIPT) mechanism, leading to bathochromic shift and longer emission wavelength. This process involves significant reorganization of electron density in both donor and acceptor orbitals upon excitation. The introduction of electron-withdrawing substituents adds complexity to the fluorescence behavior by modifying the acceptor orbitals. Chloro substituents, for instance, exhibit good fluorescence intensity due to anion⋯π interactions, while bromo substituents show reduced intensity due to the heavy atom effect. These observations highlight the nuanced impact of halogen substituents on the electronic structure and subsequent photophysical properties of these compounds.

In stark contrast, compounds containing strongly electron-withdrawing nitro groups (7–10) exhibit complete fluorescence quenching. Quantum mechanical calculations revealed that nitro substituents significantly alter the electron density distribution across the molecule's donor and acceptor orbitals, destabilizing the excited state and promoting non-radiative deactivation pathways. This effect is further compounded by π⋯π interactions between phenylene rings, which contribute to the quenching effect through the aggregation-induced quenching (ACQ) mechanism. These findings underscore the complex interplay between molecular structure, substituent effects, and the resulting modifications to donor and acceptor orbitals in determining the fluorescence properties of phenylhydrazones. The ability to fine-tune these properties through strategic molecular design opens up exciting possibilities for developing tailored luminescent materials for various applications.

## Data availability

All data generated or analyzed during this study are included in this published article and its ESI files.†

## Author contributions

Conceptualization: Sobczak, P. and Trzęsowska-Kruszyńska, A.; investigation, formal analysis: Sobczak, P., Sierański, T., Świątkowski, M., Kolińska, J.; visualization, writing—original draft preparation, writing—review and editing: Sobczak, P., Sierański, T., Świątkowski, M.; supervision: Trzęsowska-Kruszyńska, A.

## Conflicts of interest

There are no conflicts to declare.

## Supplementary Material

RA-015-D4RA07856J-s001

RA-015-D4RA07856J-s002
