# Supplementary material for: Unraveling the structure–property relationships in fluorescent phenylhydrazones: the role of substituents and molecular interactions
Source: RSC Adv. 2025 Jan 17;15(3):1514–26. doi: 10.1039/d4ra07856j (PMC11740018; doi:10.1039/d4ra07856j)
Supplement: RA-015-D4RA07856J-s001 [file RA-015-D4RA07856J-s001.pdf]

Supplementary Information (SI) for RSC Advances.
This journal is © The Royal Society of Chemistry 2025

Supplementary Information

Unraveling the Structure-Property Relationships in Fluorescent Phenylhydrazones: The Role of Substituents and Molecular Interactions

Paulina Sobczak [a,b], Tomasz Sierański [a], Marcin Świątkowski [a], Agata Trzęsowska-Kruszyńska [a] and Jolanta Kolińska[c]

a *Institute of General and Ecological Chemistry, Lodz University of Technology, Zeromskiego 116, 90924, Lodz, Poland*
b *Łukasiewicz – Lodz Institute of Technology, M. Sklodowskiej-Curie 19/27, 90570 Lodz, Poland*

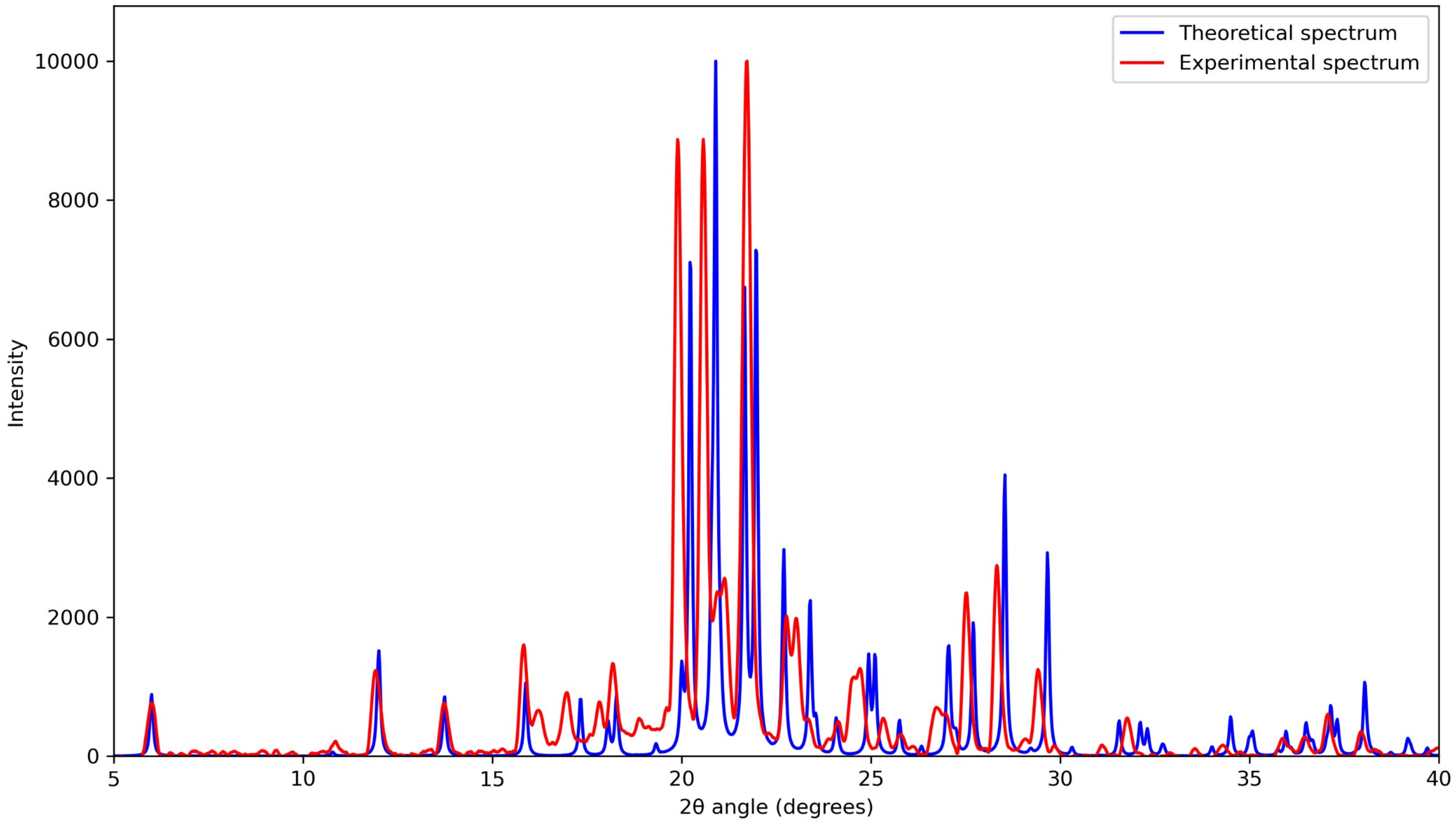

Figure S1. X-ray powder diffraction data of (*E*)-1-(4-methoxybenzylidene)-2-(4-methoxyphenyl)hydrazine (**1**).

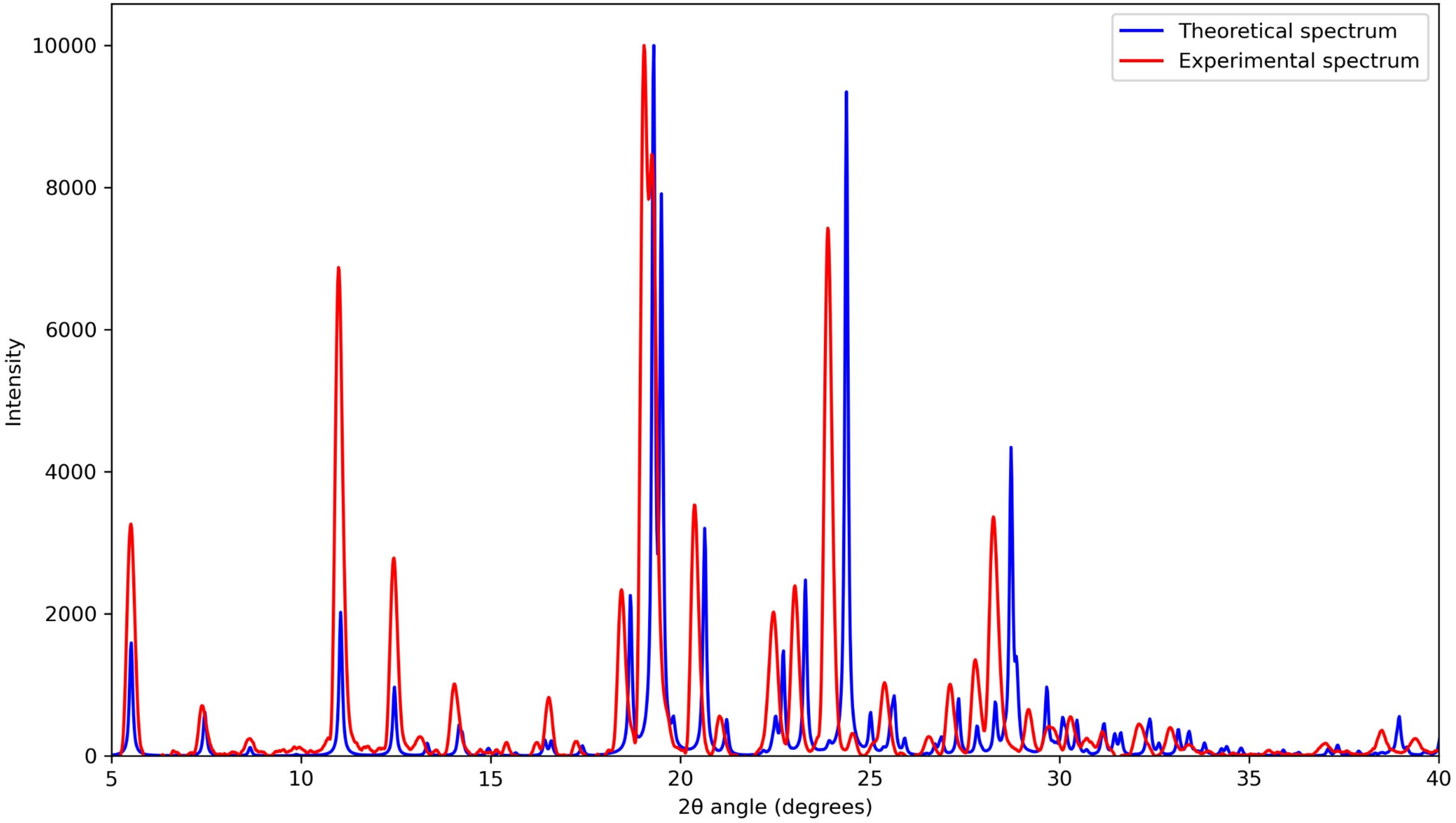

Figure S2. X-ray powder diffraction data of (*E*)-4-((2-(4-methoxyphenyl)hydrazineylidene)methyl)-*N*,*N*-dimethylaniline (**2**).

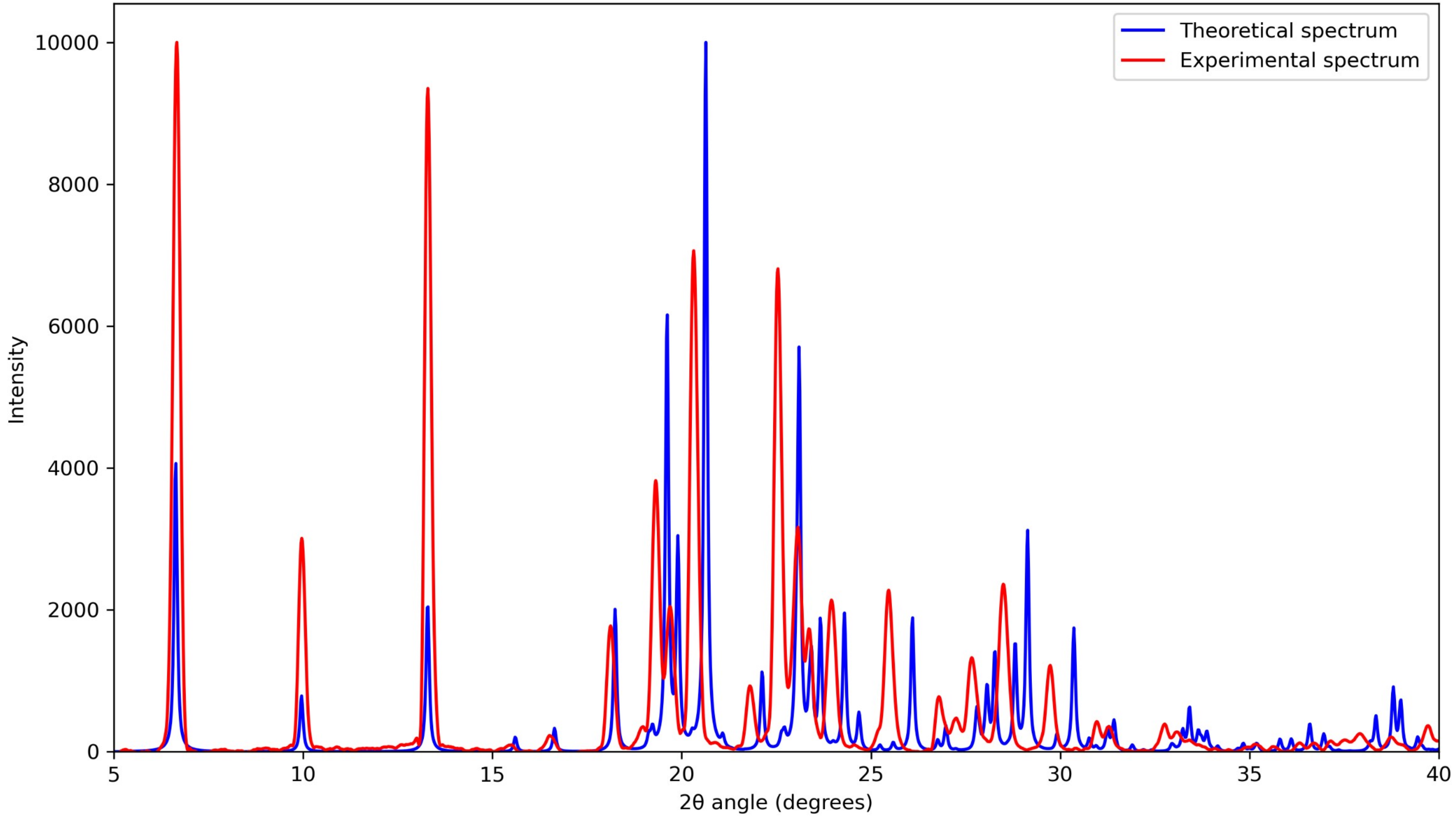

Figure S3. X-ray powder diffraction data of (*E*)-2-((2-(4-methoxyphenyl)hydrazineylidene)methyl)phenol (**3**).

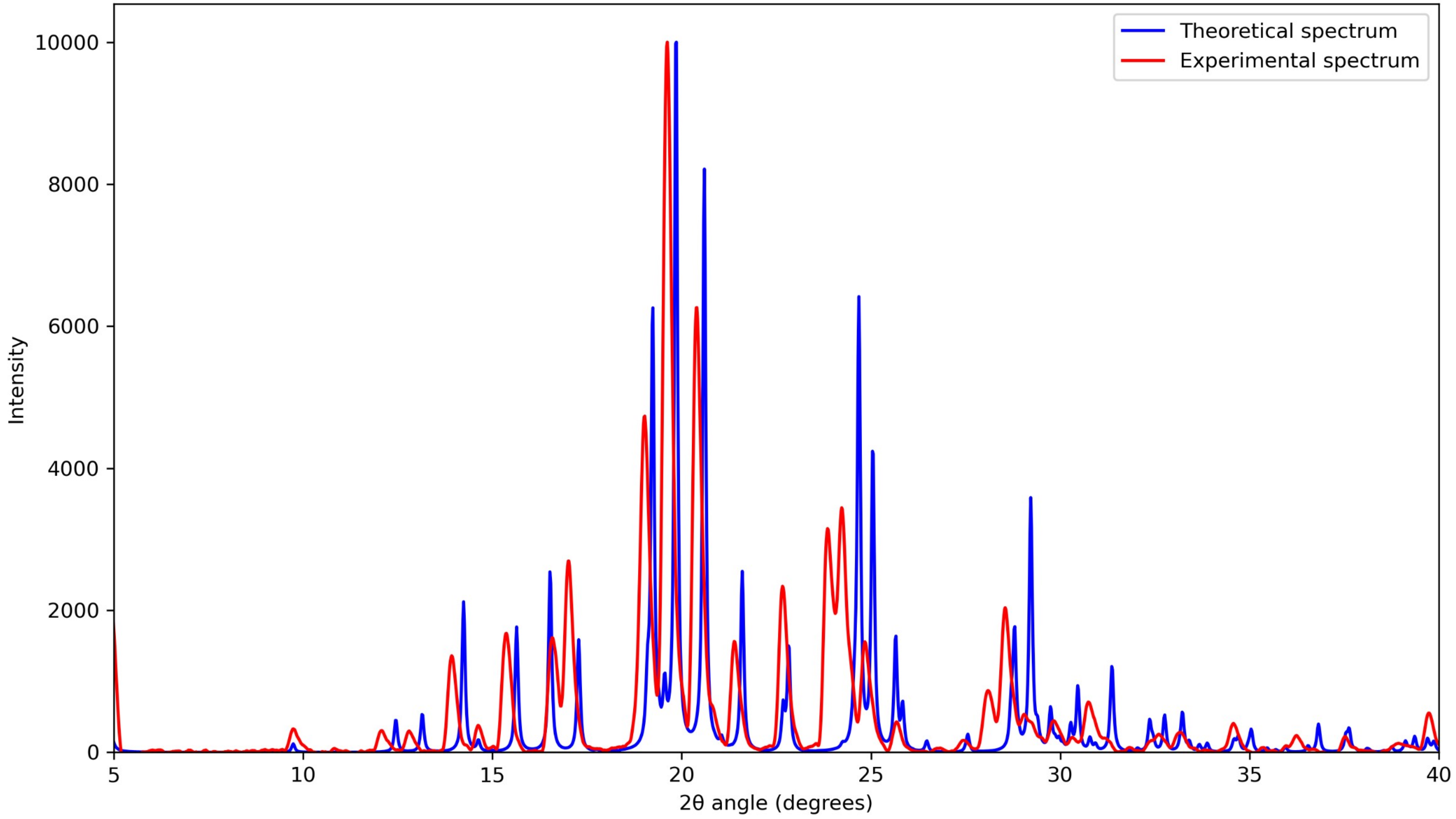

Figure S4. X-ray powder diffraction data of (*E*)-4-(4-((2-(4-methoxyphenyl)hydrazineylidene)methyl)phenyl)morpholine (**4**).

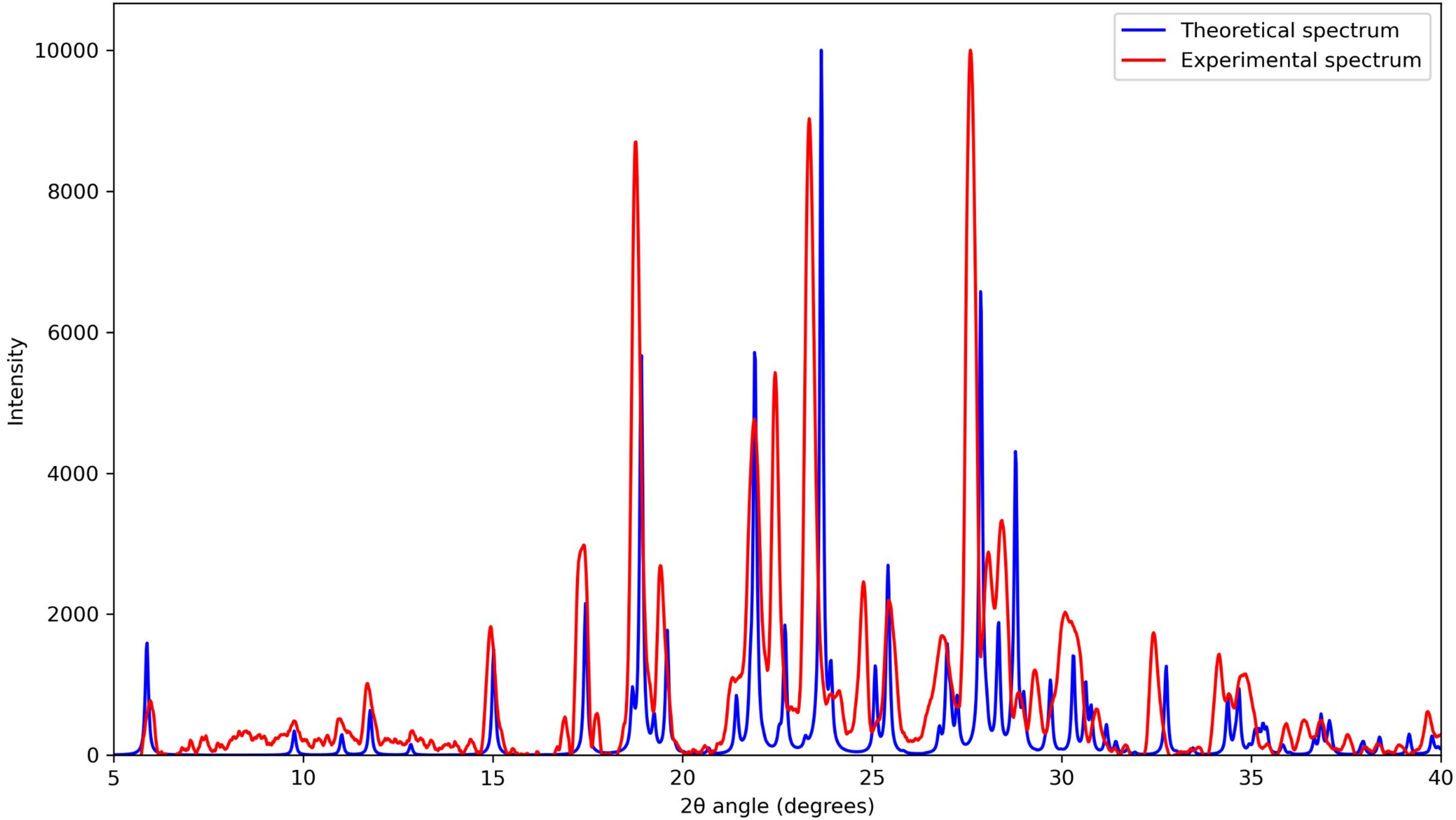

Figure S5. X-ray powder diffraction data of (*E*)-1-(2,4-dichlorobenzylidene)-2-(4-methoxyphenyl)hydrazine (**5**).

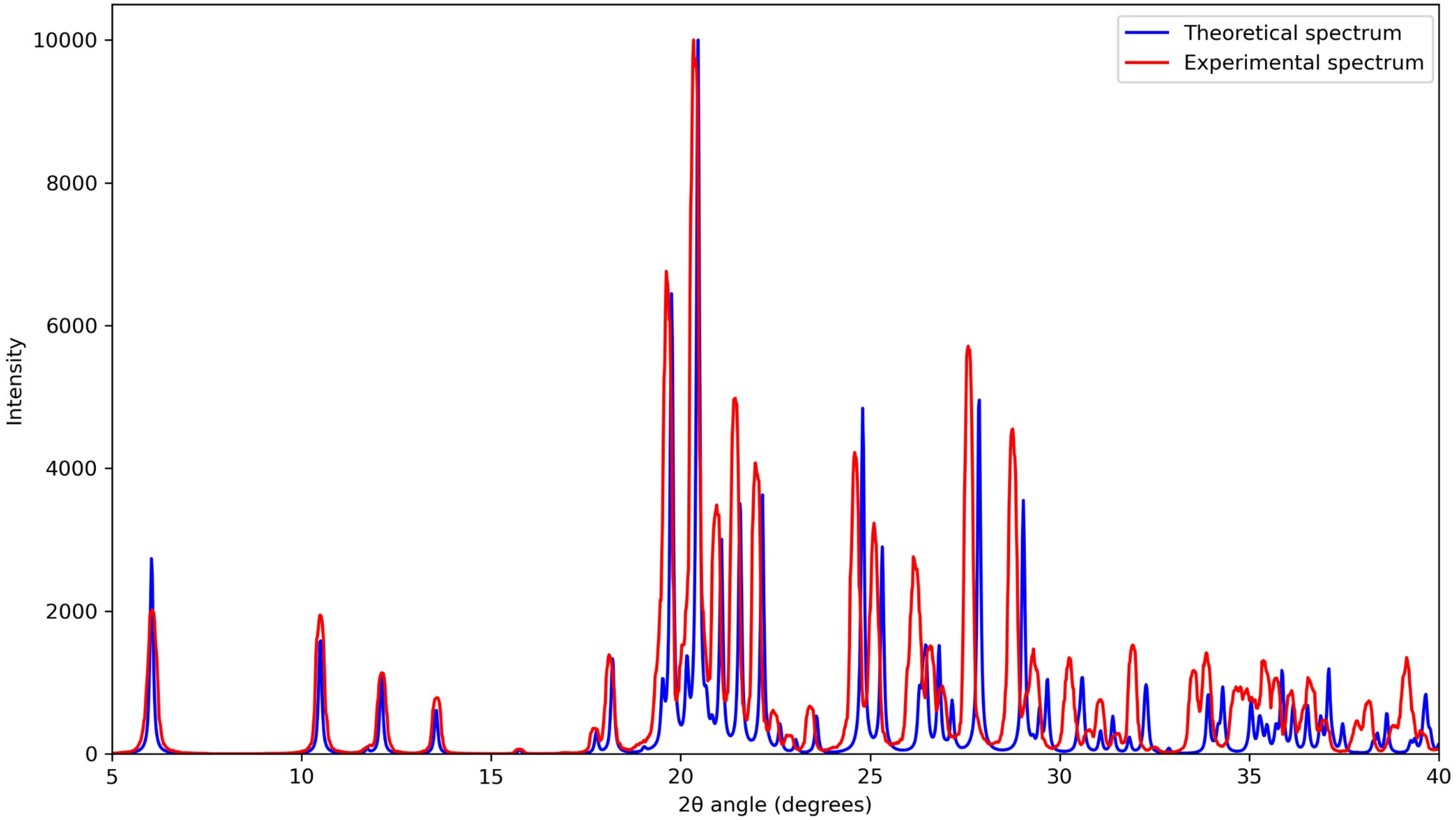

Figure S6. X-ray powder diffraction data of (*E*)-1-(1-(4-bromophenyl)ethylidene)-2-(4-methoxyphenyl)hydrazine (**6**).

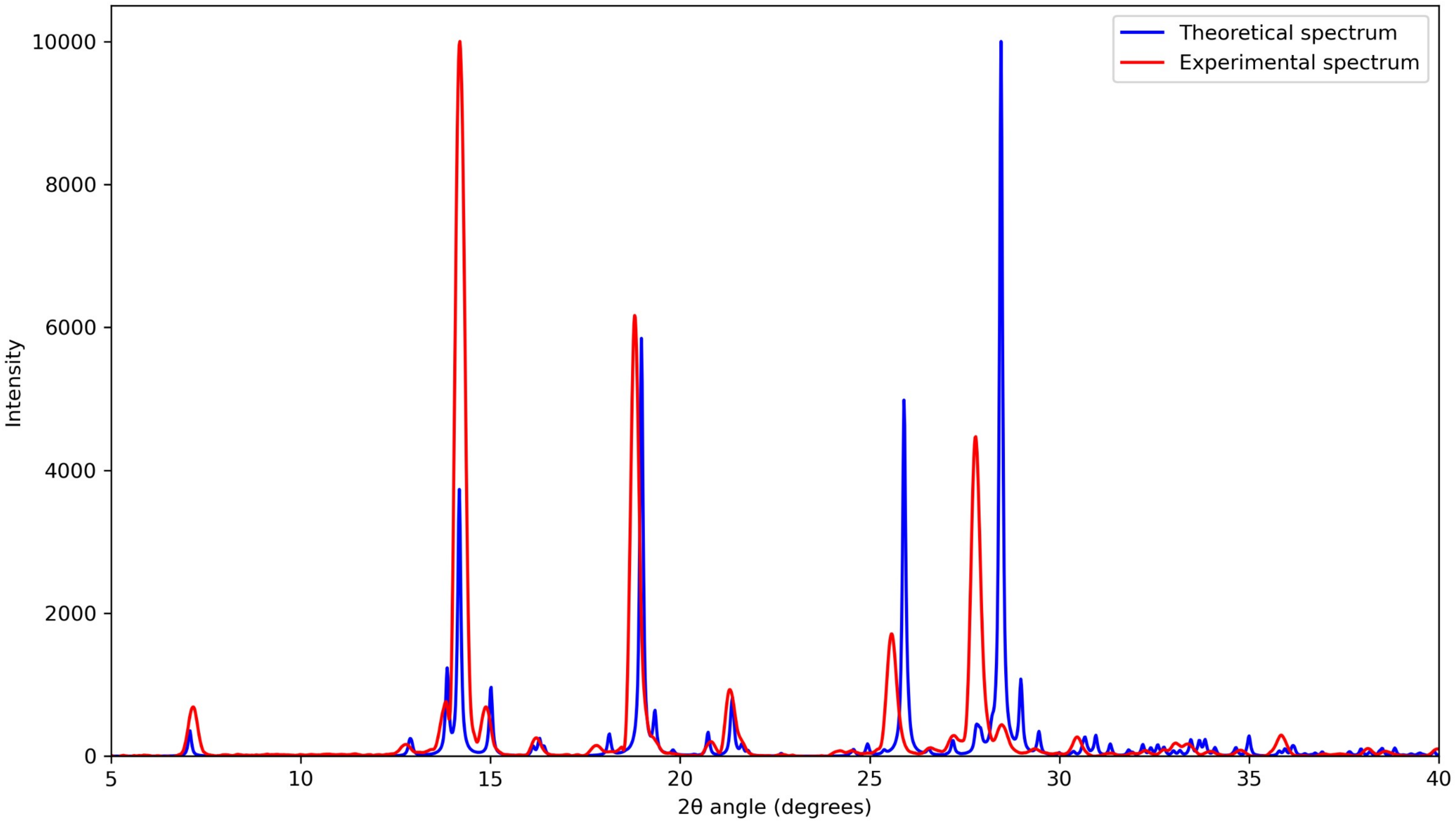

Figure S7. X-ray powder diffraction data of (*E*)-1-(4-methoxyphenyl)-2-((5-nitrofuran-2-yl)methylene)hydrazine (**7**).

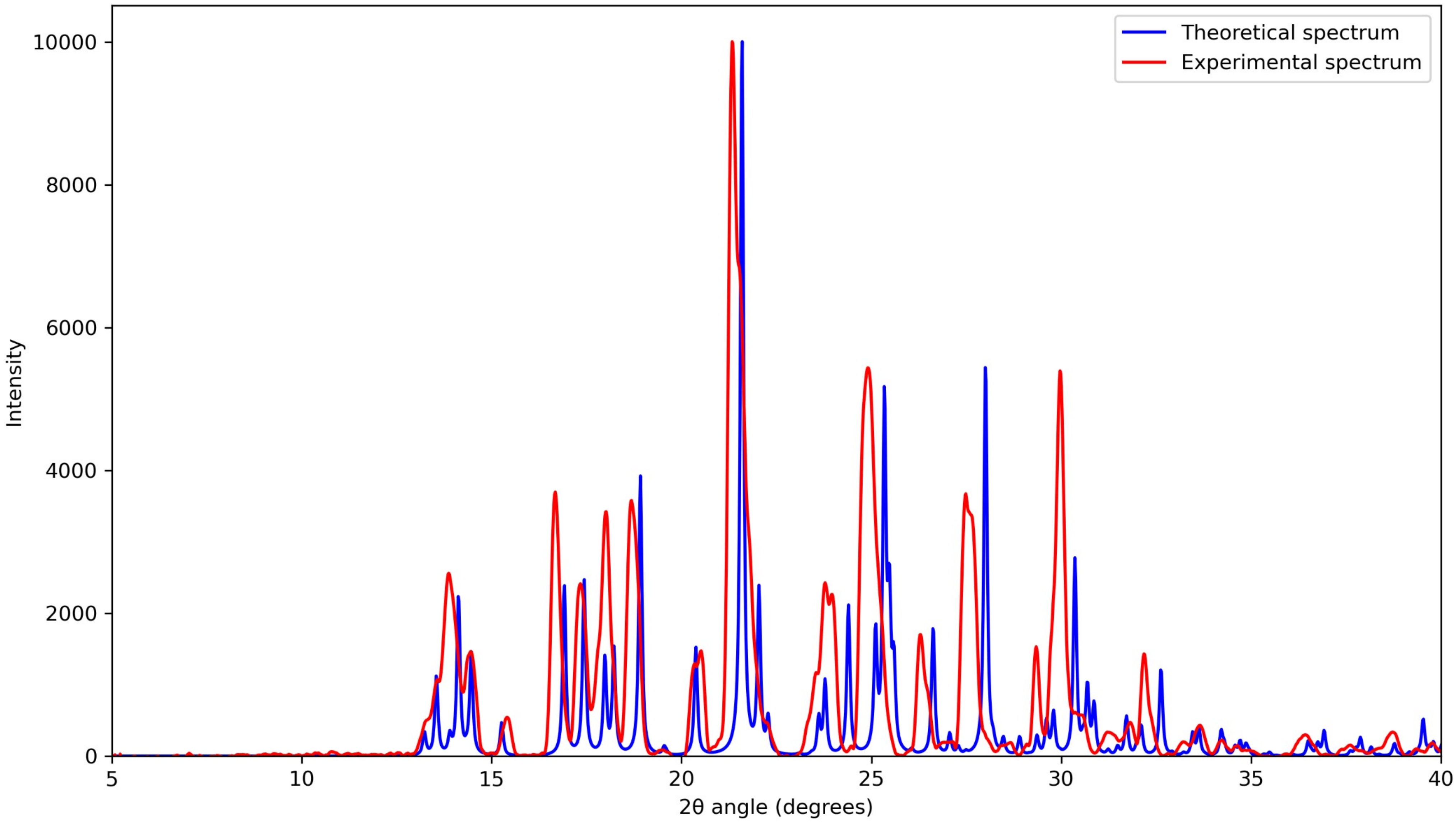

Figure S8. X-ray powder diffraction data of (*E*)-1-(4-methoxyphenyl)-2-(4-nitrobenzylidene)hydrazine (**8**).

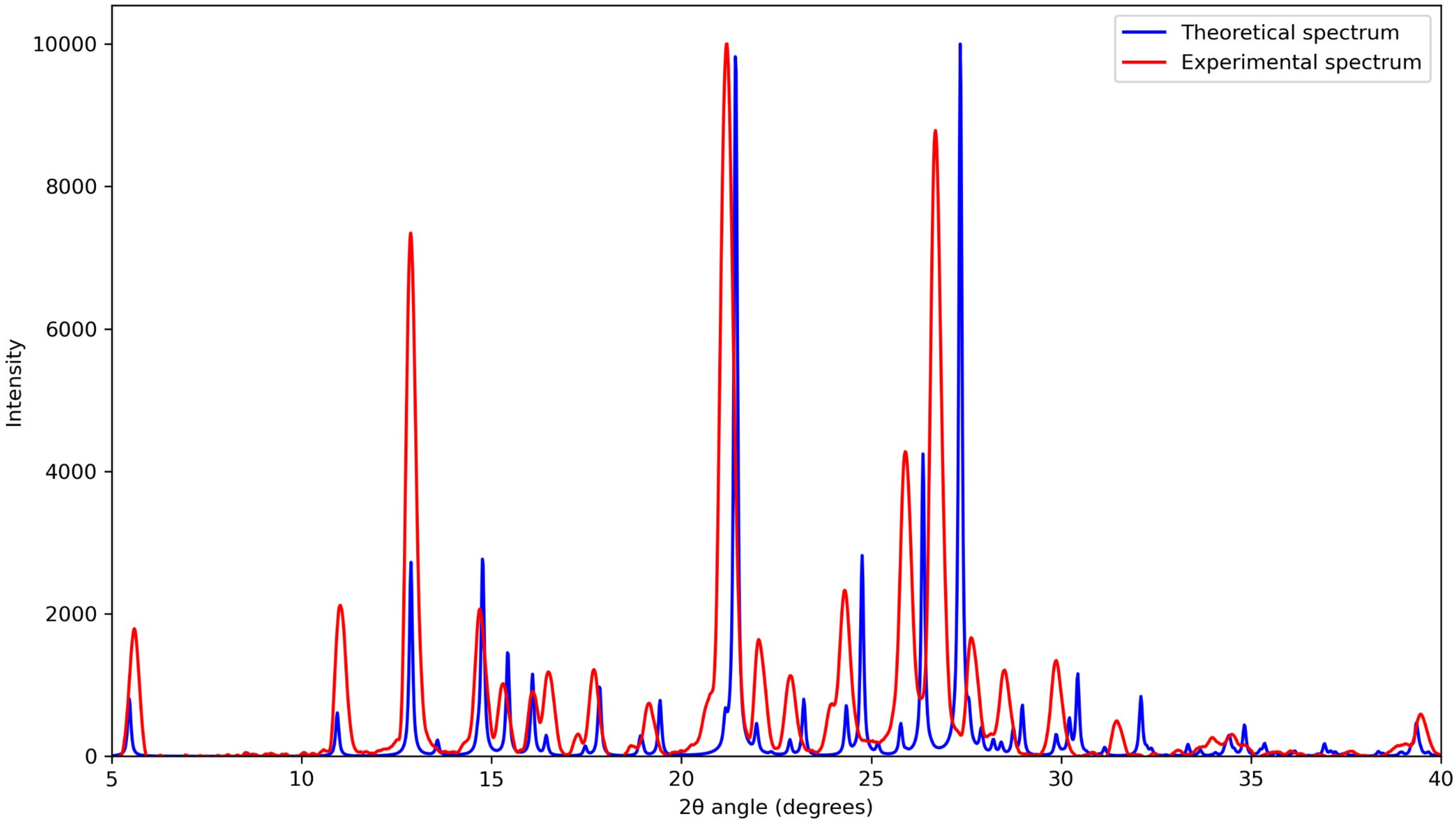

Figure S9. X-ray powder diffraction data of (*E*)-1-(4-methoxyphenyl)-2-(3-(4-nitrophenyl)propylidene)hydrazine (**9**).

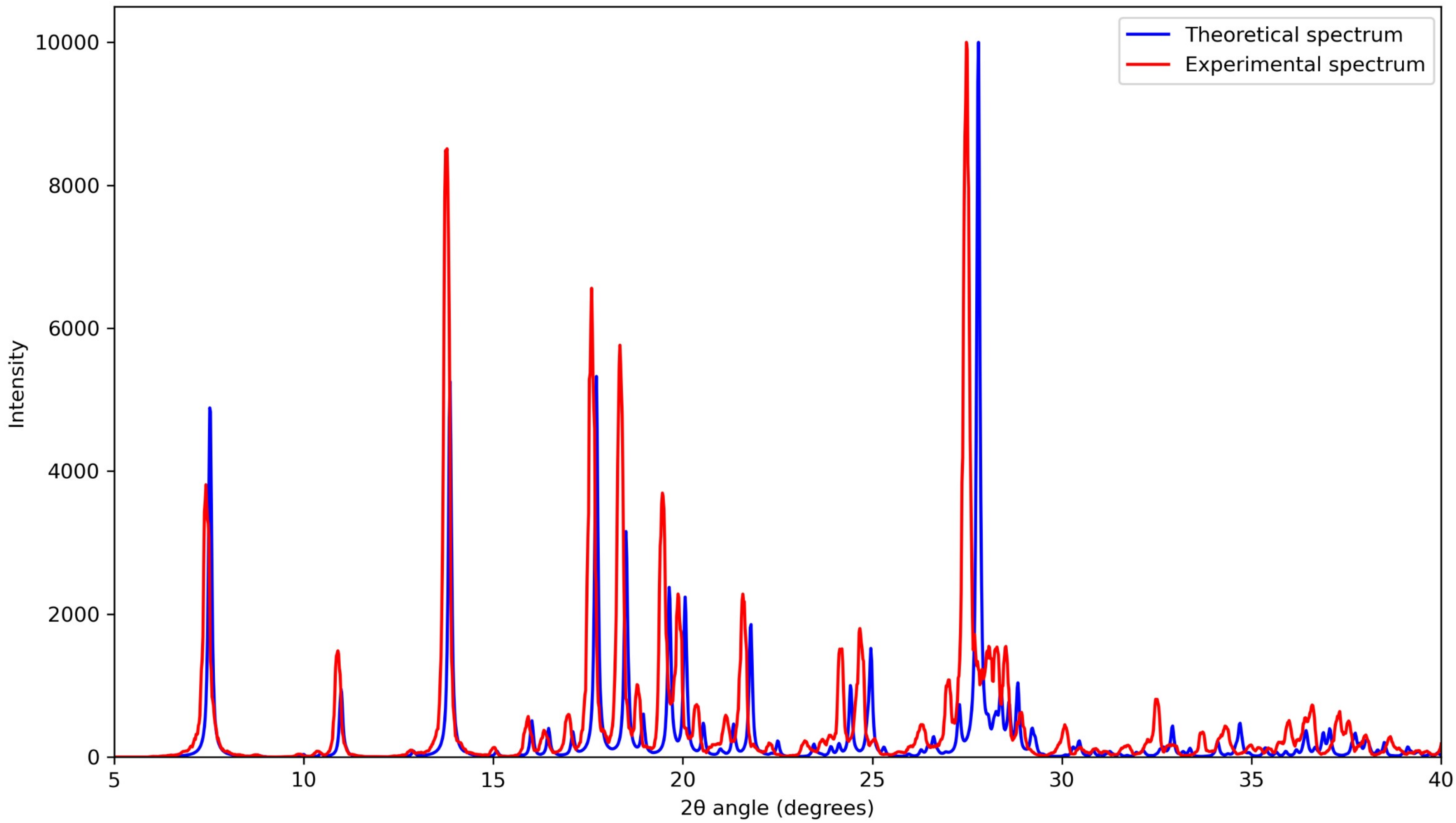

Figure S10. X-ray powder diffraction data of (*E*)-1-(4-methoxyphenyl)-2-((5-nitrothiophen-2-yl)methylene)hydrazine (**10**).

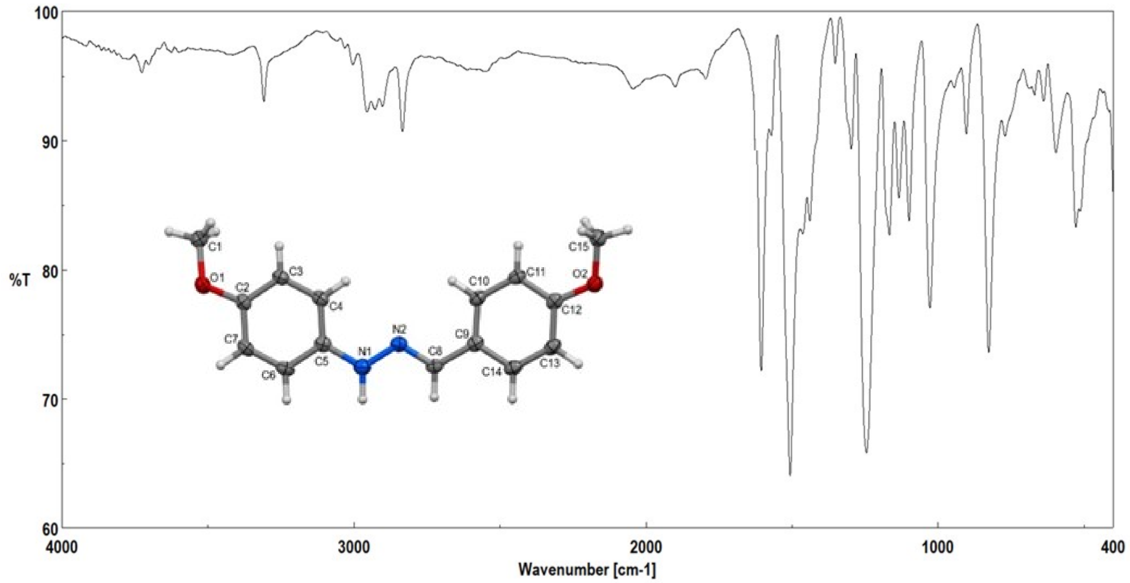

Figure S11. IR spectrum of (*E*)-1-(4-methoxybenzylidene)-2-(4-methoxyphenyl)hydrazine (**1**).

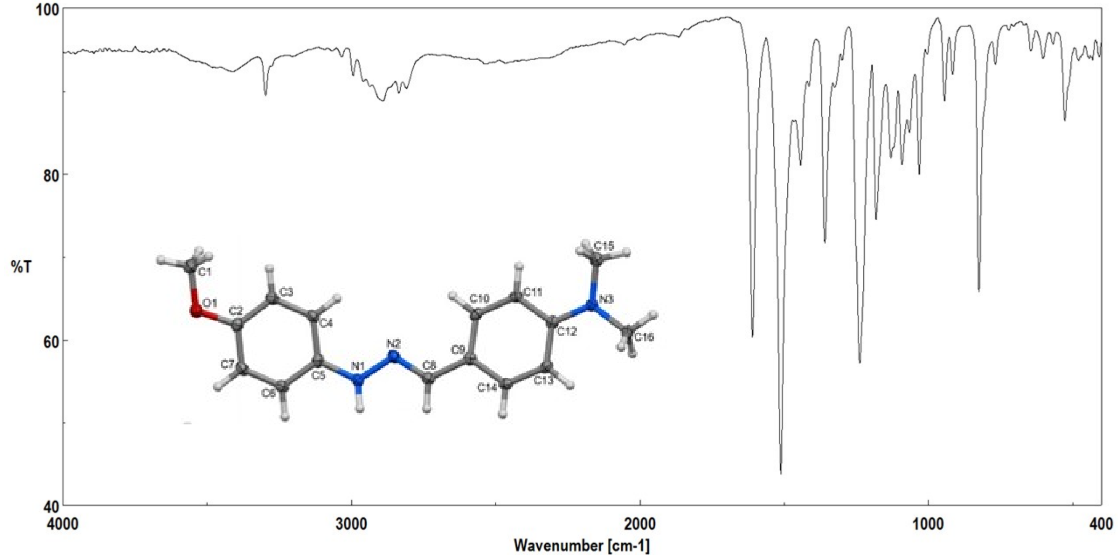

Figure S12. IR spectrum of (*E*)-4-((2-(4-methoxyphenyl)hydrazineylidene)methyl)-*N*,*N*-dimethylaniline (**2**).

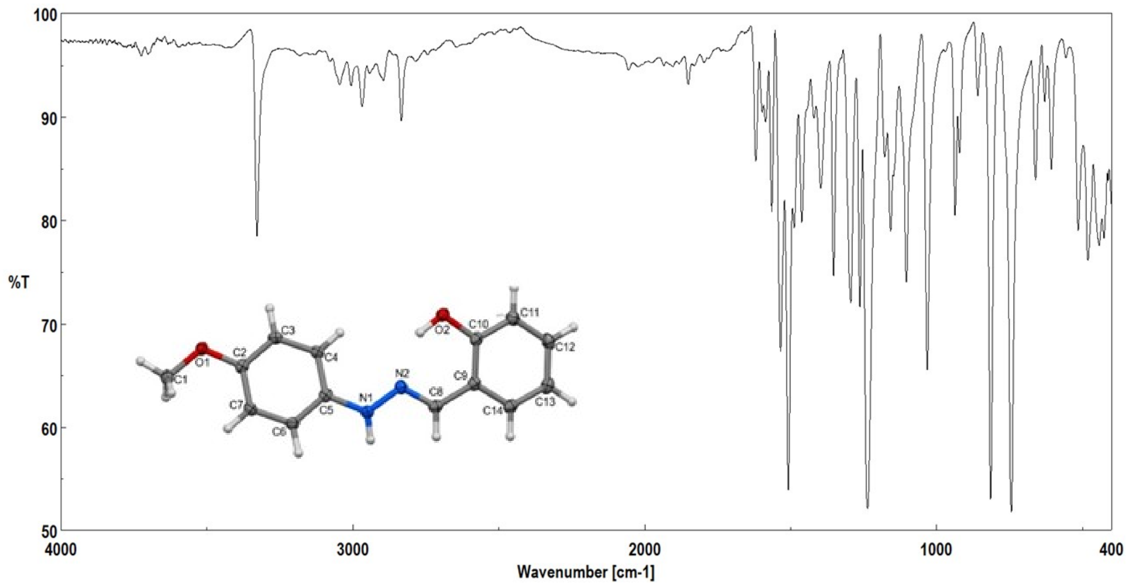

Figure S13. IR spectrum of (*E*)-2-((2-(4-methoxyphenyl)hydrazineylidene)methyl)phenol (**3**).

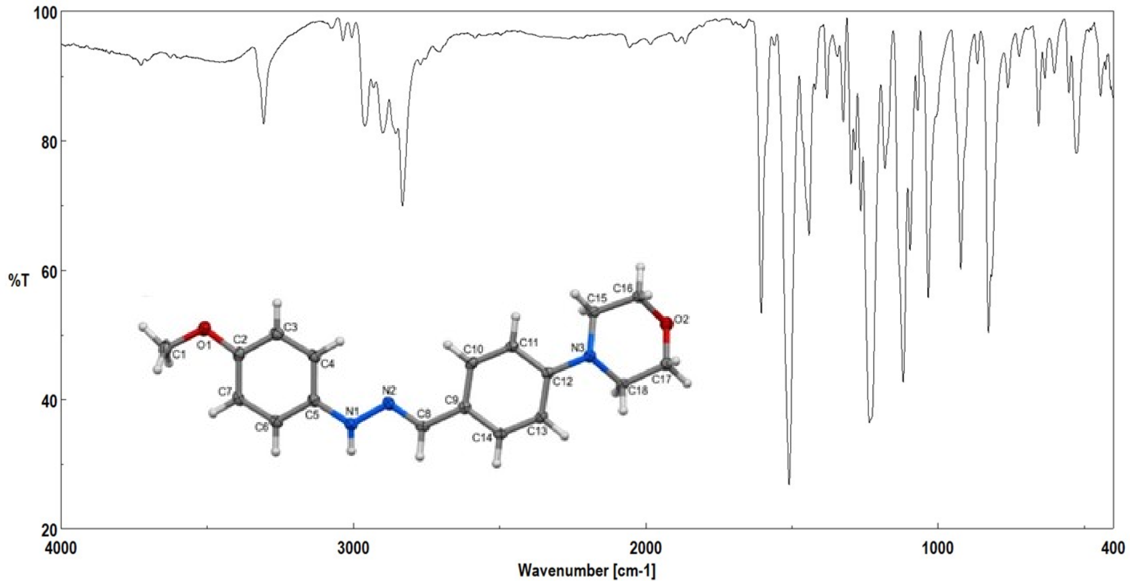

Figure S14. IR spectrum of (*E*)-4-(4-((2-(4-methoxyphenyl)hydrazineylidene)methyl)phenyl)morpholine (**4**).

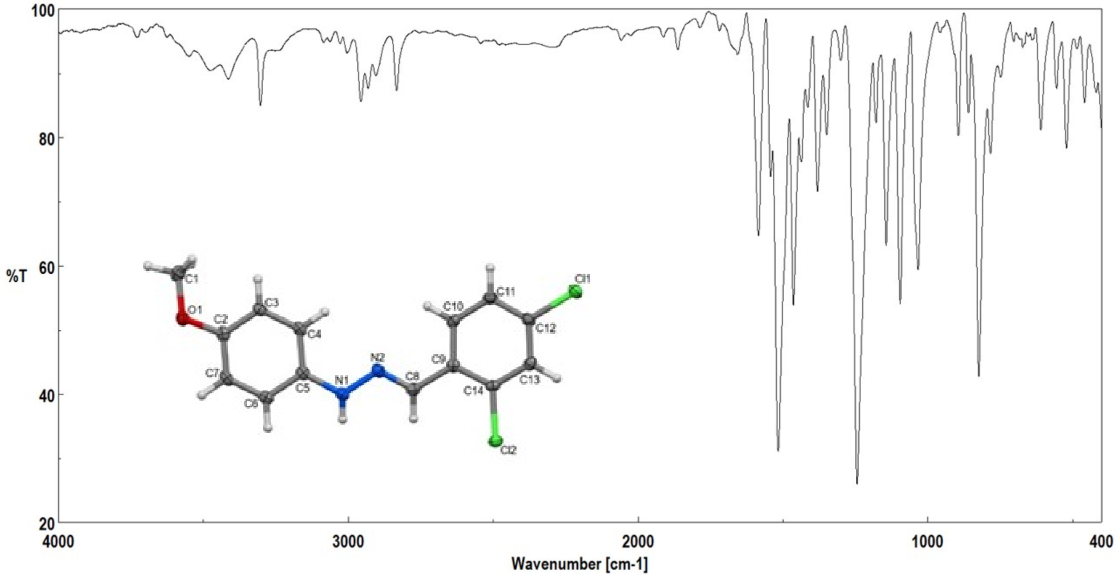

Figure S15. IR spectrum of (*E*)-1-(2,4-dichlorobenzylidene)-2-(4-methoxyphenyl)hydrazine (**5**).

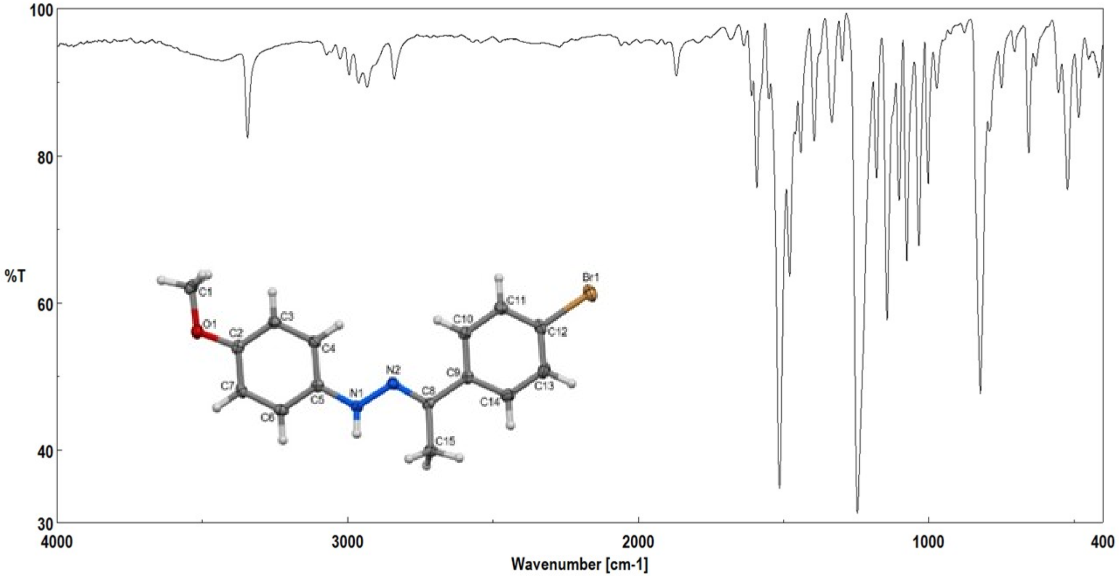

Figure S16. IR spectrum of (*E*)-1-(1-(4-bromophenyl)ethylidene)-2-(4-methoxyphenyl)hydrazine (**6**).

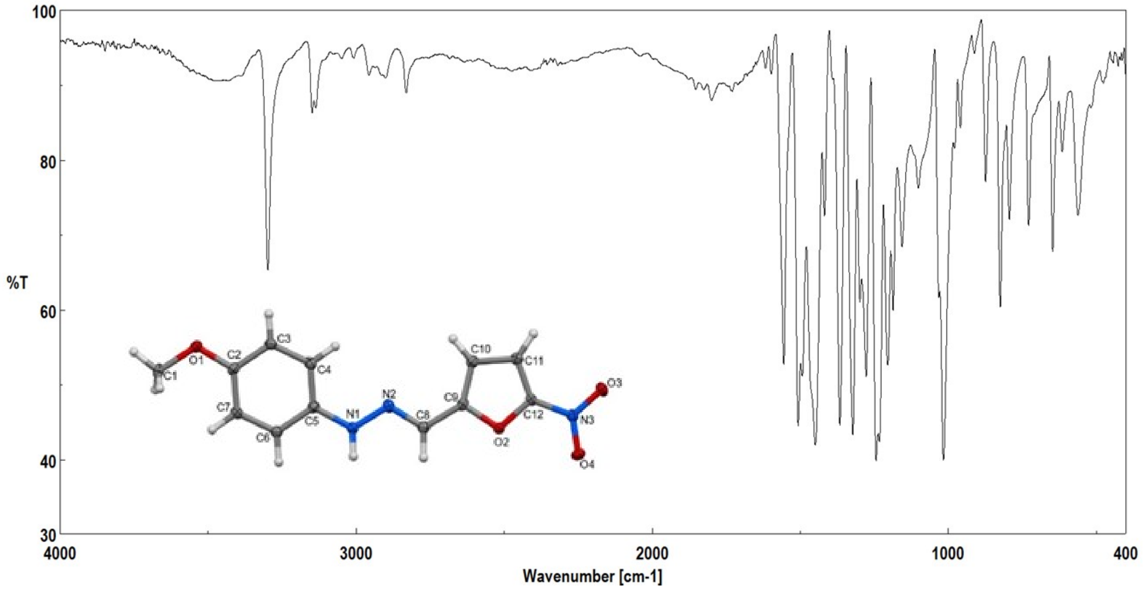

Figure S17. IR spectrum of (*E*)-1-(4-methoxyphenyl)-2-((5-nitrofuran-2-yl)methylene)hydrazine (**7**).

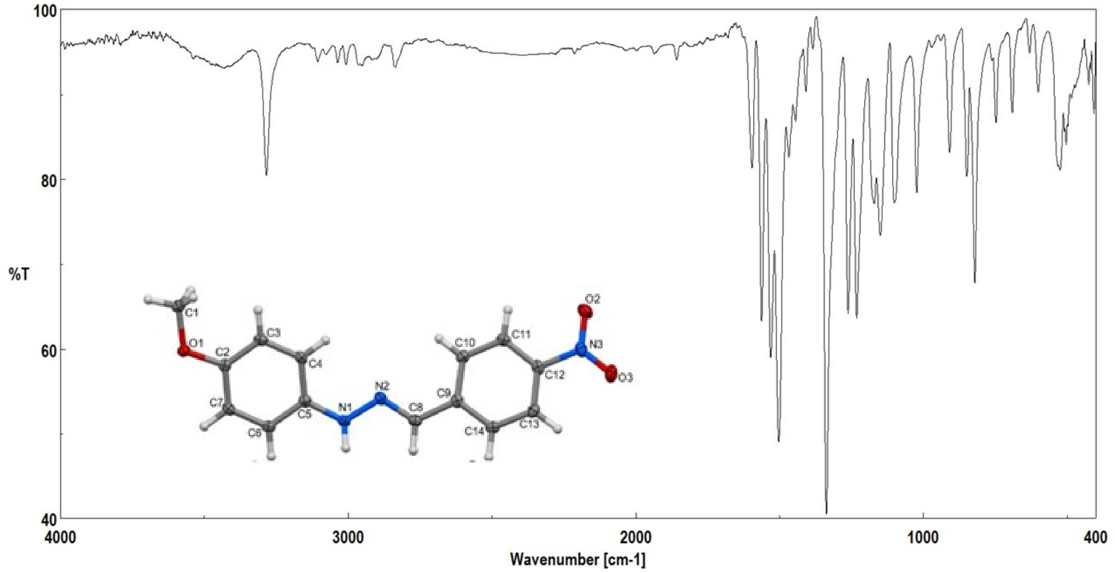

Figure S18. IR spectrum of (*E*)-1-(4-methoxyphenyl)-2-(4-nitrobenzylidene)hydrazine (**8**).

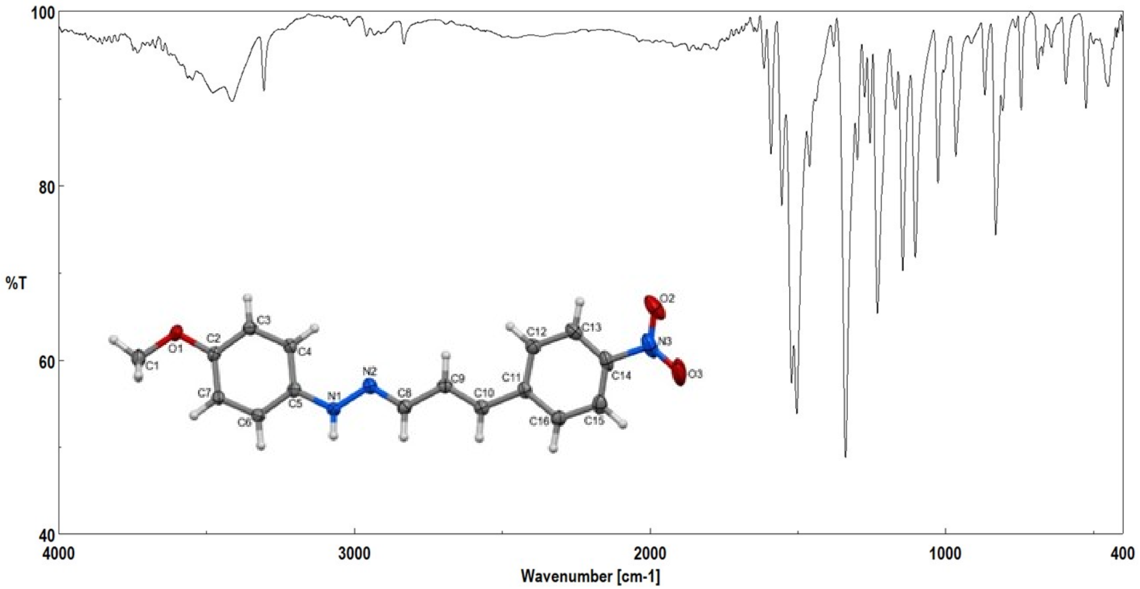

Figure S19. IR spectrum of (*E*)-1-(4-methoxyphenyl)-2-(3-(4-nitrophenyl)propylidene)hydrazine (**9**).

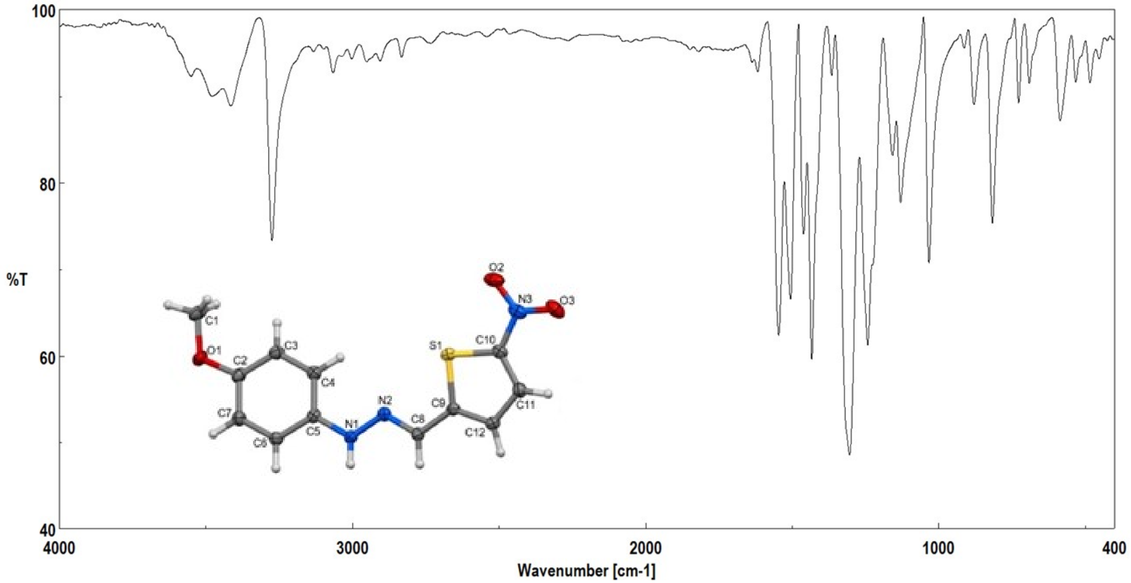

Figure S20. IR spectrum of (*E*)-1-(4-methoxyphenyl)-2-((5-nitrothiophen-2-yl)methylene)hydrazine (**10**).

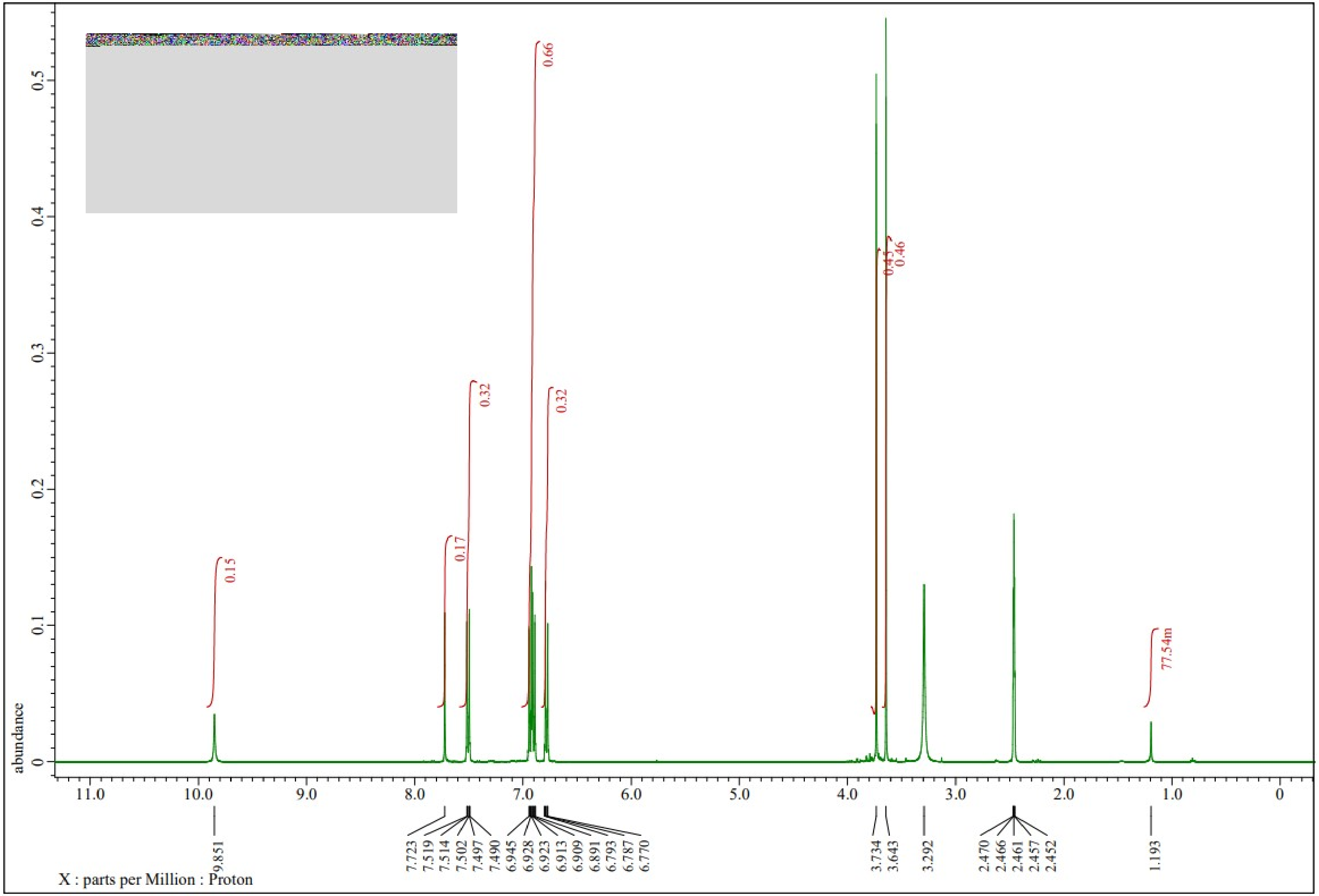

Figure S21. NMR spectrum of (*E*)-1-(4-methoxybenzylidene)-2-(4-methoxyphenyl)hydrazine (**1**).

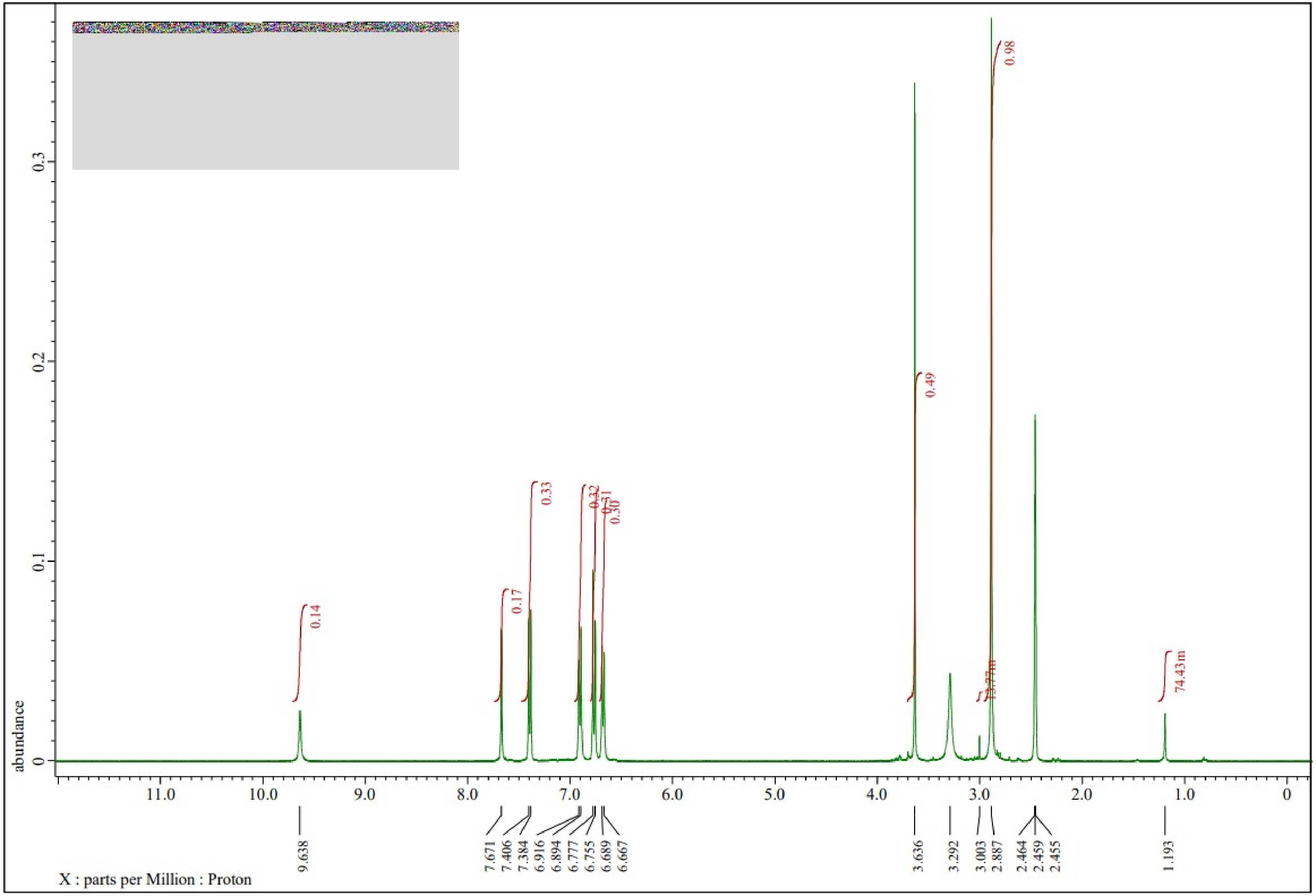

Figure S22. NMR spectrum of (*E*)-4-((2-(4-methoxyphenyl)hydrazineylidene)methyl)-*N*,*N*-dimethylaniline (**2**).

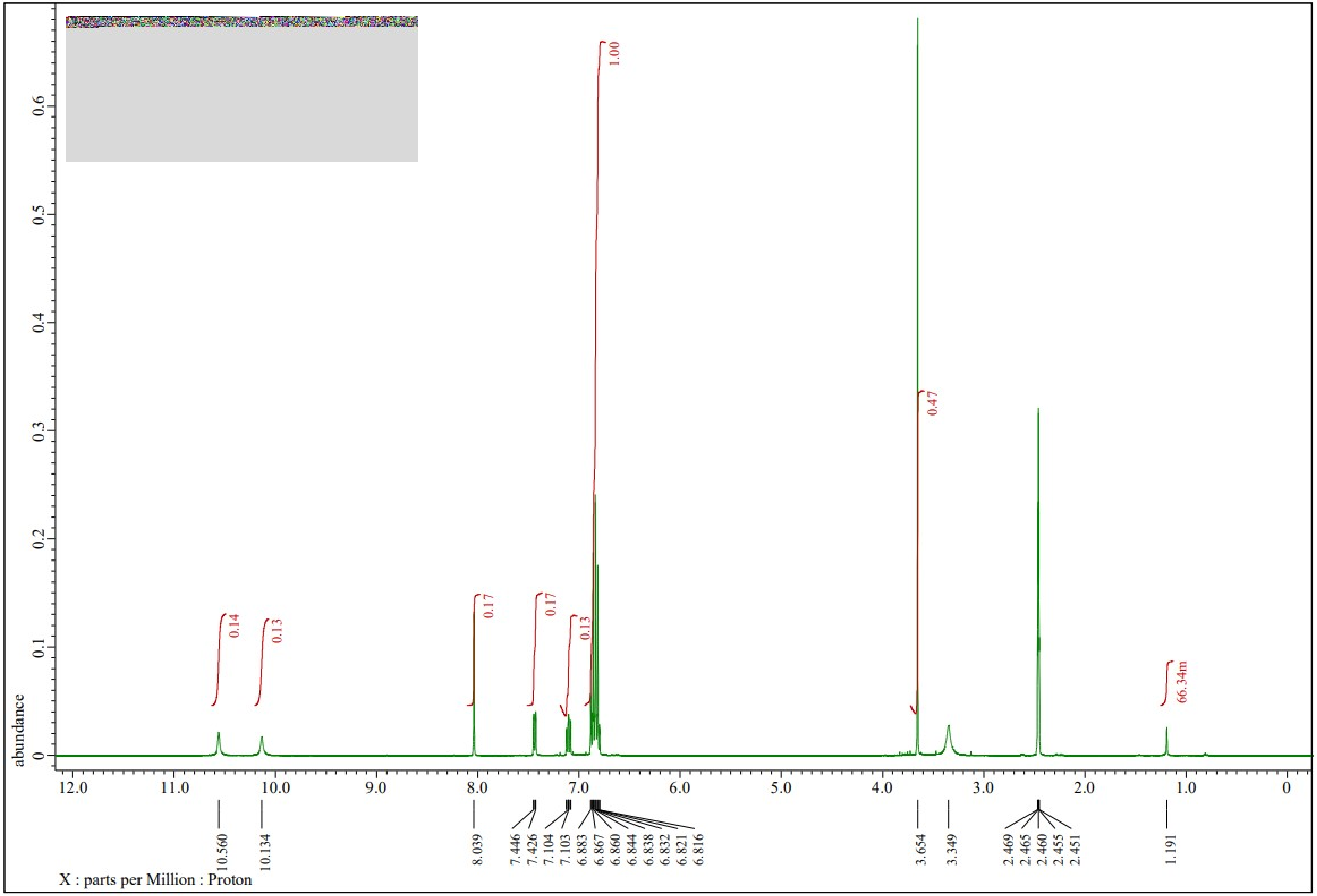

Figure S23. NMR spectrum of (*E*)-2-((2-(4-methoxyphenyl)hydrazineylidene)methyl)phenol (**3**).

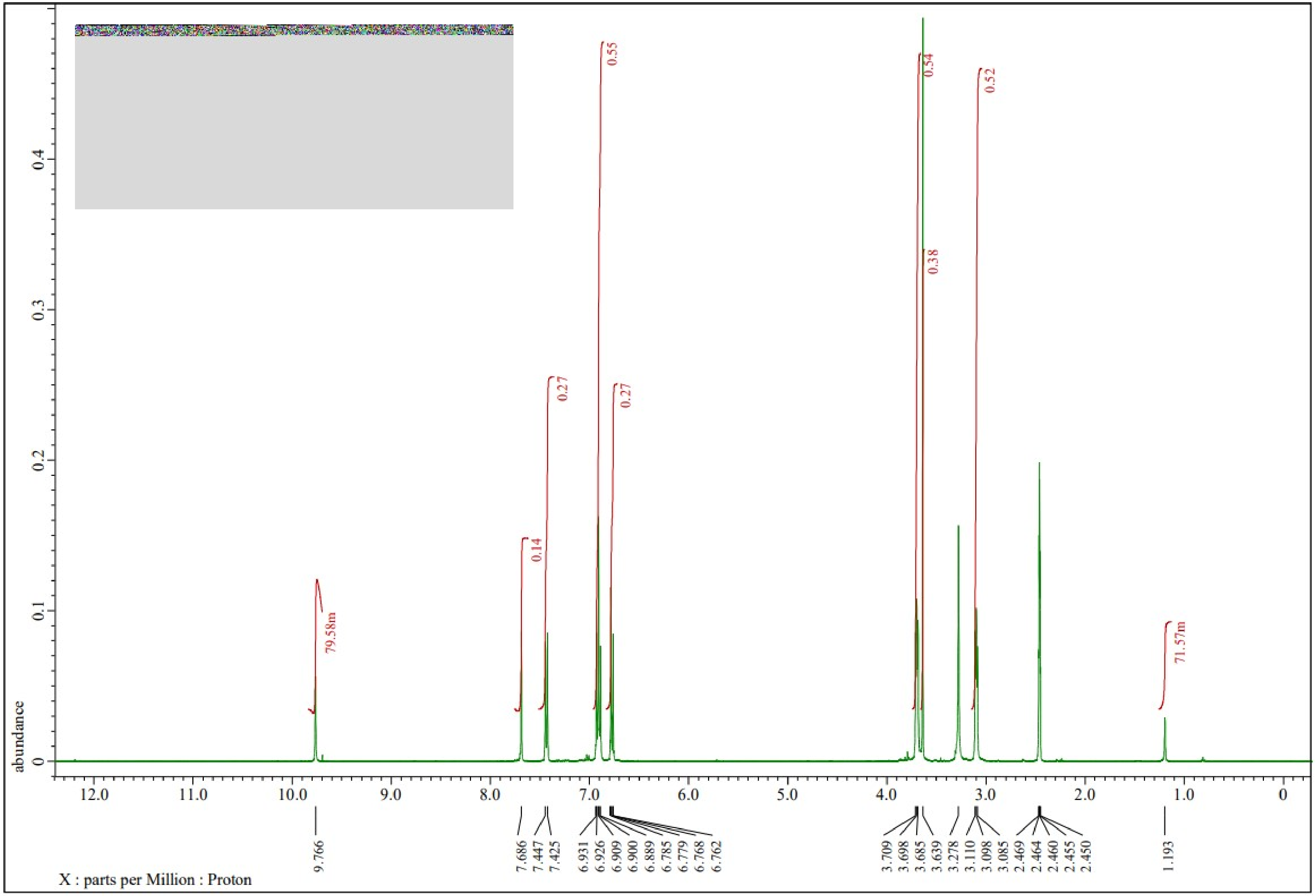

Figure S24. NMR spectrum of (*E*)-4-(4-((2-(4-methoxyphenyl)hydrazineylidene)methyl)phenyl)morpholine (**4**).

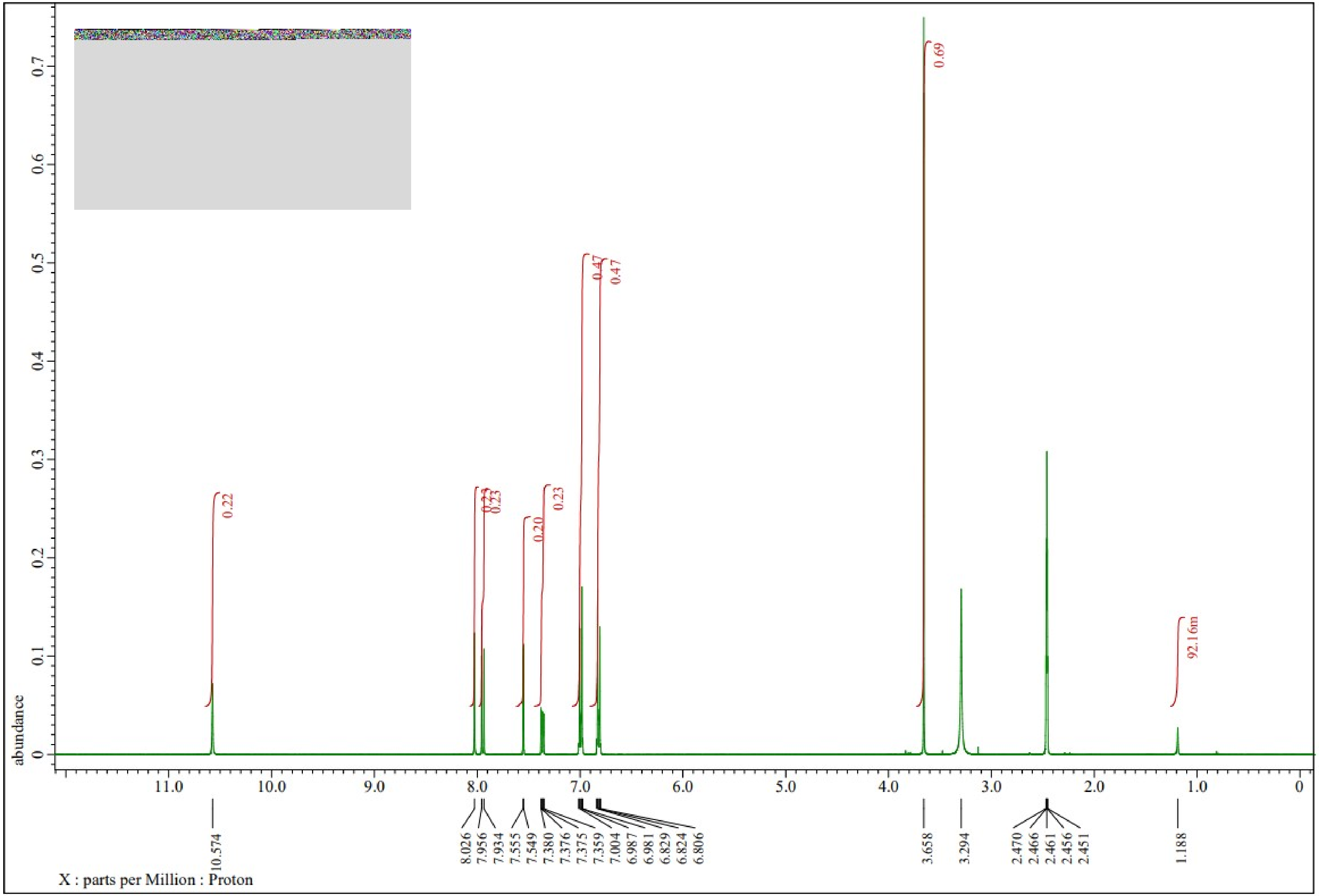

Figure S25. NMR spectrum of (*E*)-1-(2,4-dichlorobenzylidene)-2-(4-methoxyphenyl)hydrazine (**5**).

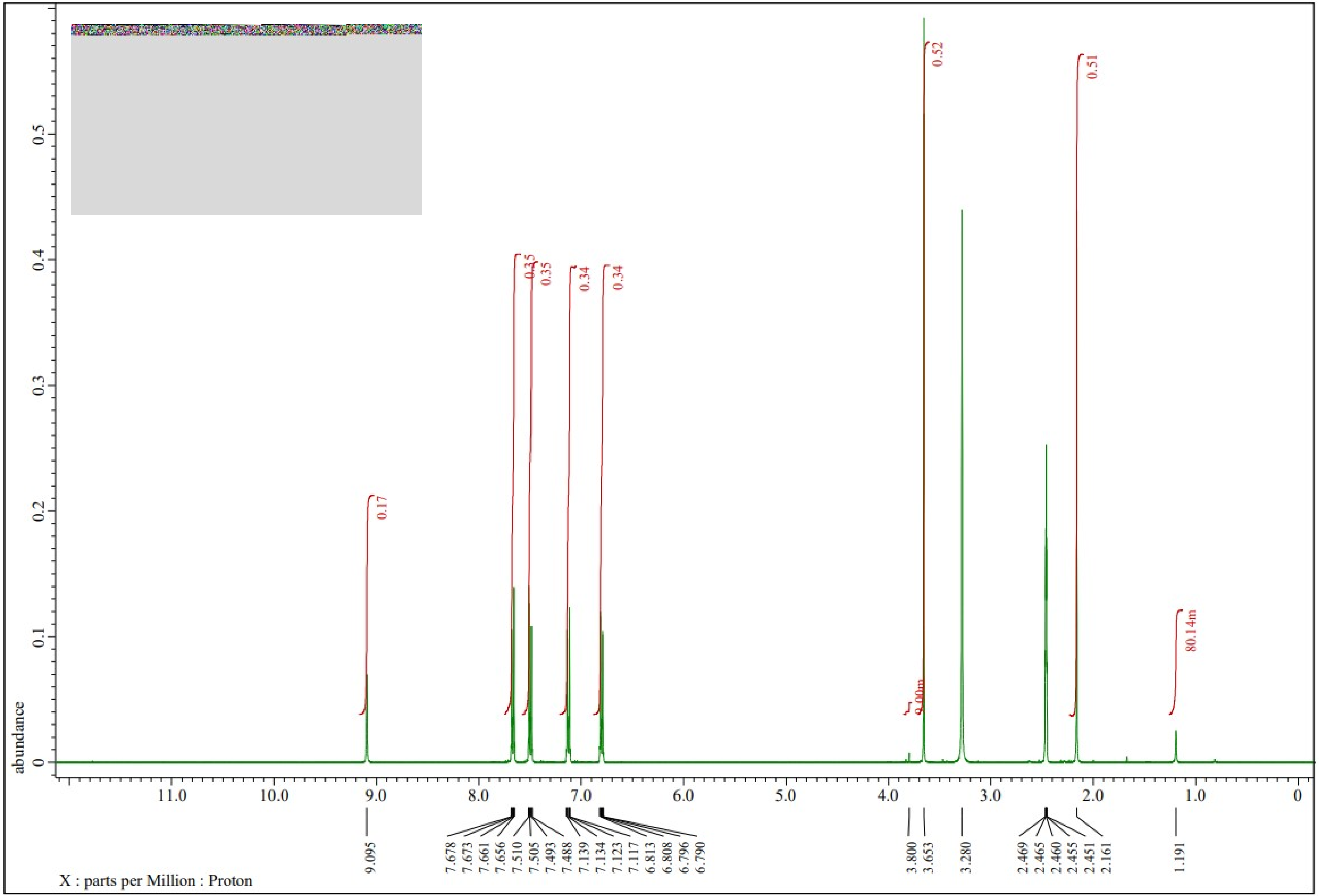

Figure S26. NMR spectrum of (*E*)-1-(1-(4-bromophenyl)ethylidene)-2-(4-methoxyphenyl)hydrazine (**6**).

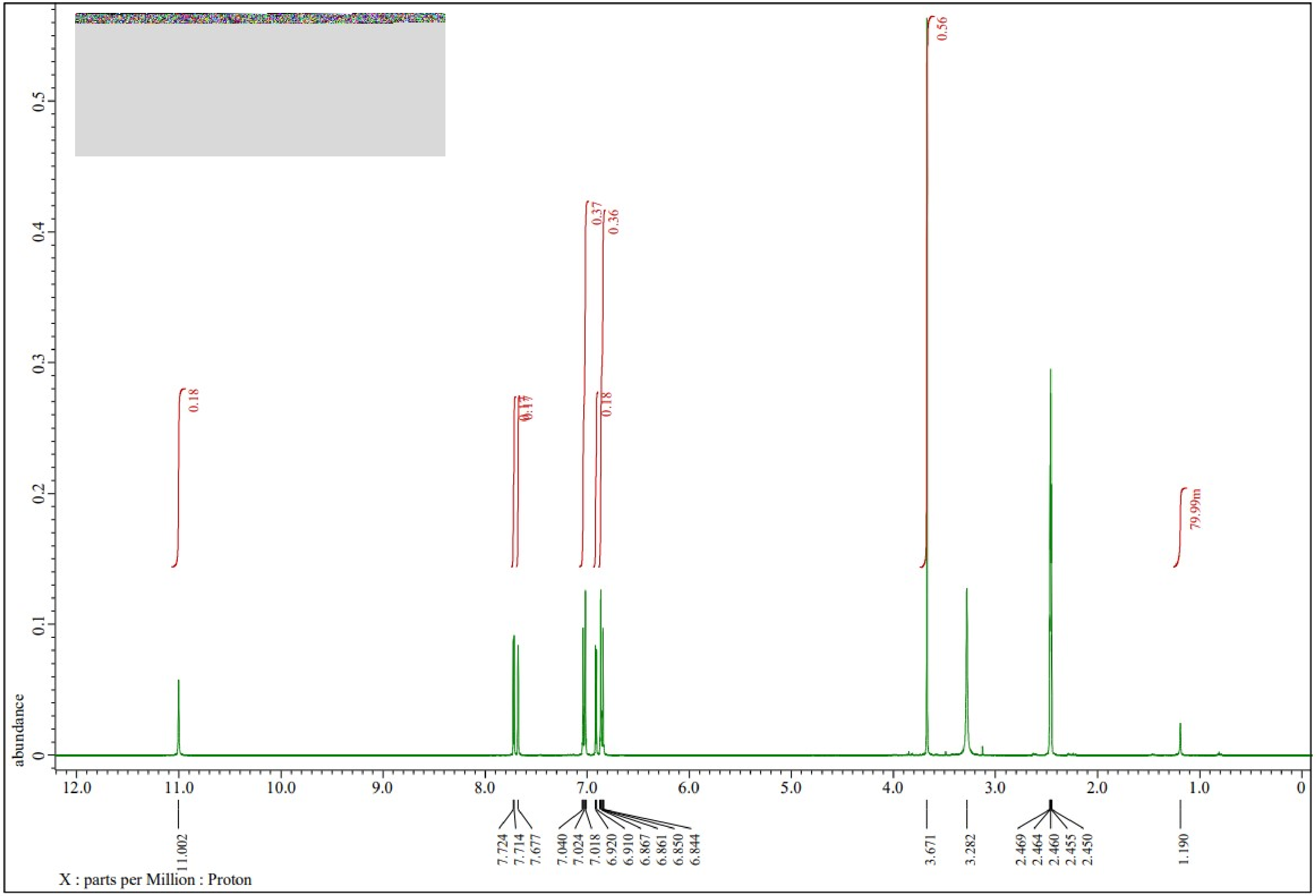

Figure S27. NMR spectrum of (*E*)-1-(4-methoxyphenyl)-2-((5-nitrofuran-2-yl)methylene)hydrazine (**7**).

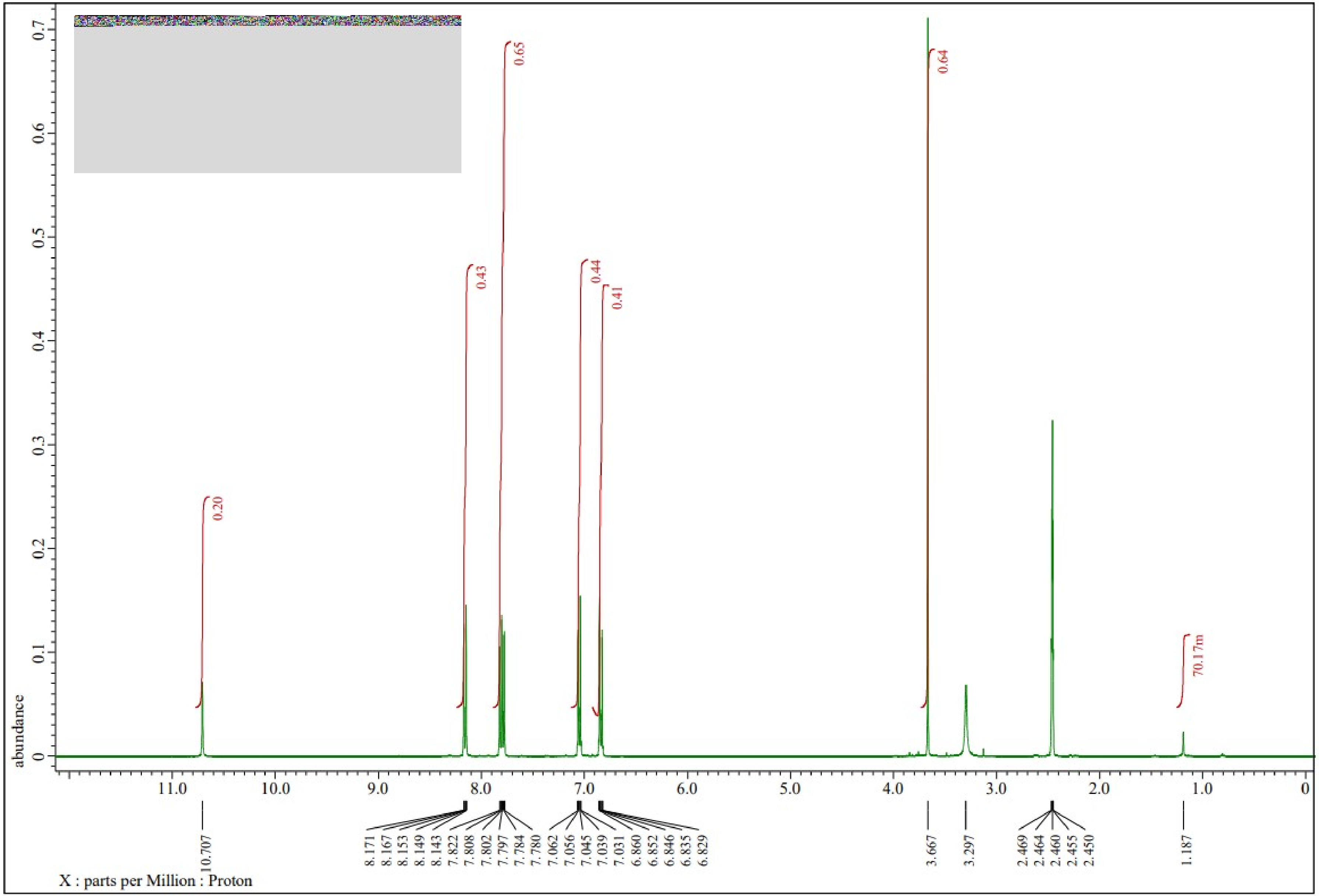

Figure S28. NMR spectrum of (*E*)-1-(4-methoxyphenyl)-2-(4-nitrobenzylidene)hydrazine (**8**).

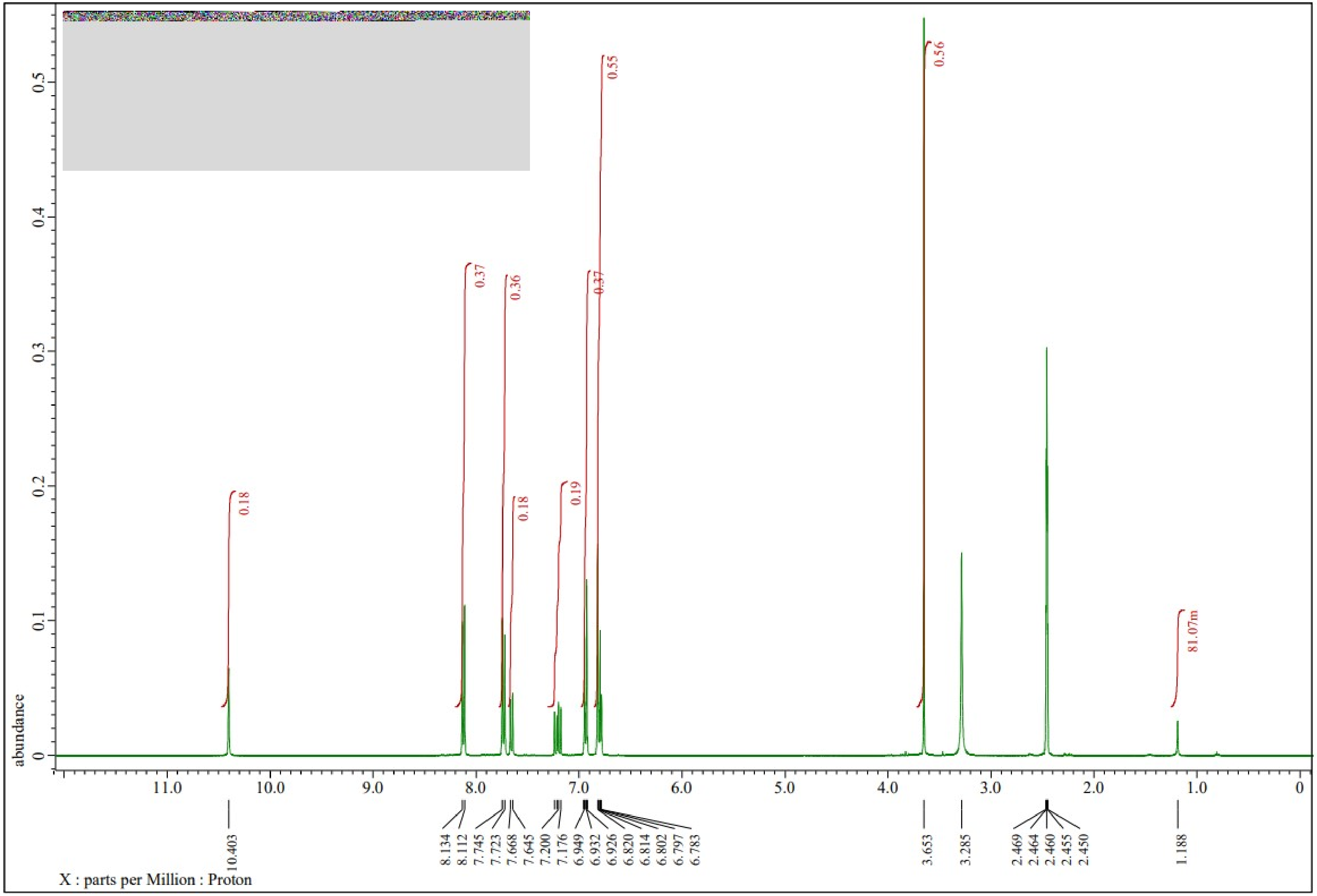

Figure S29. NMR spectrum of (*E*)-1-(4-methoxyphenyl)-2-(3-(4-nitrophenyl)propylidene)hydrazine (**9**).

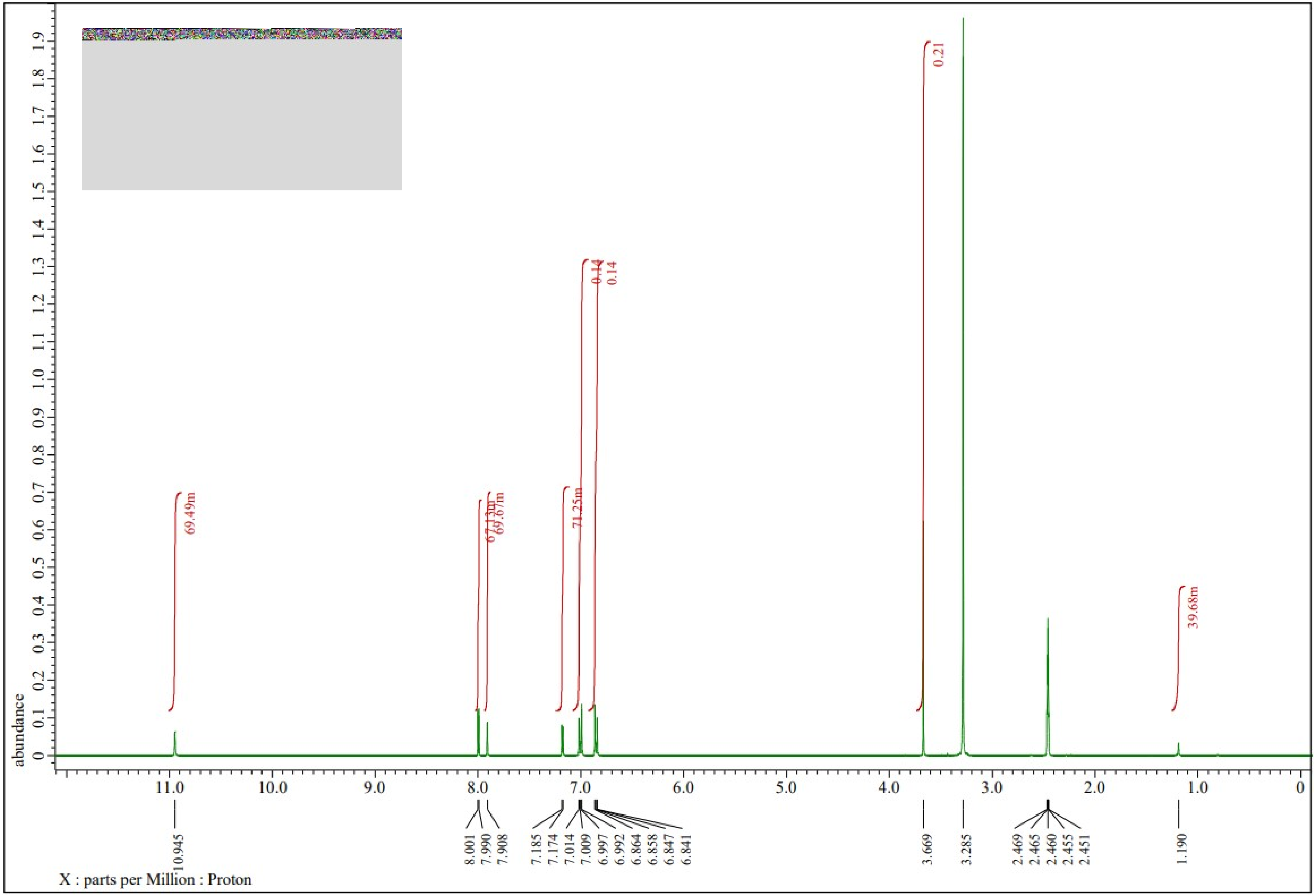

Figure S30. NMR spectrum of (*E*)-1-(4-methoxyphenyl)-2-((5-nitrothiophen-2-yl)methylene)hydrazine (**10**).

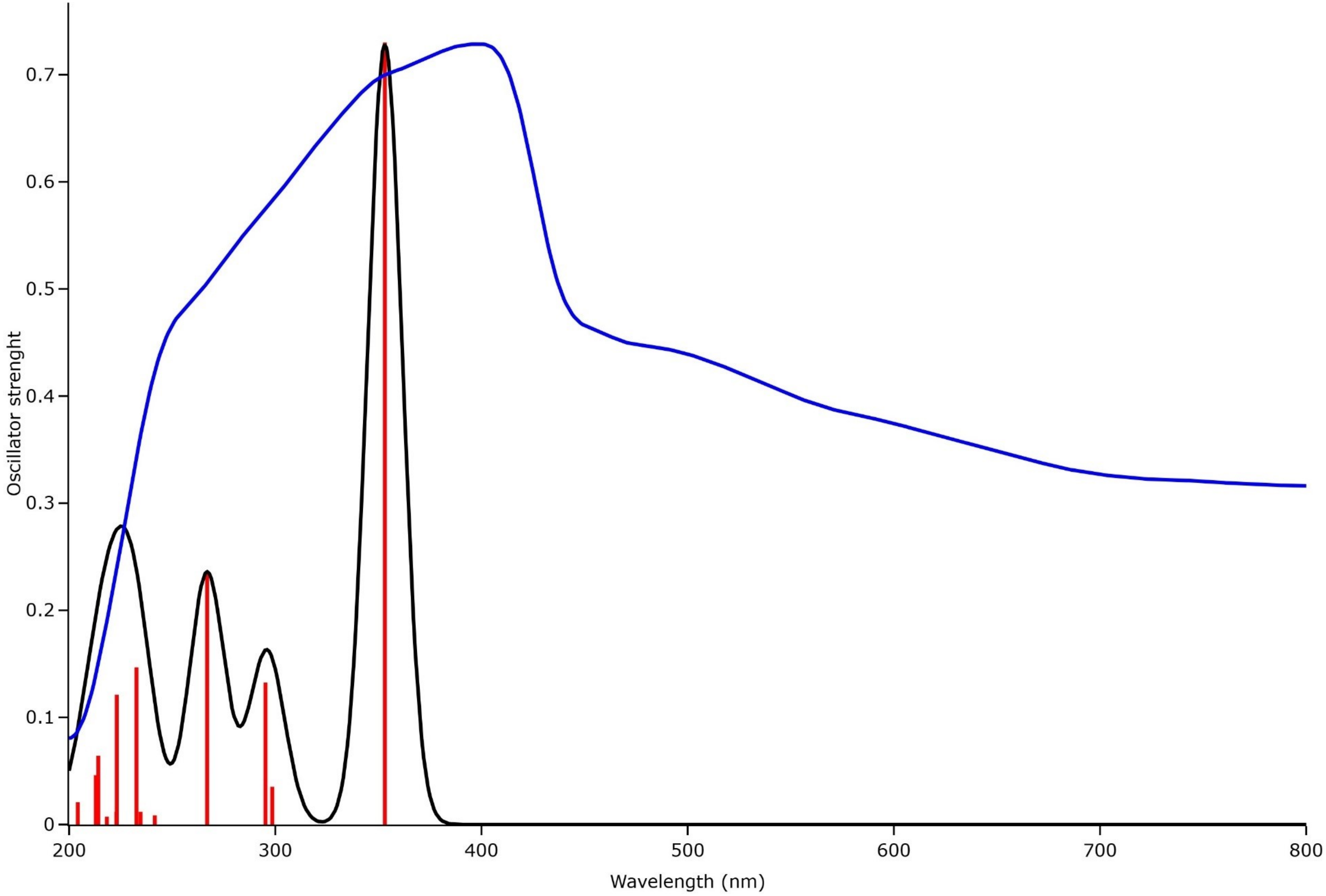

Figure S31. Experimental (blue) and calculated (black) UV-Vis spectrum of **1**. Oscillator strengths are represented as vertical navy-red lines.

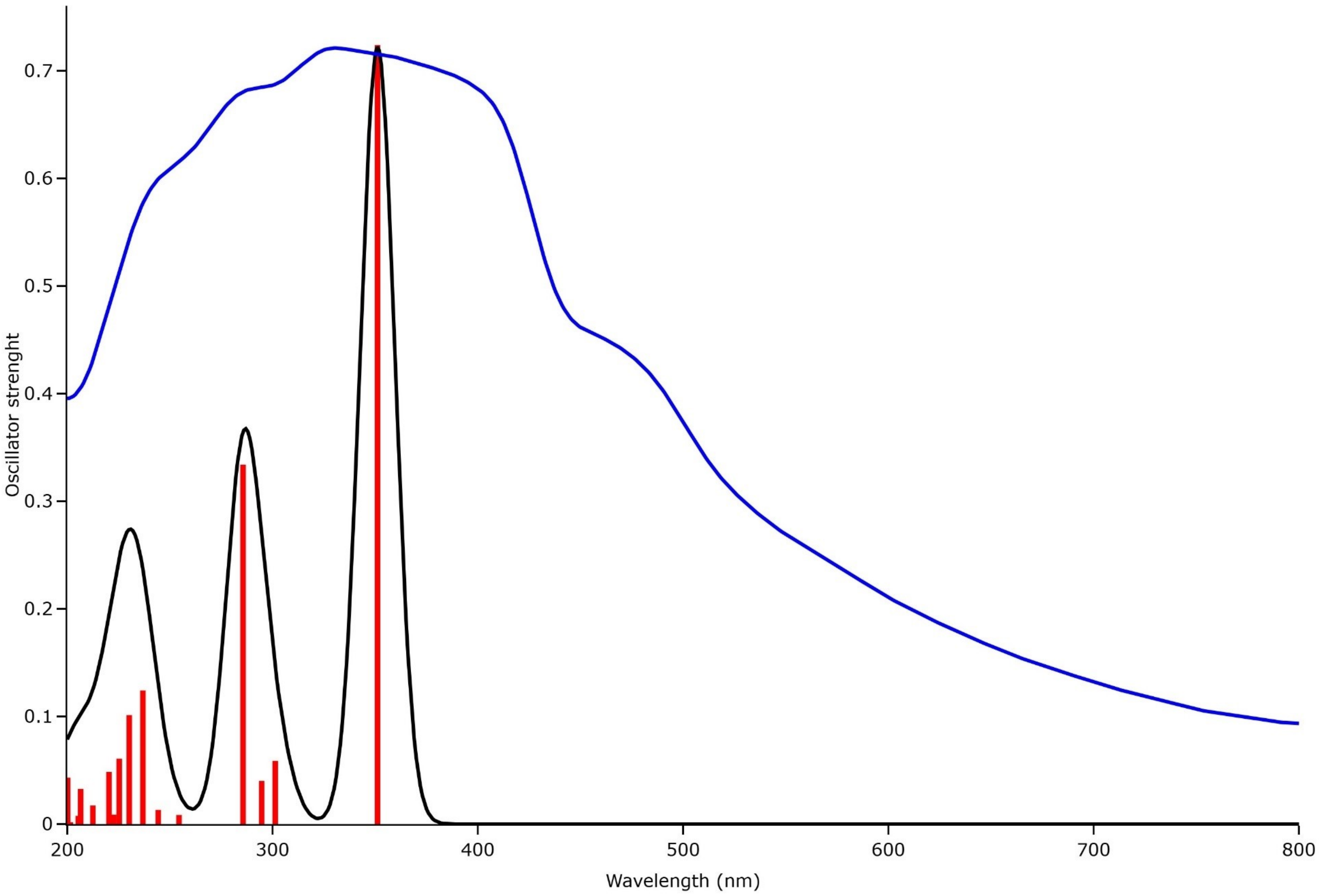

Figure S32. Experimental (blue) and calculated (black) UV-Vis spectrum of **2**. Oscillator strengths are represented as vertical navy-red lines.

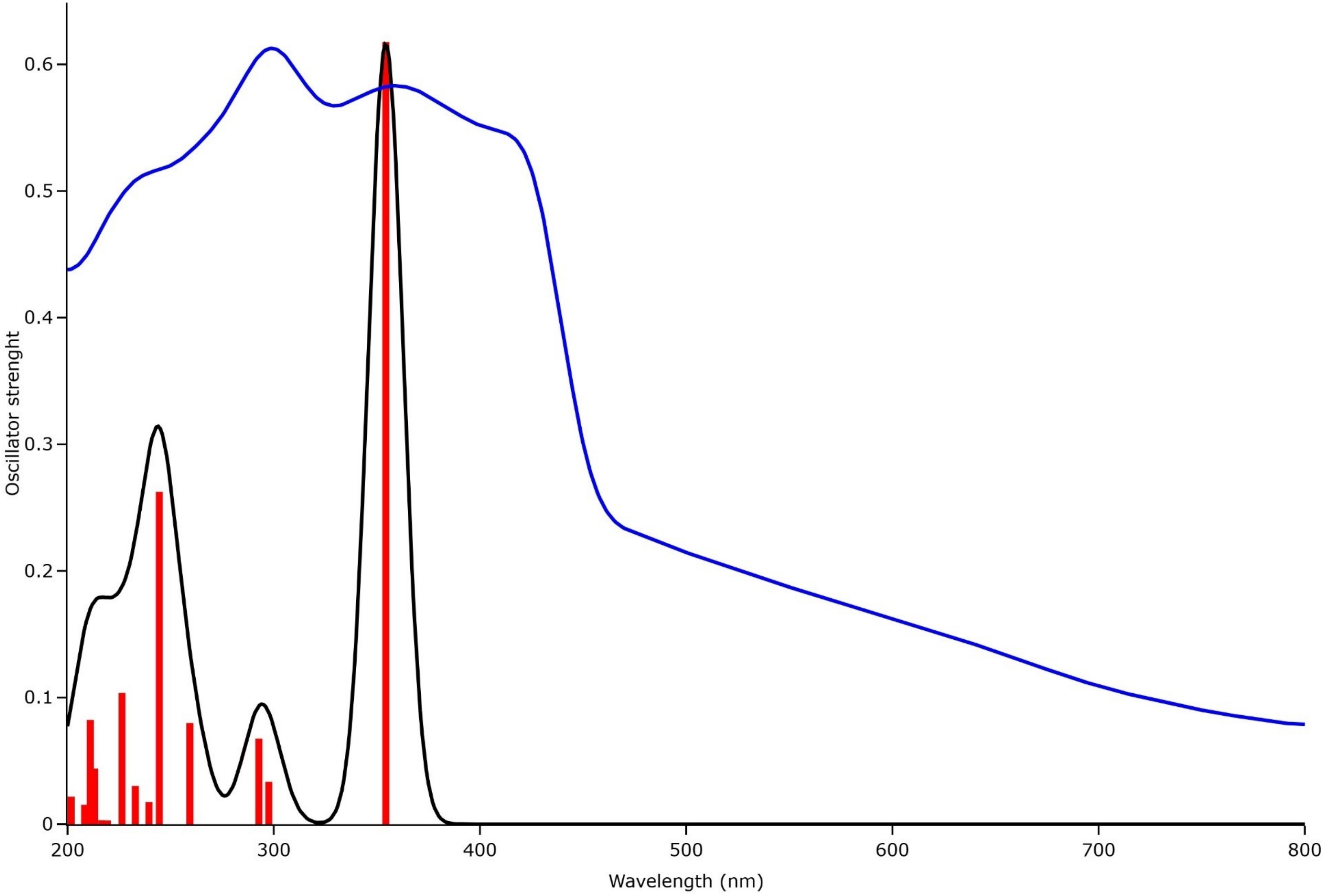

Figure S33. Experimental (blue) and calculated (black) UV-Vis spectrum of **3**. Oscillator strengths are represented as vertical navy-red lines.

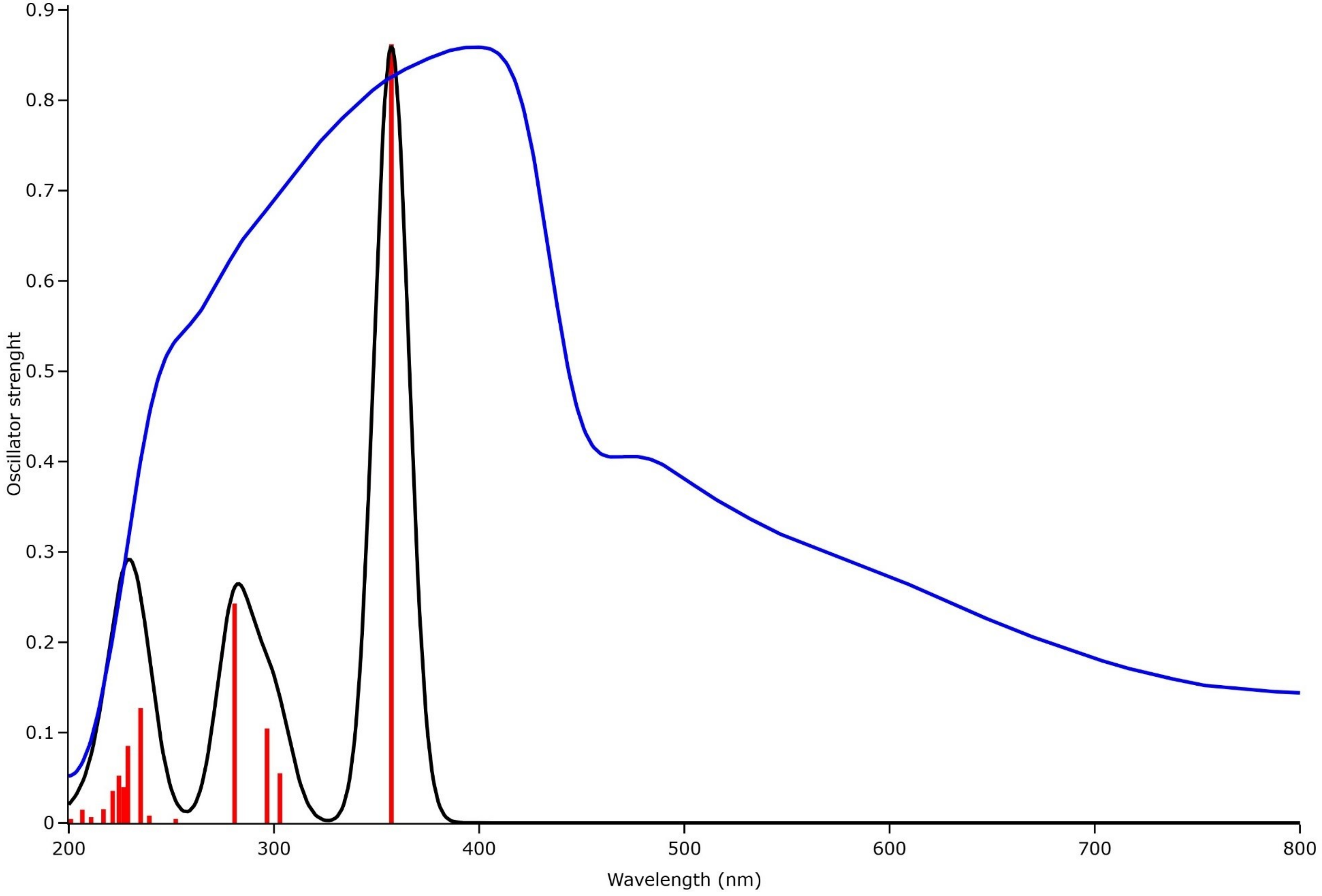

Figure S34. Experimental (blue) and calculated (black) UV-Vis spectrum of **4**. Oscillator strengths are represented as vertical navy-red lines.

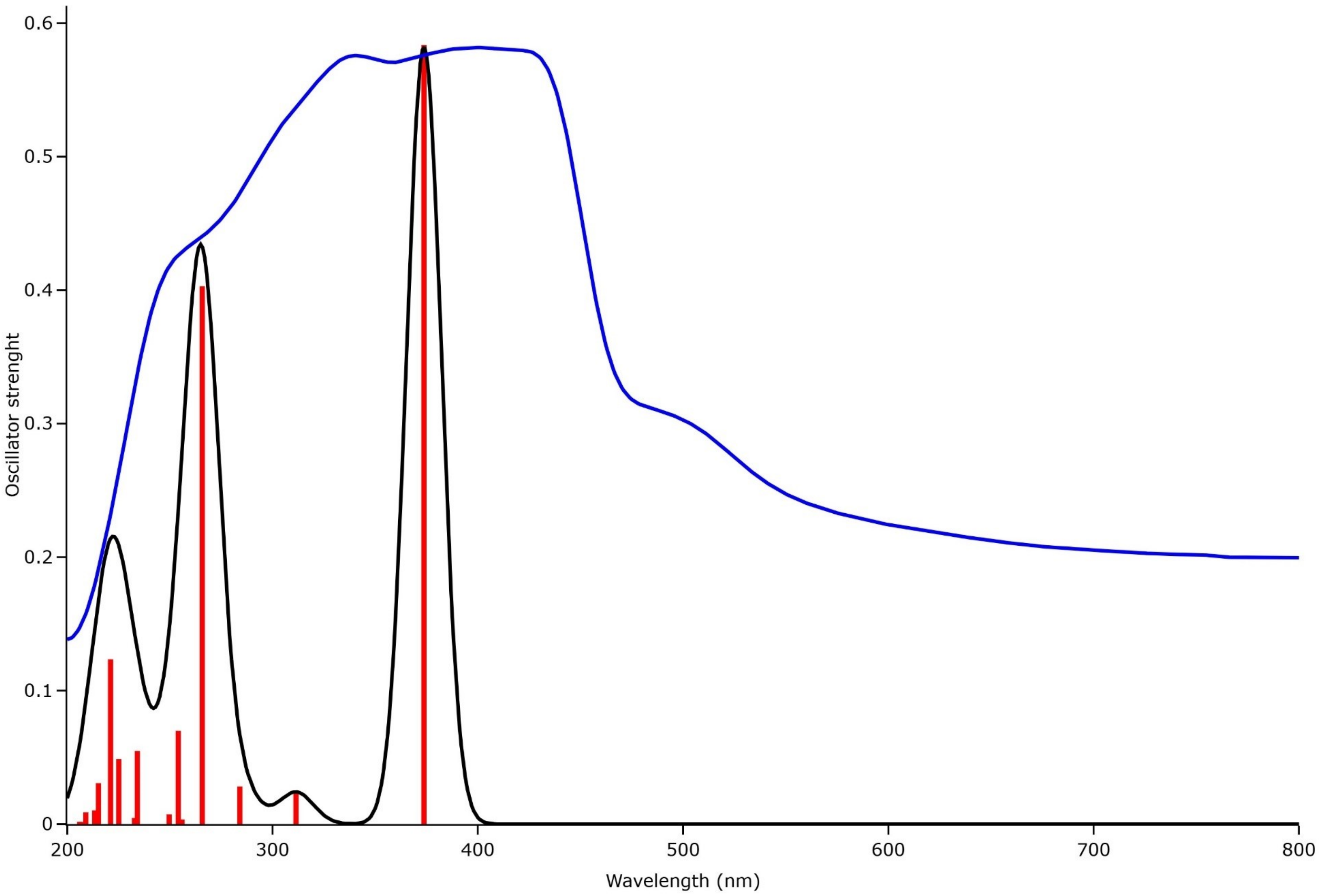

Figure S35. Experimental (blue) and calculated (black) UV-Vis spectrum of **5**. Oscillator strengths are represented as vertical navy-red lines.

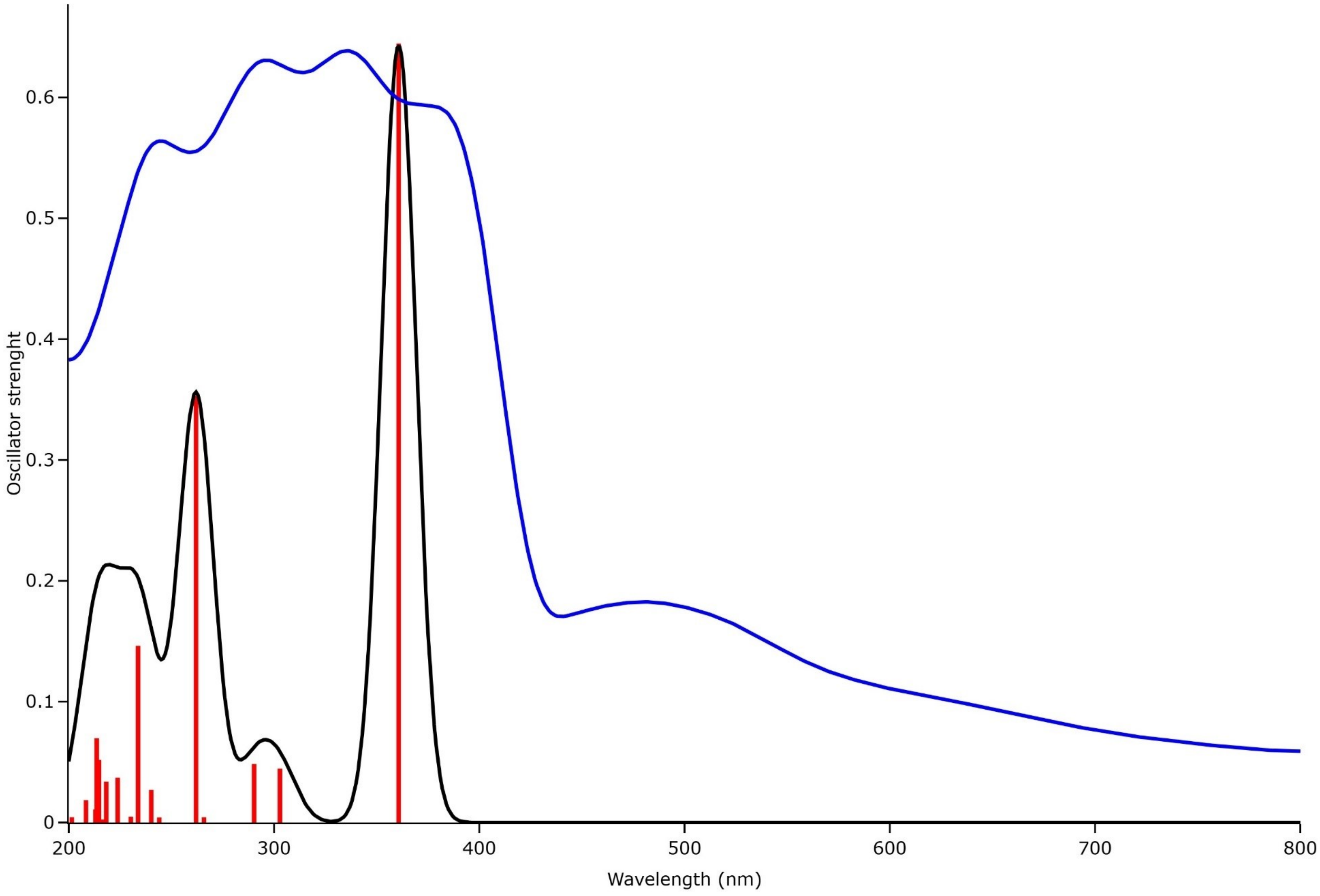

Figure S36. Experimental (blue) and calculated (black) UV-Vis spectrum of **6**. Oscillator strengths are represented as vertical navy-red lines.

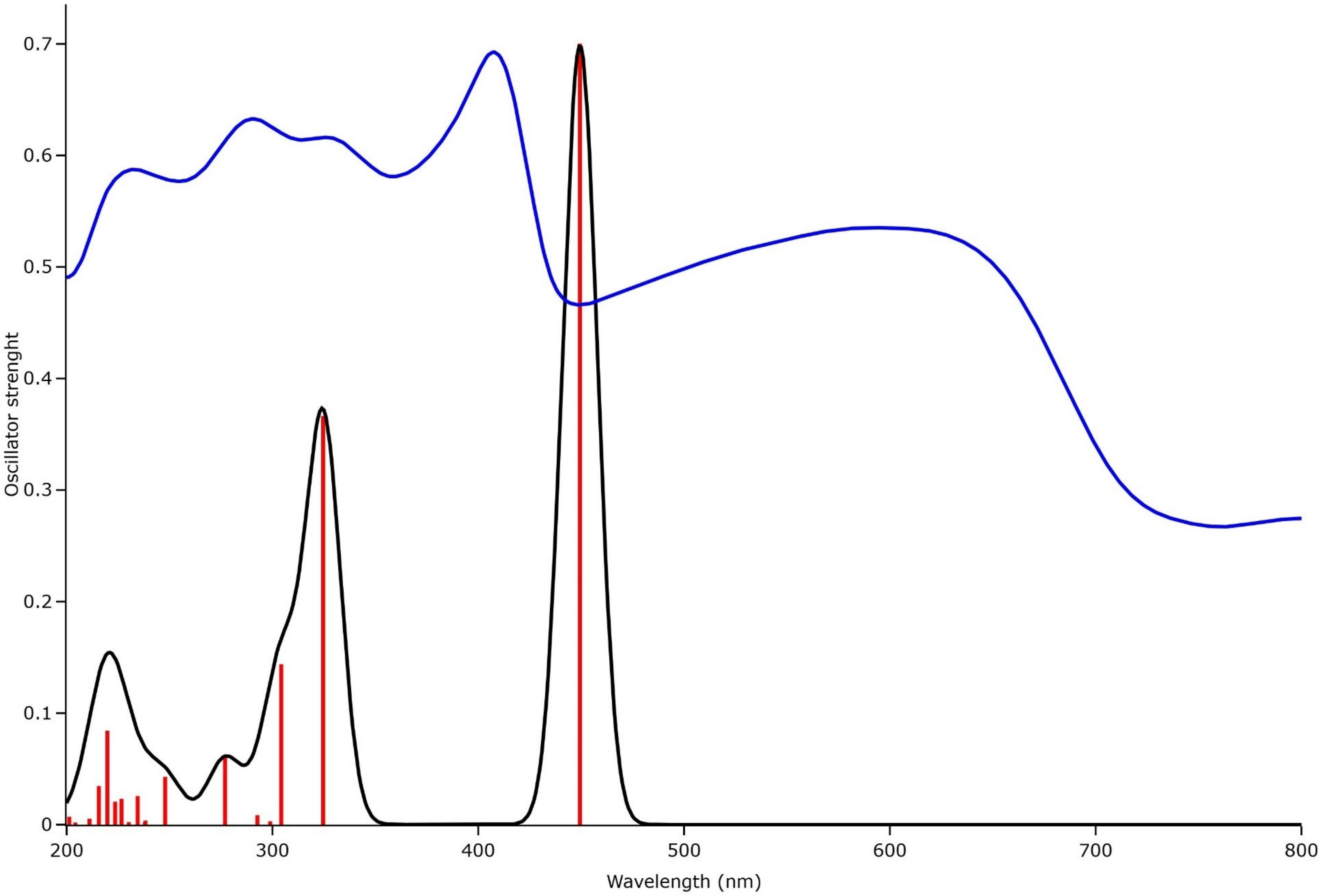

Figure S37. Experimental (blue) and calculated (black) UV-Vis spectrum of **7**. Oscillator strengths are represented as vertical navy-red lines.

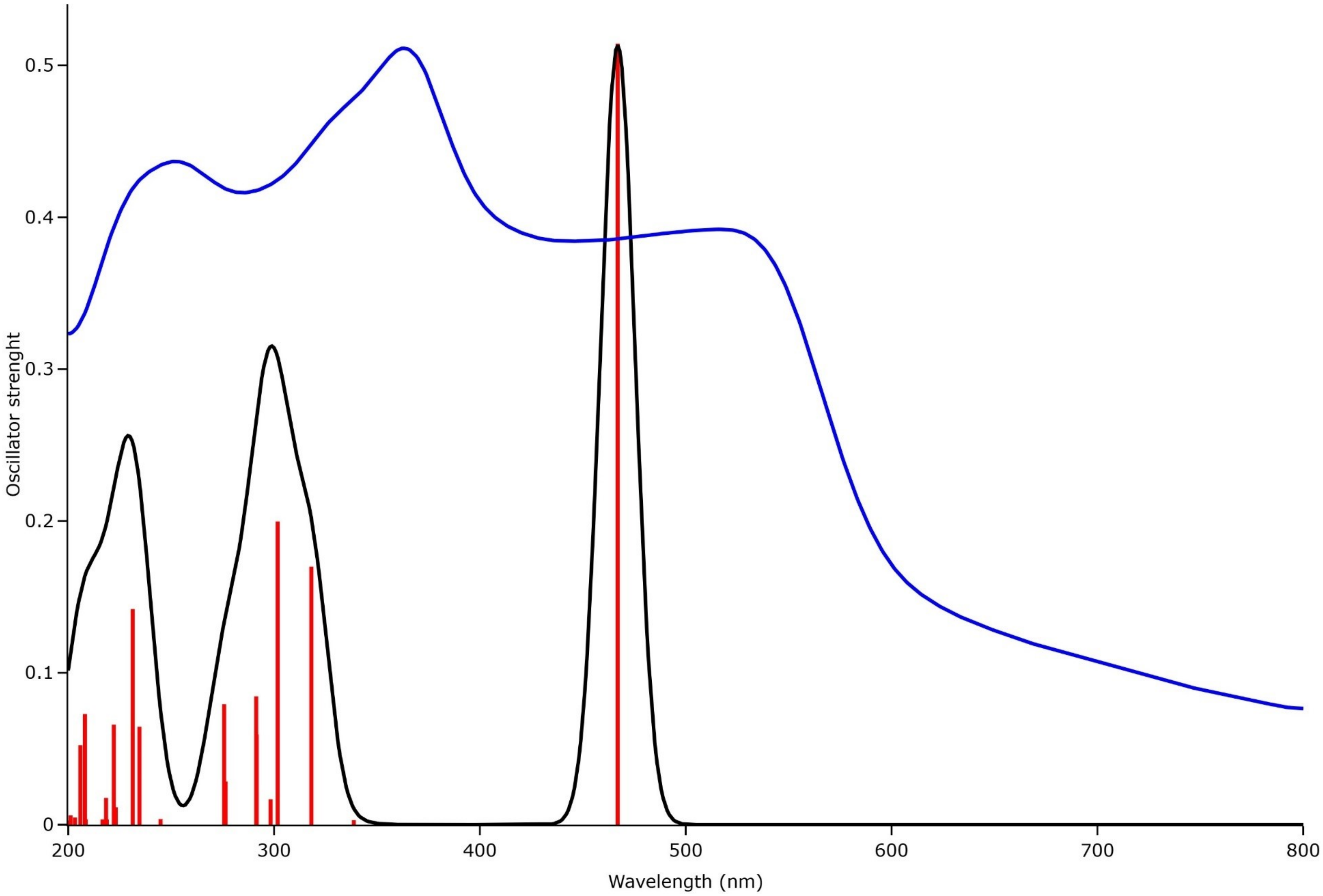

Figure S38. Experimental (blue) and calculated (black) UV-Vis spectrum of **8**. Oscillator strengths are represented as vertical navy-red lines.

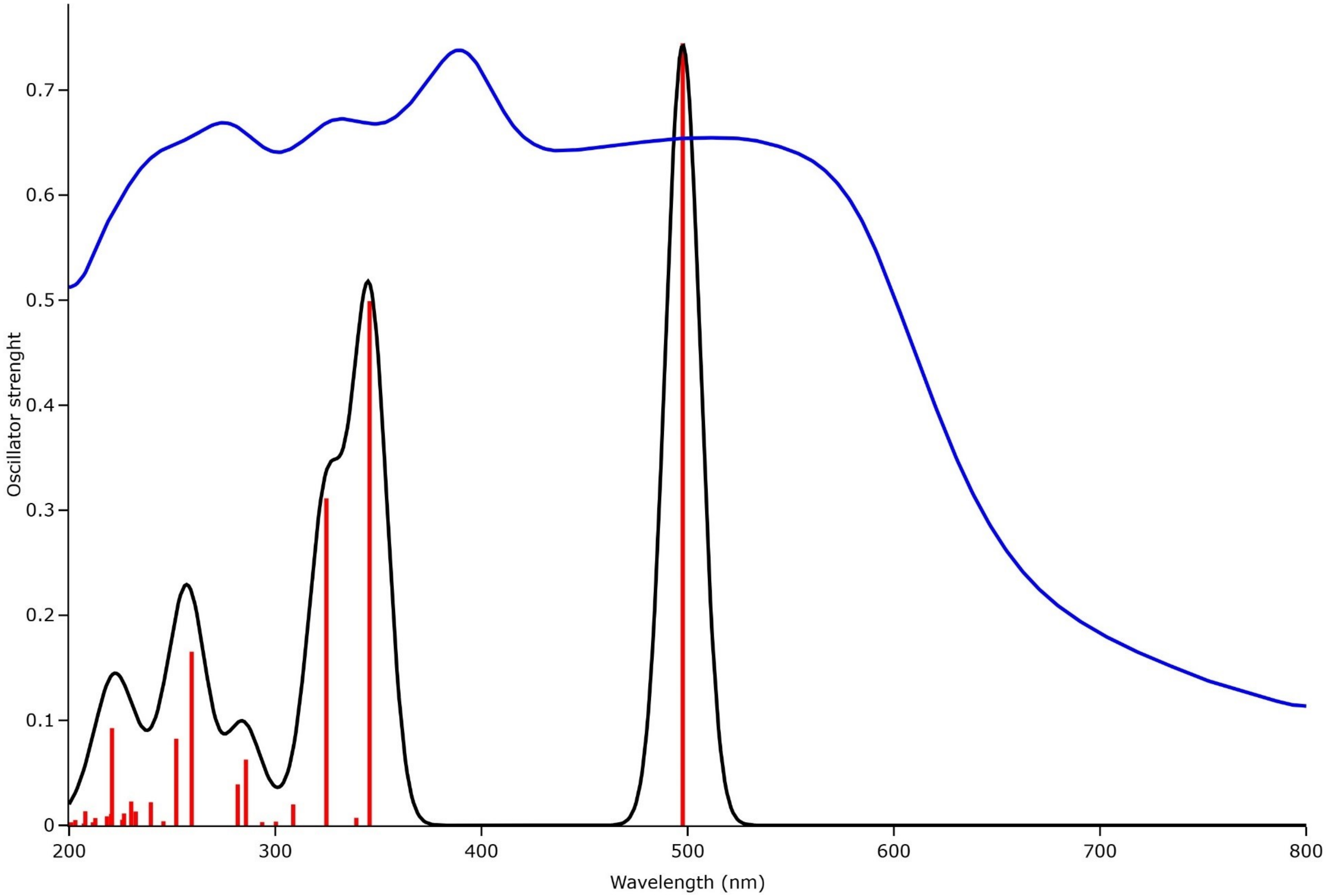

Figure S39. Experimental (blue) and calculated (black) UV-Vis spectrum of **9**. Oscillator strengths are represented as vertical navy-red lines.

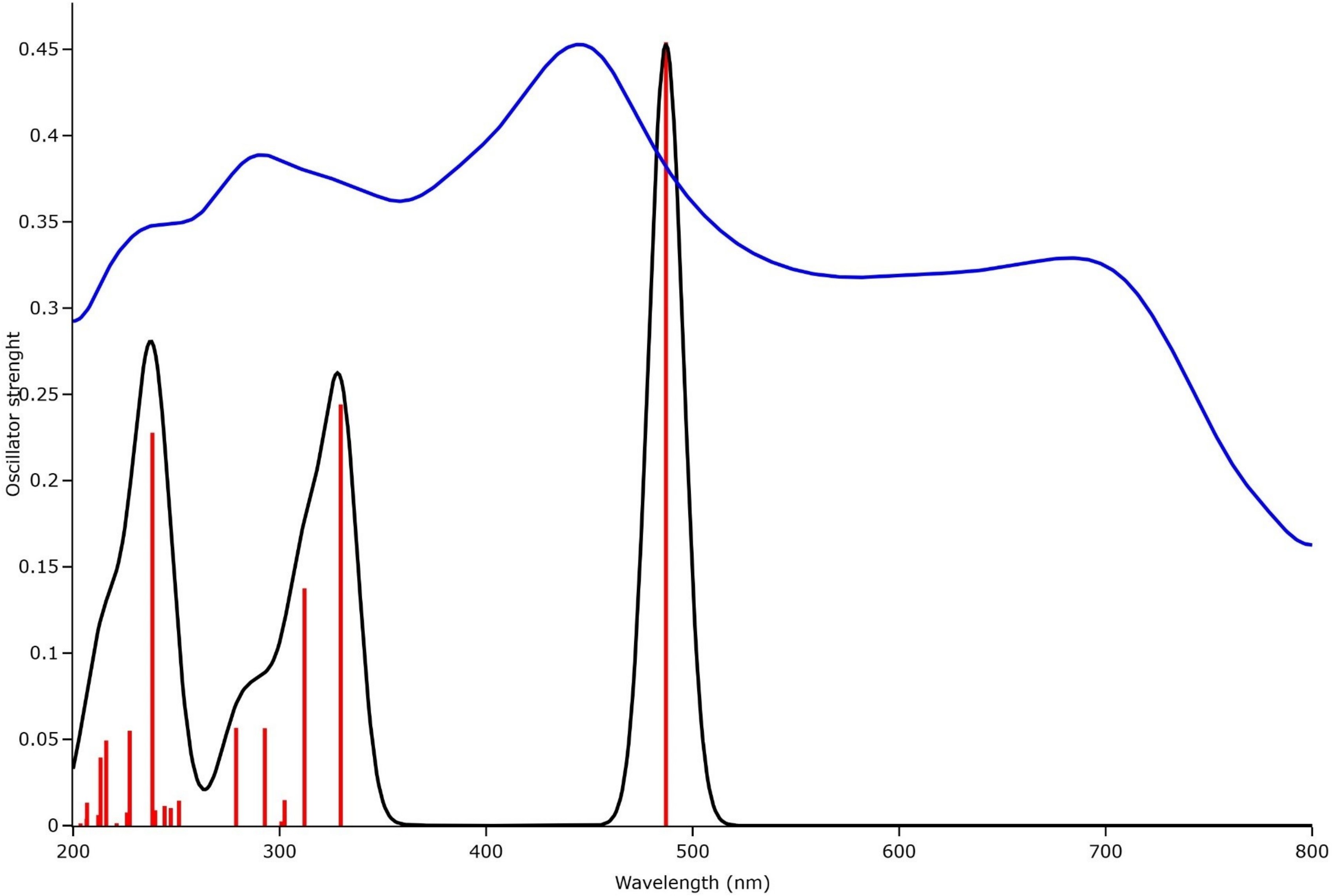

Figure S40. Experimental (blue) and calculated (black) UV-Vis spectrum of **10**. Oscillator strengths are represented as vertical navy-red lines.

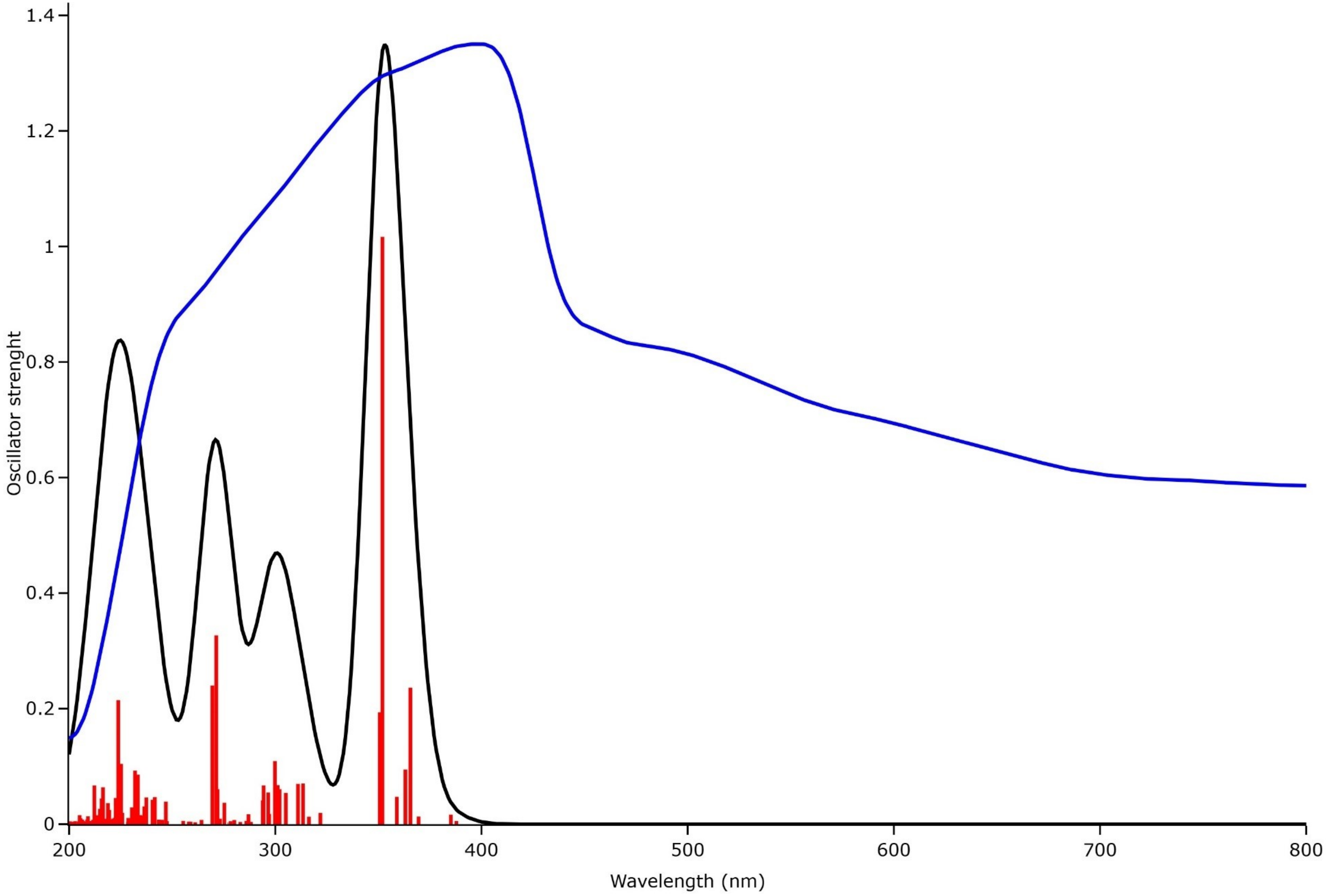

Figure S41. Experimental (blue) and calculated for 3 molecules (black) UV-Vis spectrum of **1**. Oscillator strengths are represented as vertical navy-red lines.

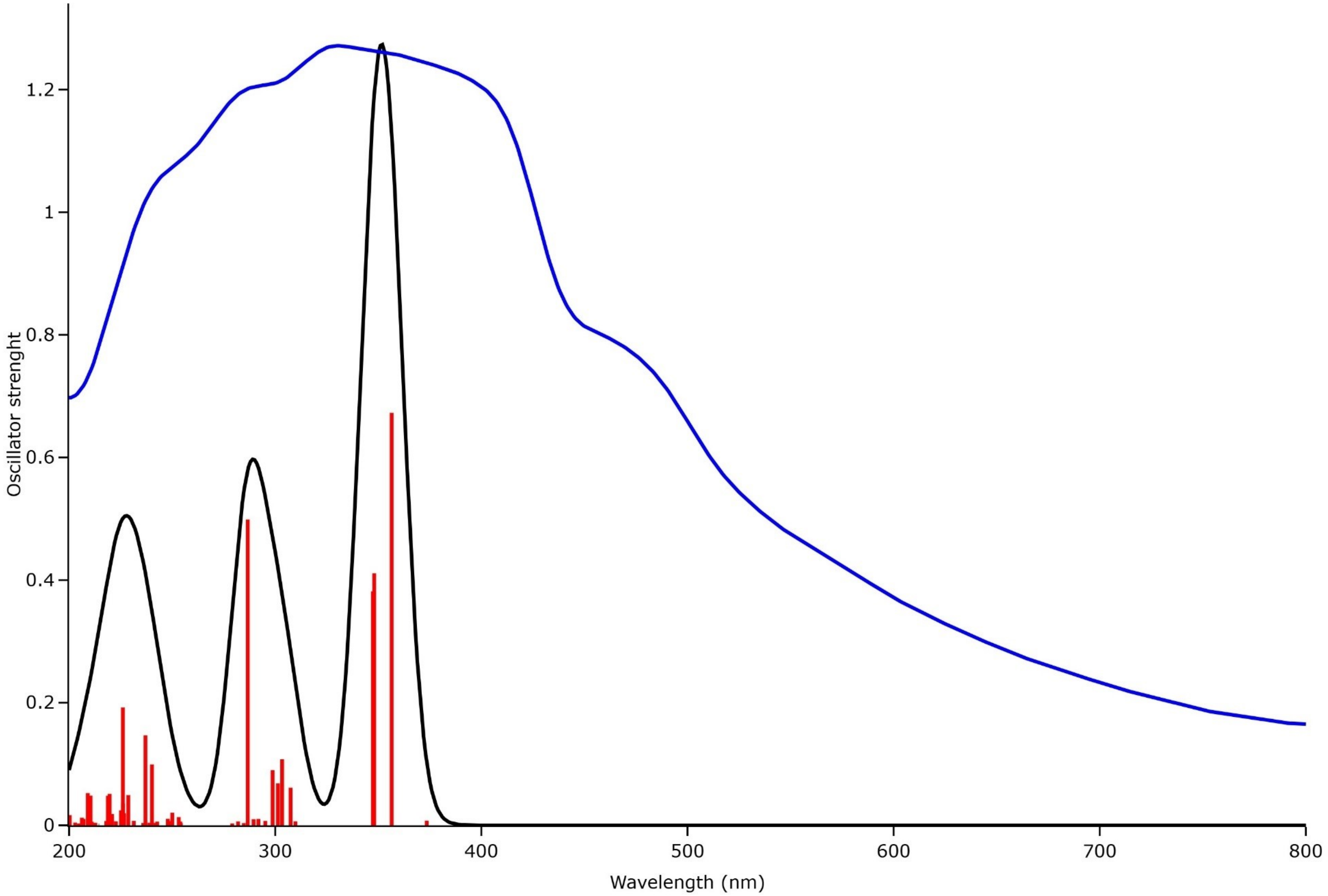

Figure S42. Experimental (blue) and calculated for 2 molecules (black) UV-Vis spectrum of **2**. Oscillator strengths are represented as vertical navy-red lines.

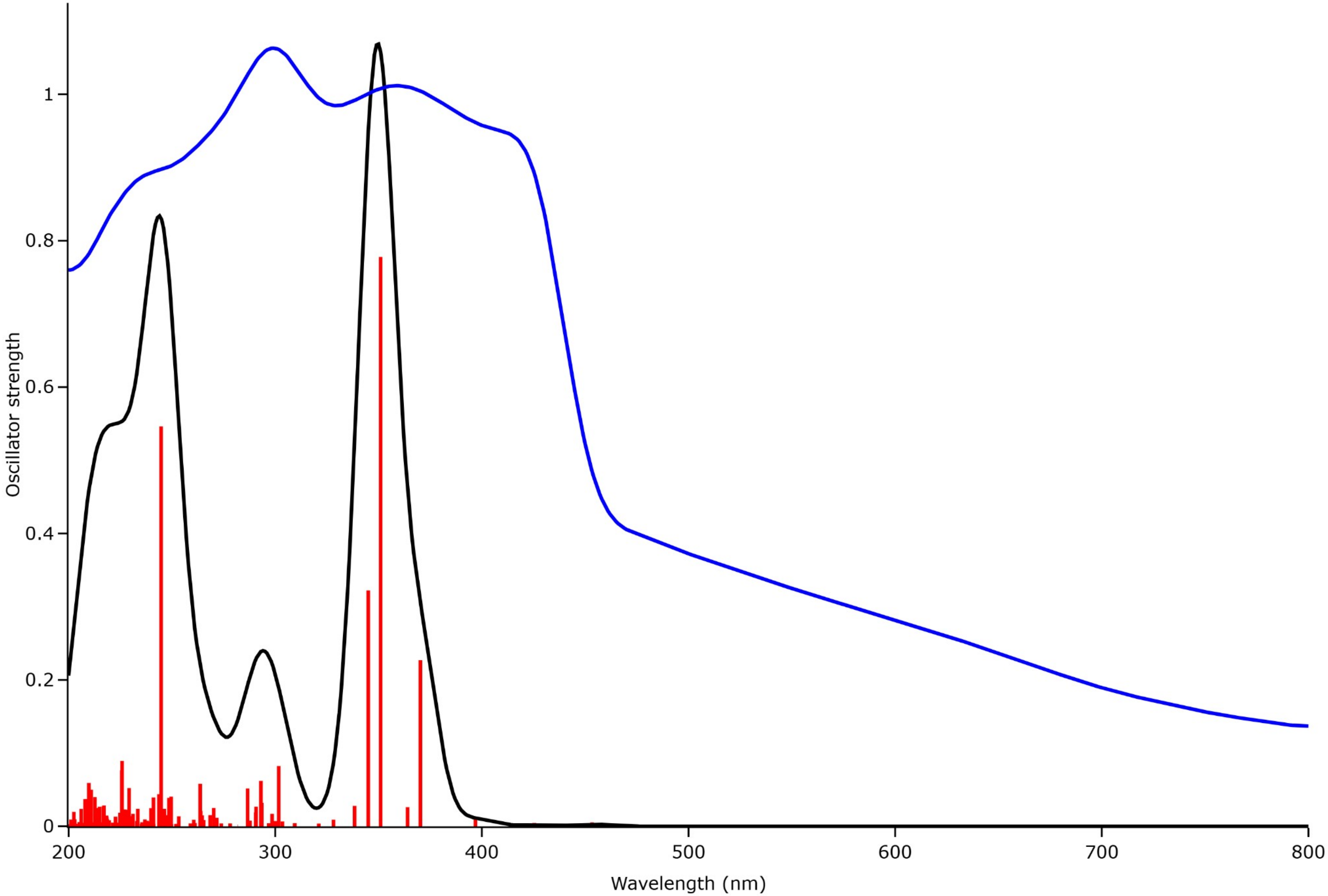

Figure S43. Experimental (blue) and calculated for 3 molecules (black) UV-Vis spectrum of **3**. Oscillator strengths are represented as vertical navy-red lines.

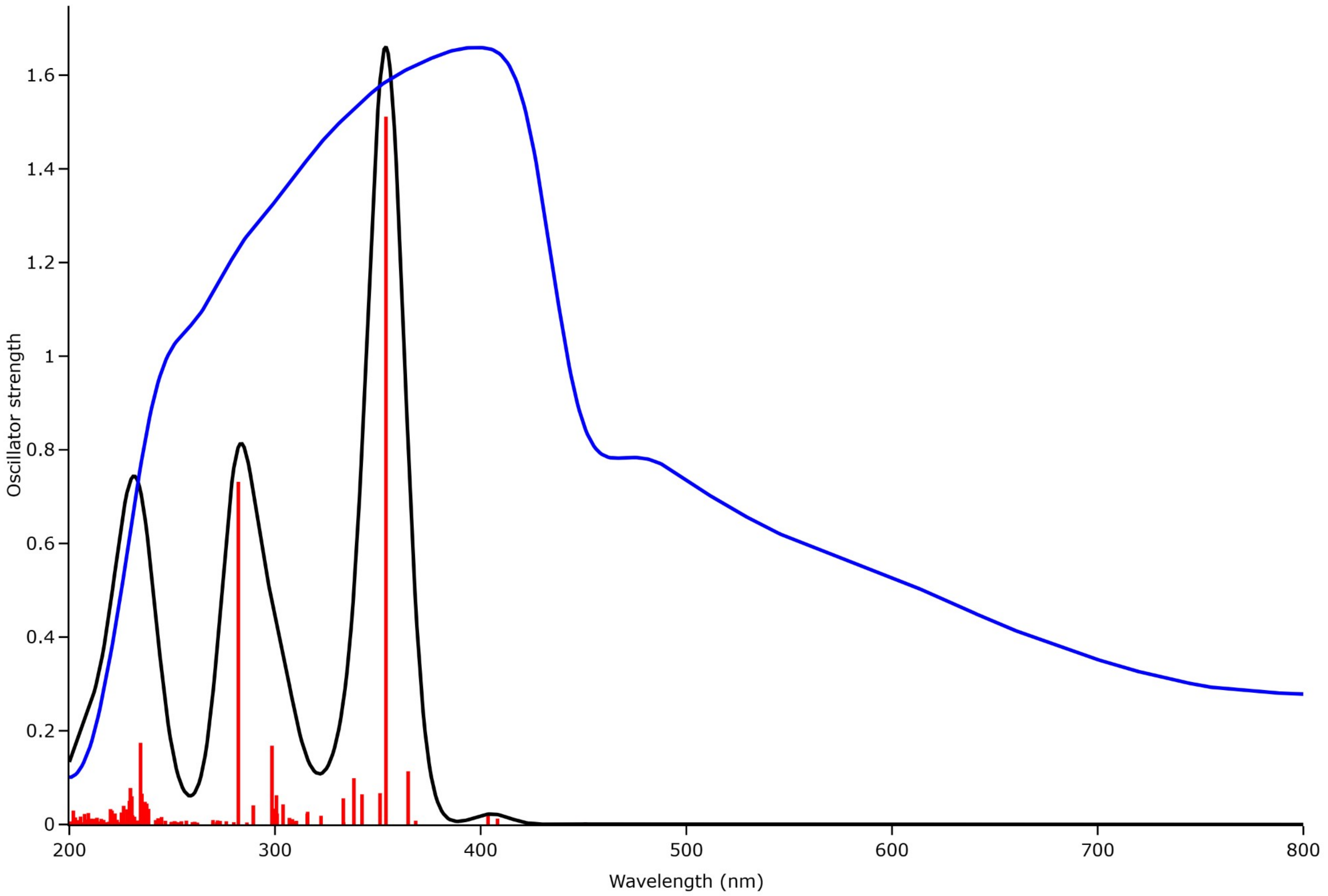

Figure S44. Experimental (blue) and calculated for 3 molecules (black) UV-Vis spectrum of **4**. Oscillator strengths are represented as vertical navy-red lines.

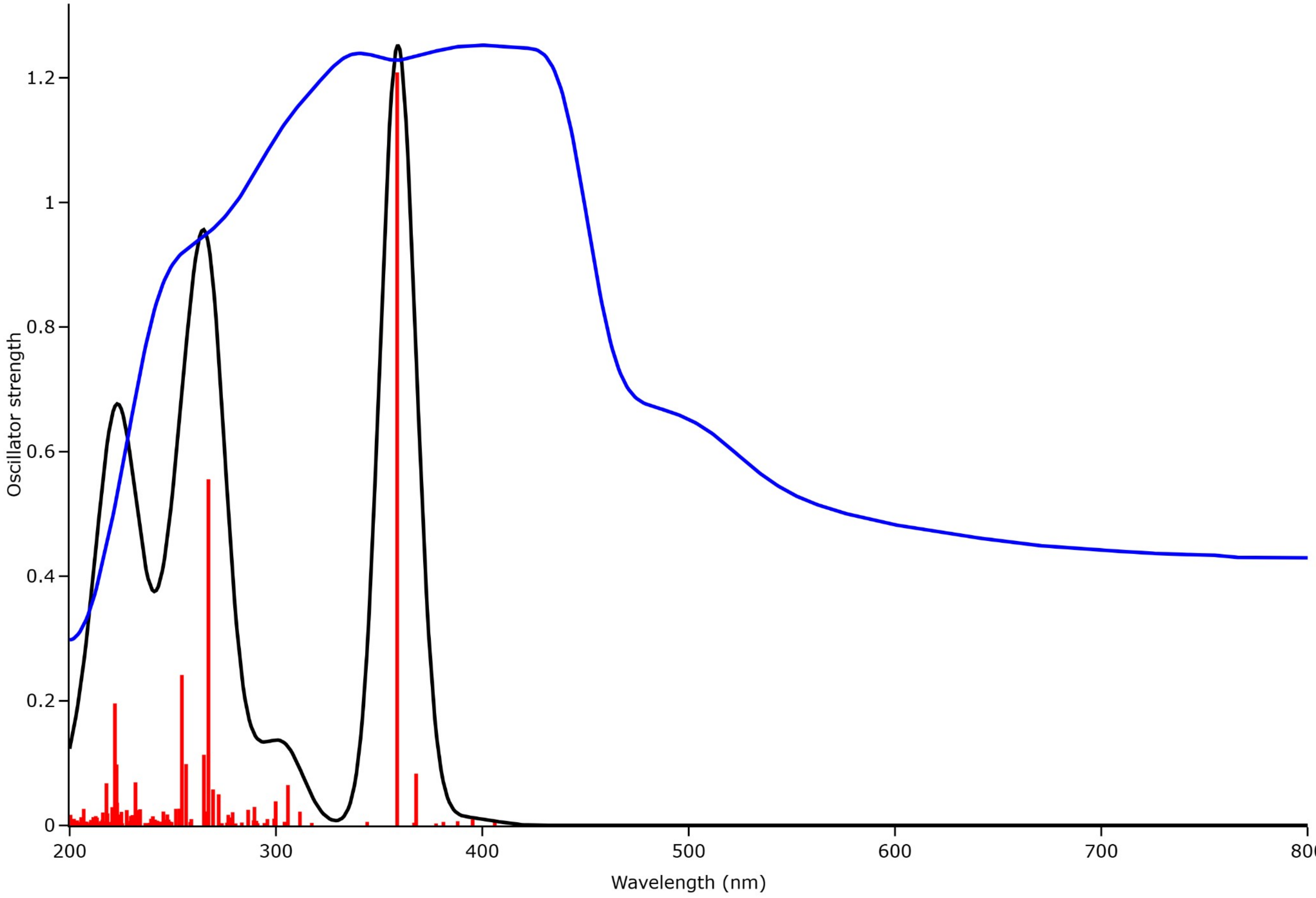

Figure S45. Experimental (blue) and calculated for 3 molecules (black) UV-Vis spectrum of **5**. Oscillator strengths are represented as vertical navy-red lines.

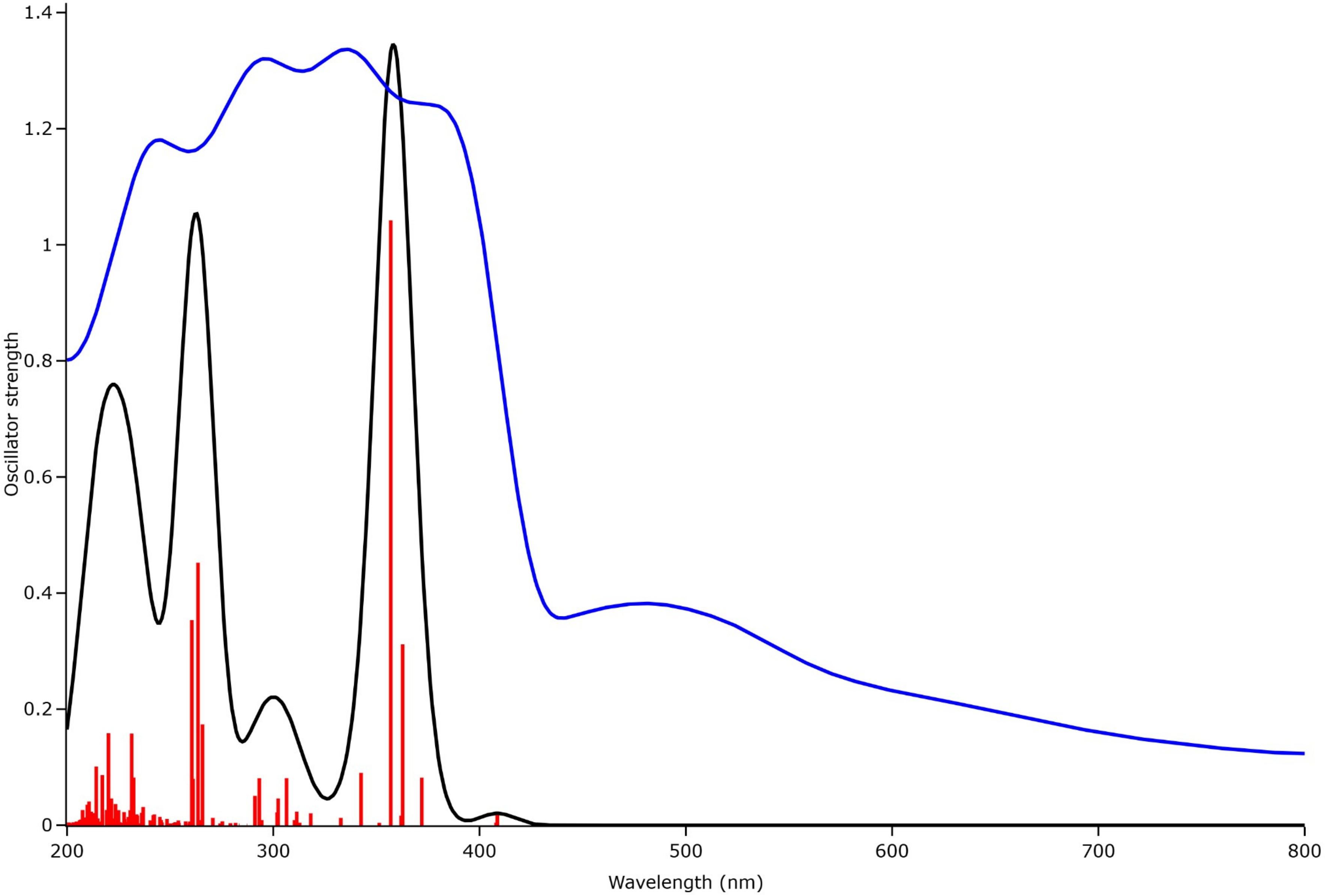

Figure S46. Experimental (blue) and calculated for 3 molecules (black) UV-Vis spectrum of **6**. Oscillator strengths are represented as vertical navy-red lines.

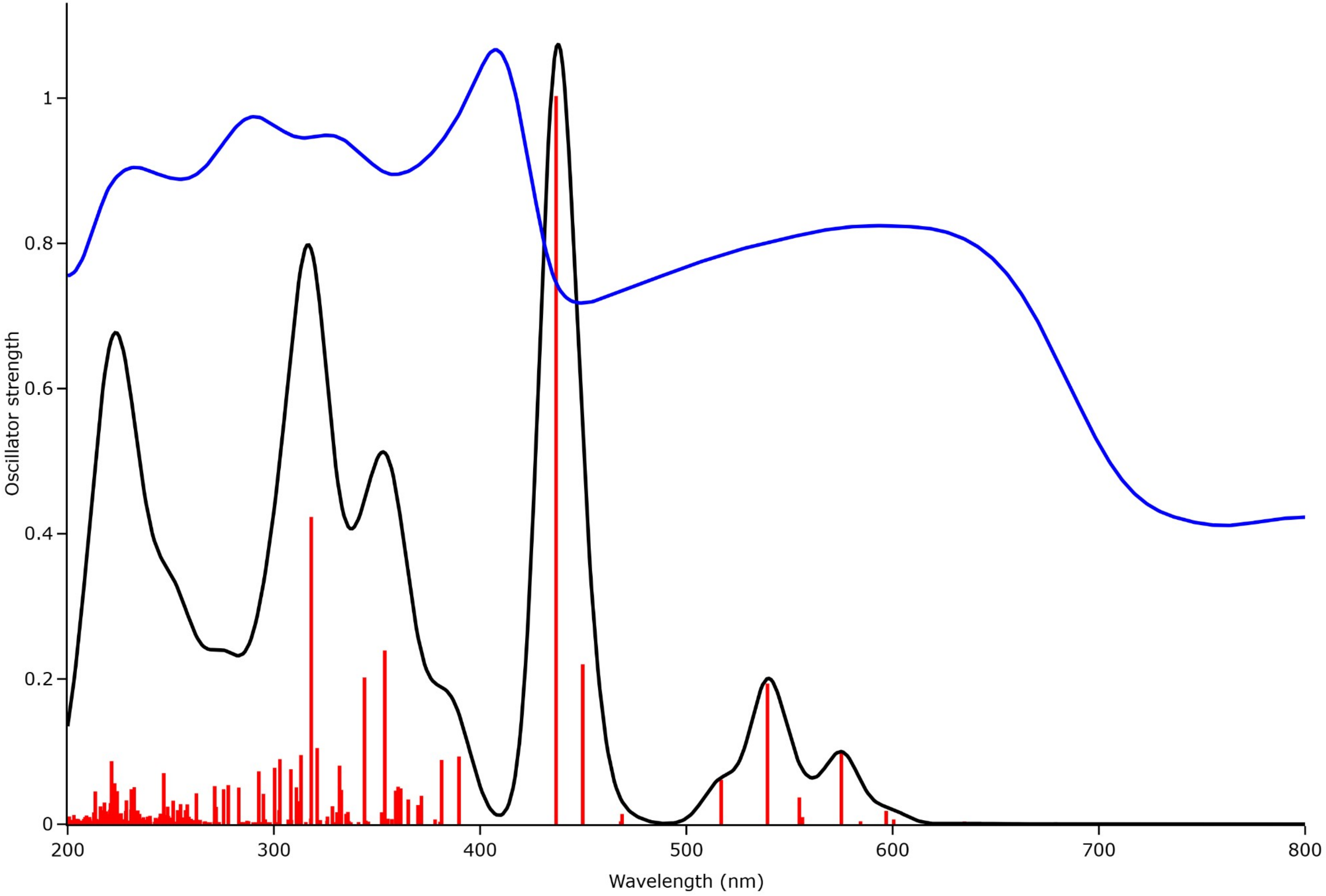

Figure S47. Experimental (blue) and calculated for 4 molecules (black) UV-Vis spectrum of **7**. Oscillator strengths are represented as vertical navy-red lines.

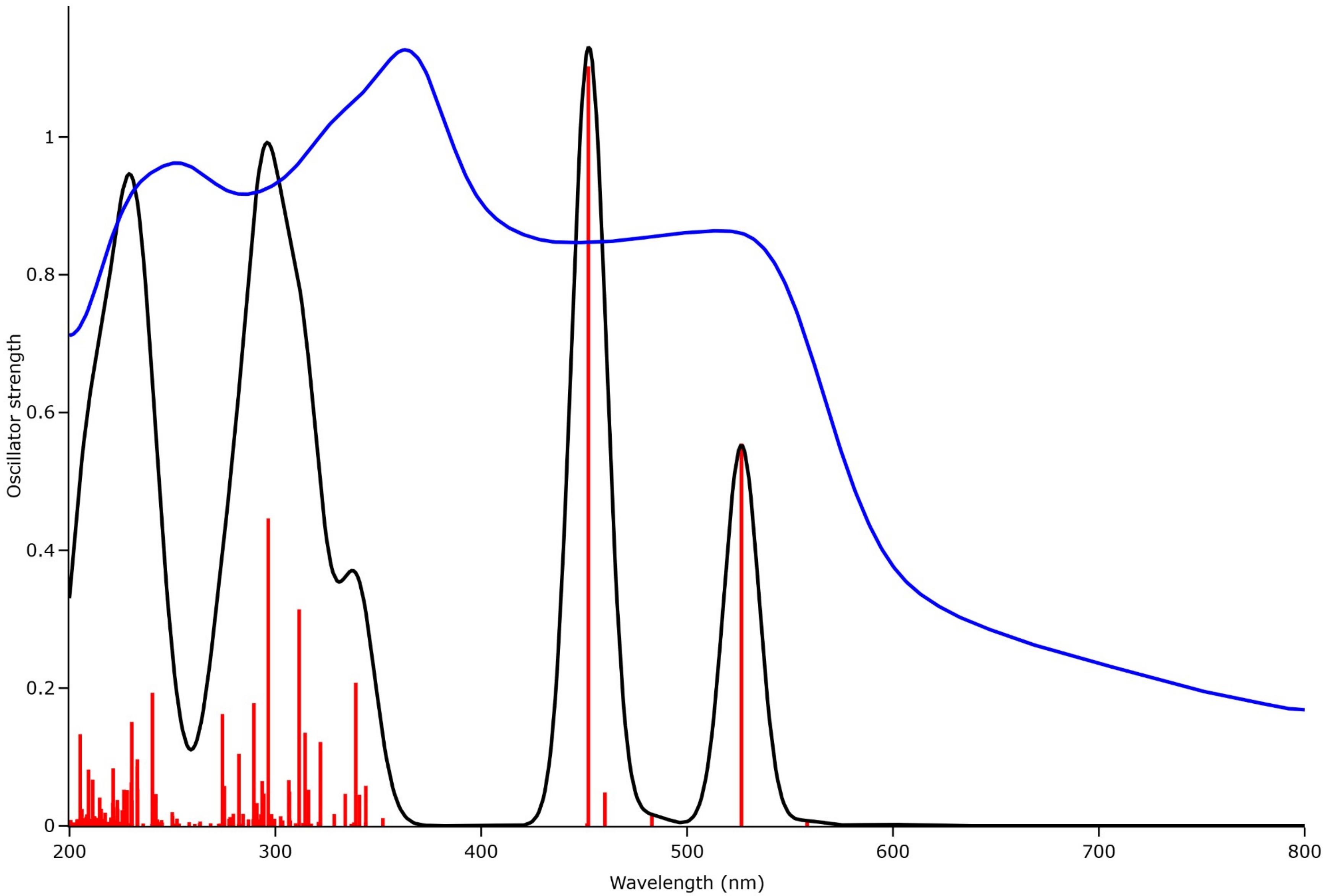

Figure S48. Experimental (blue) and calculated for 4 molecules (black) UV-Vis spectrum of **8**. Oscillator strengths are represented as vertical navy-red lines.

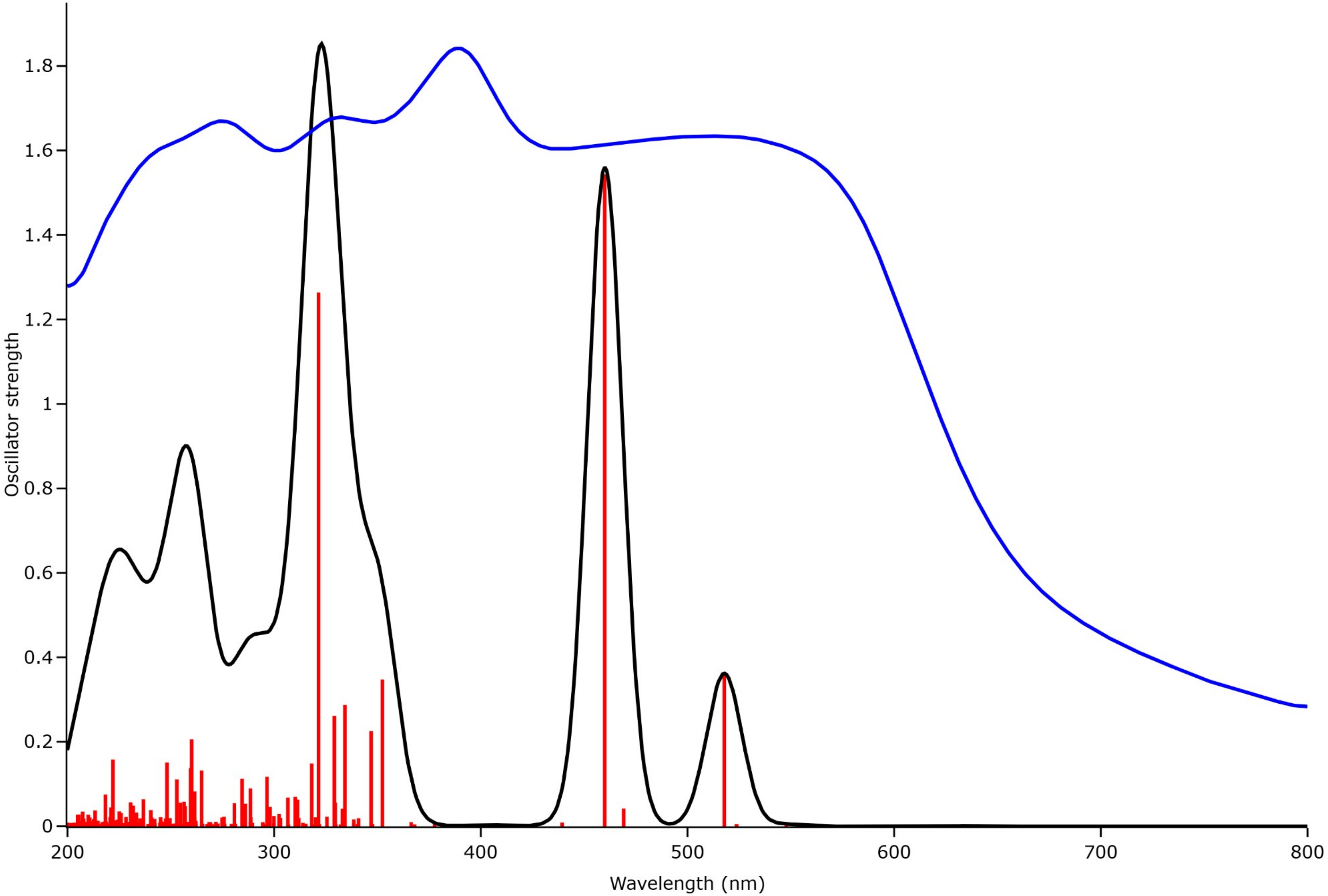

Figure S49. Experimental (blue) and calculated for 4 molecules (black) UV-Vis spectrum of **9**. Oscillator strengths are represented as vertical navy-red lines.

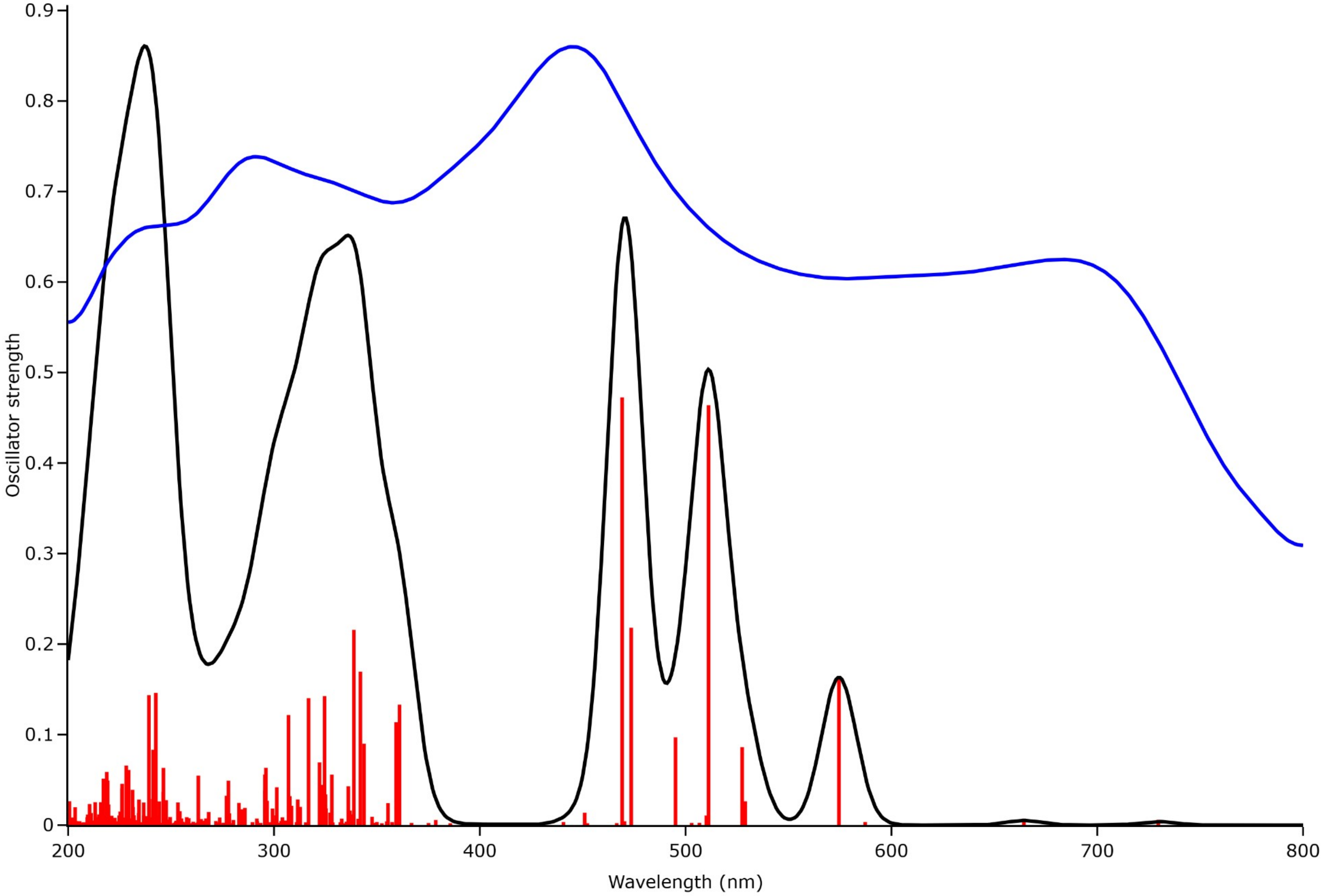

Figure S50. Experimental (blue) and calculated for 4 molecules (black) UV-Vis spectrum of **10**. Oscillator strengths are represented as vertical navy-red lines.

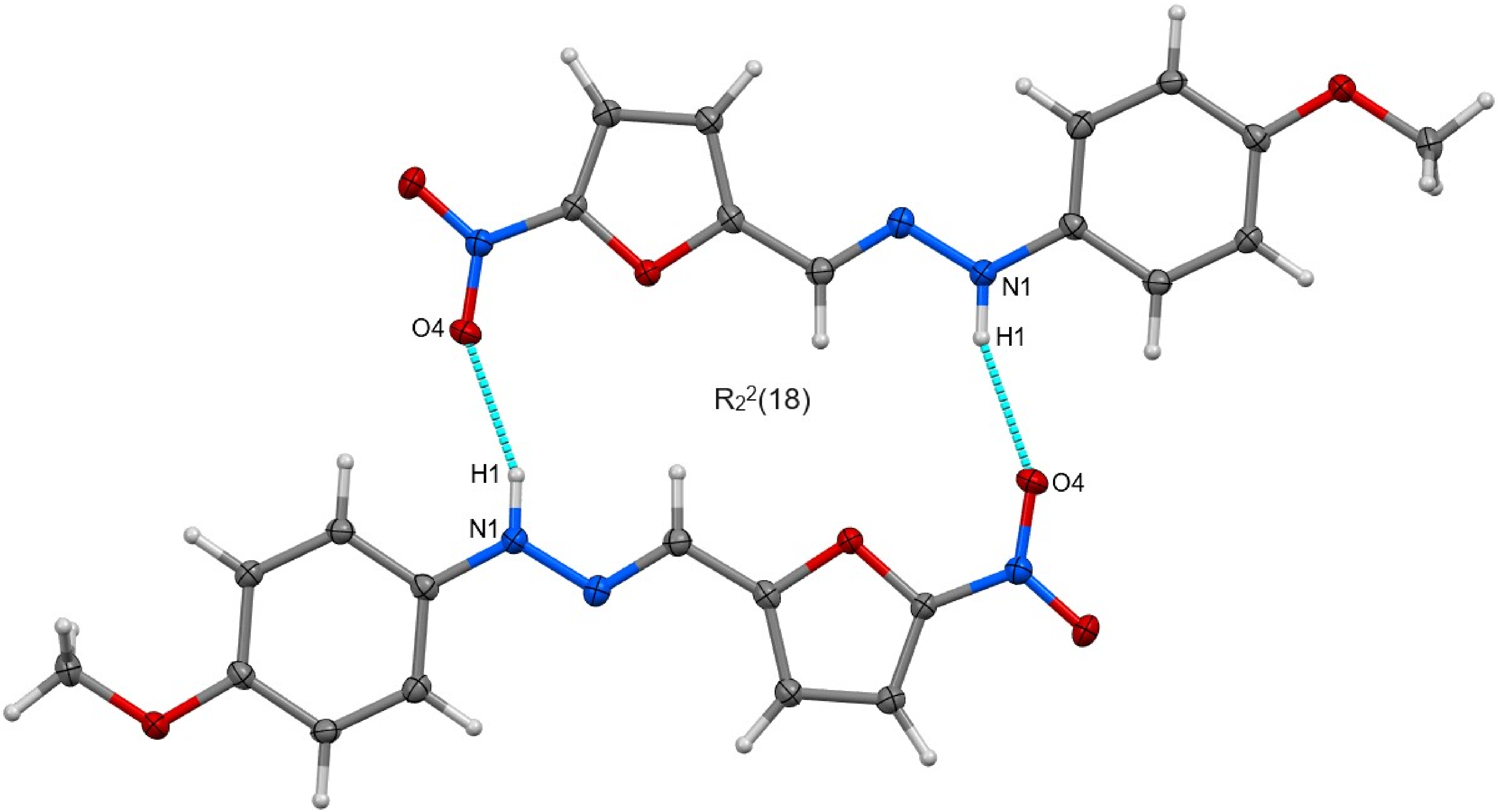

Figures S51. Hydrogen bond ring motif in the structure of **7**.

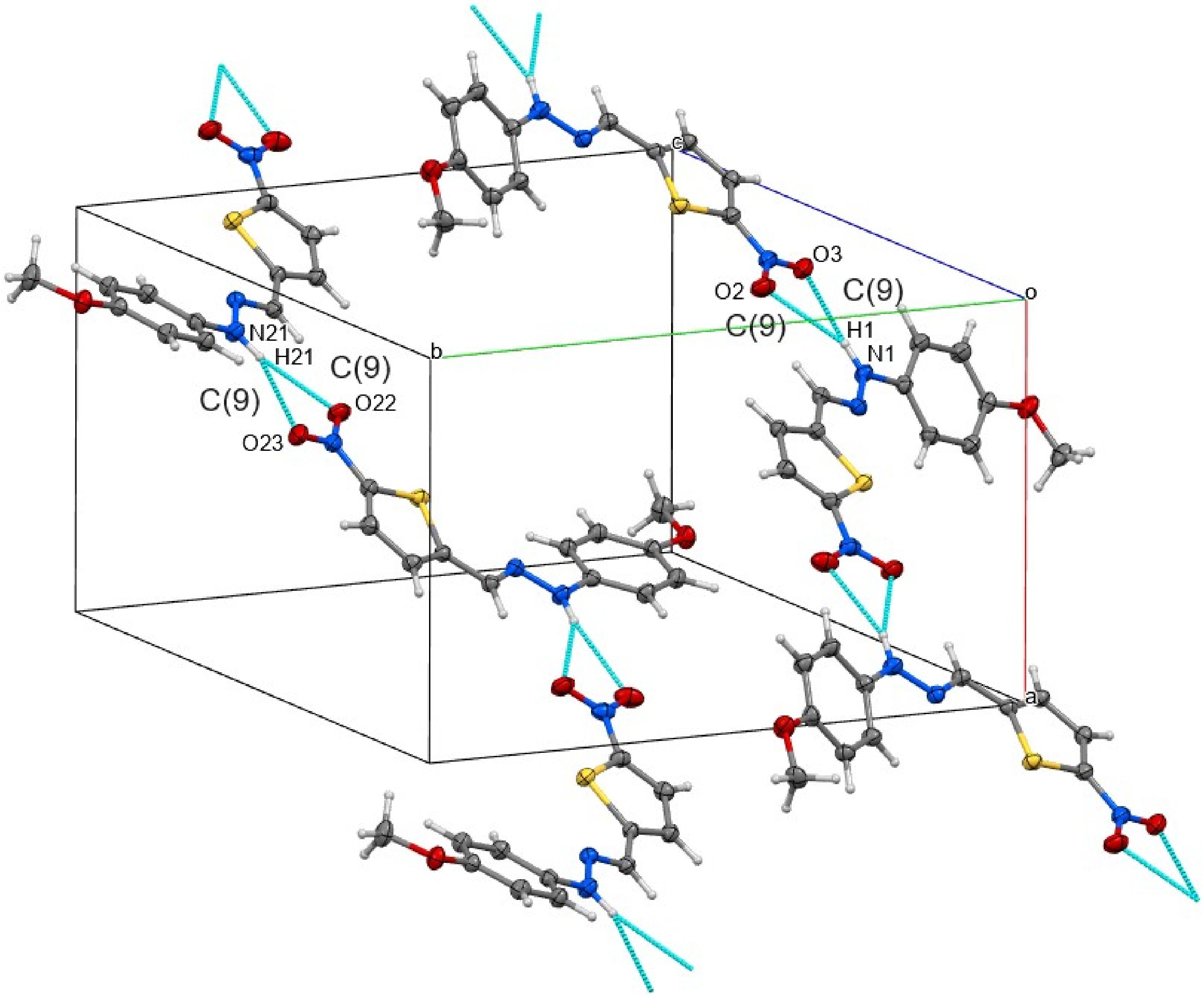

Figure S52. Hydrogen bond system in the structure of **10.**

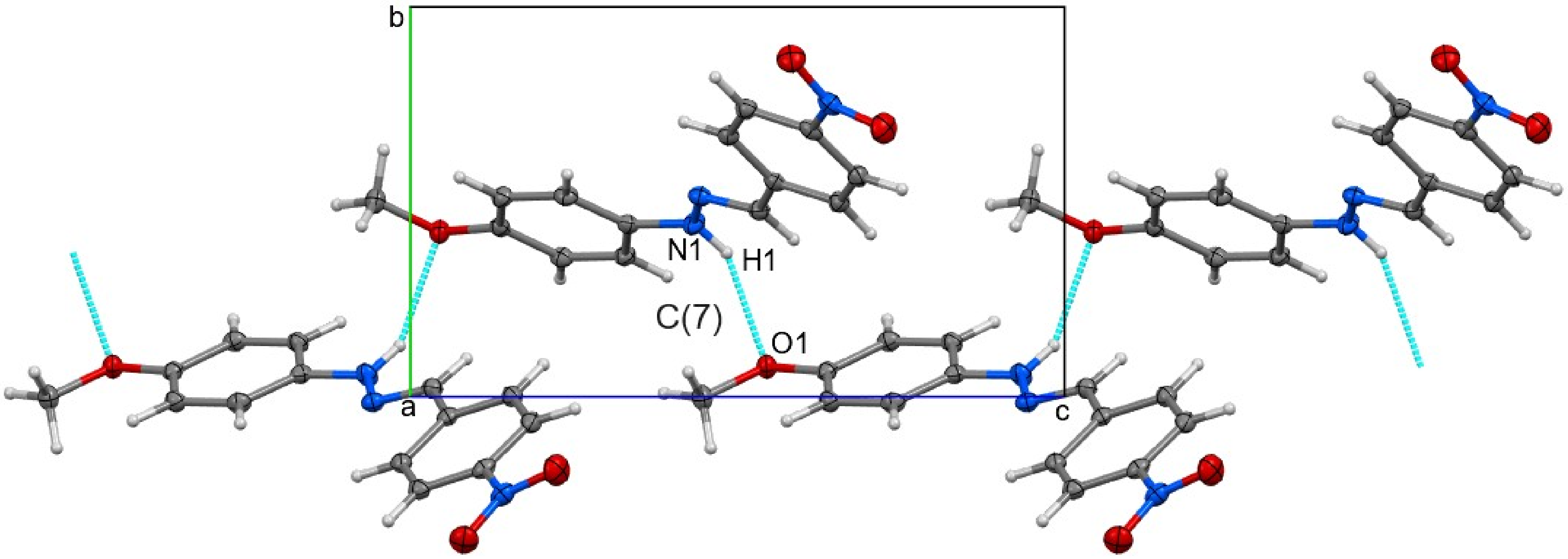

Figure S53. Hydrogen bond chain motif in the structure of **8.**

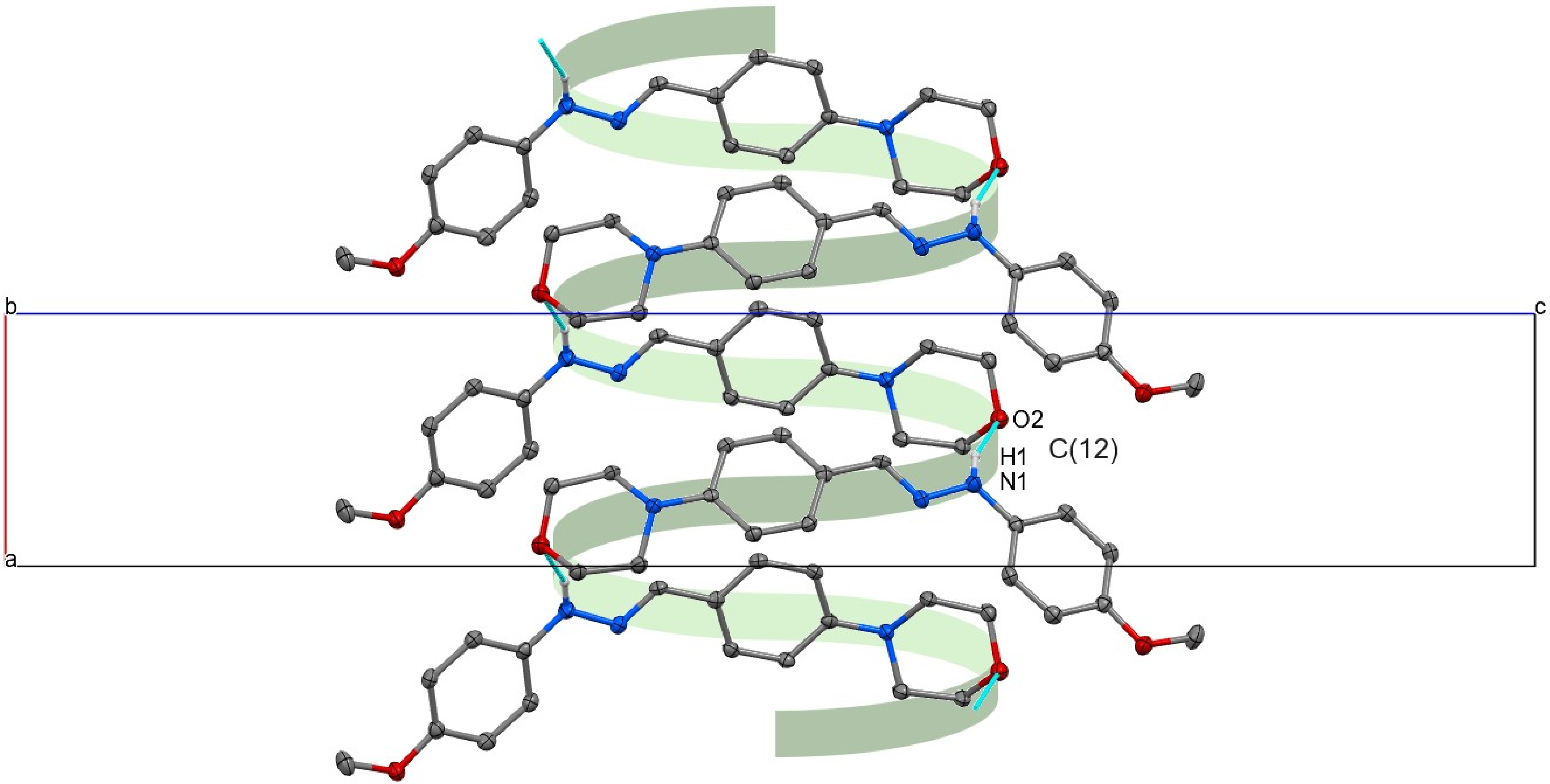

Figure S54. Hydrogen bond right-handed helix motif in the structure of **4**.

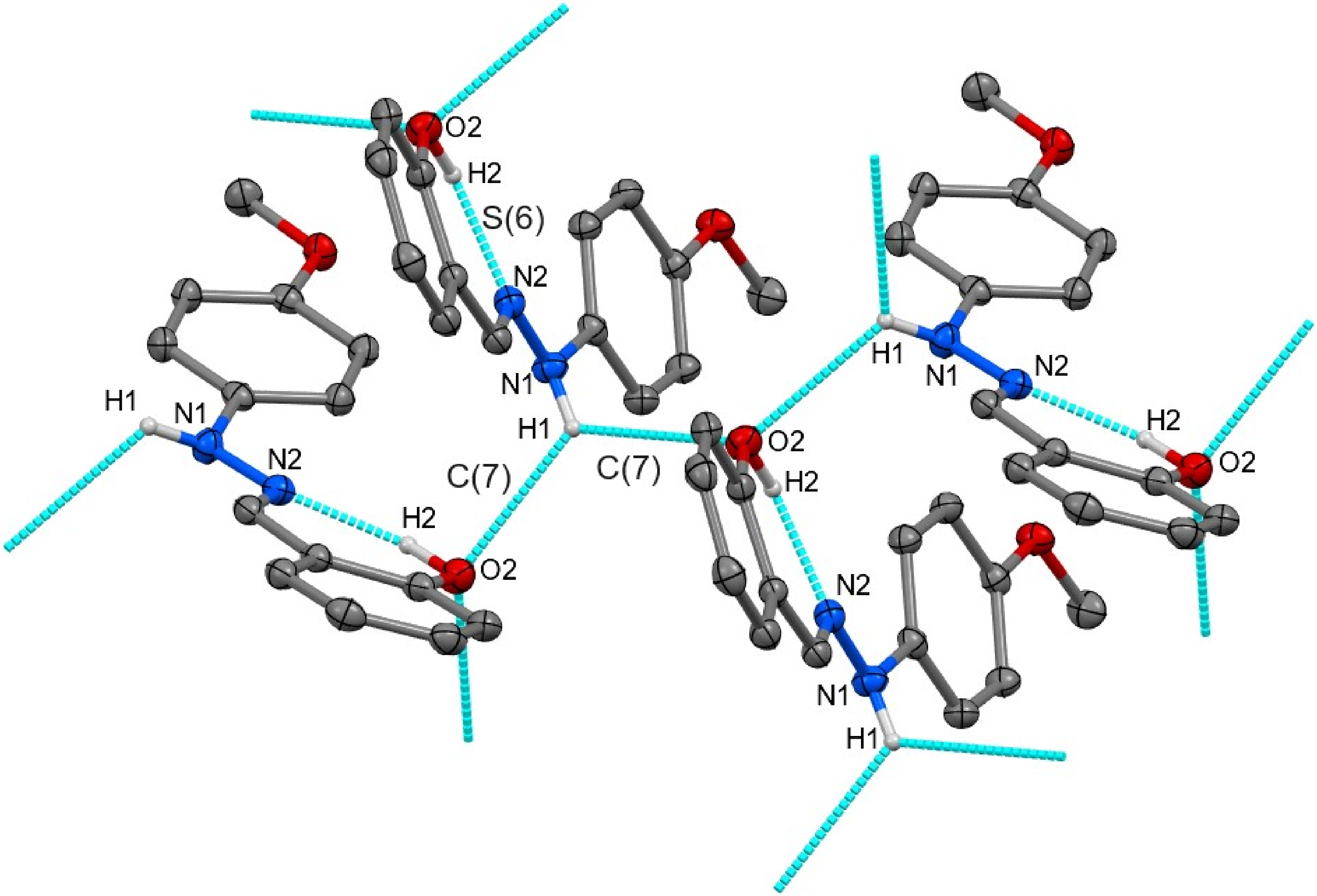

Figure S55. Hydrogen bond system in the structure of **3**.

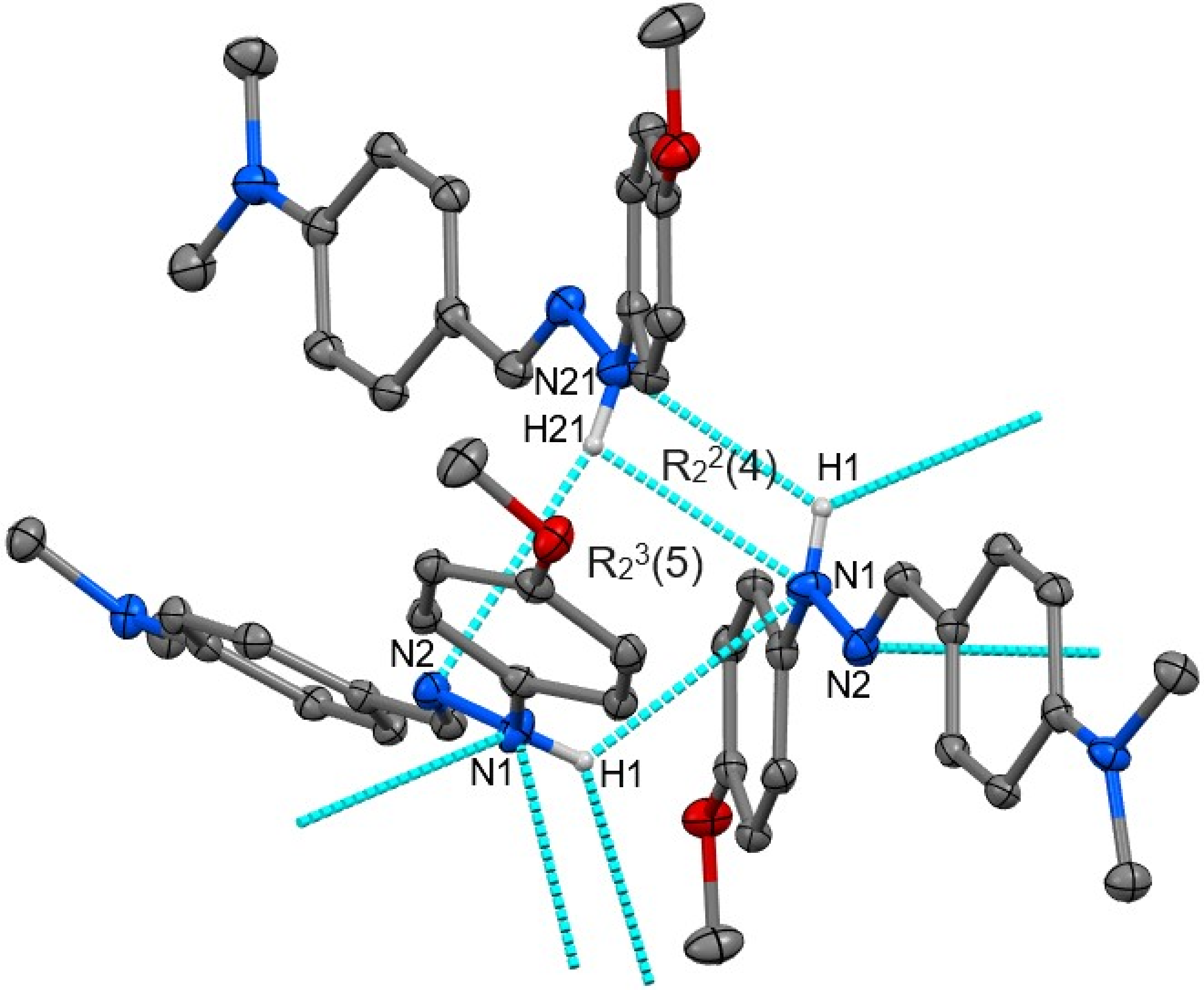

Figure S56. Hydrogen bond system in the structure of **2**.

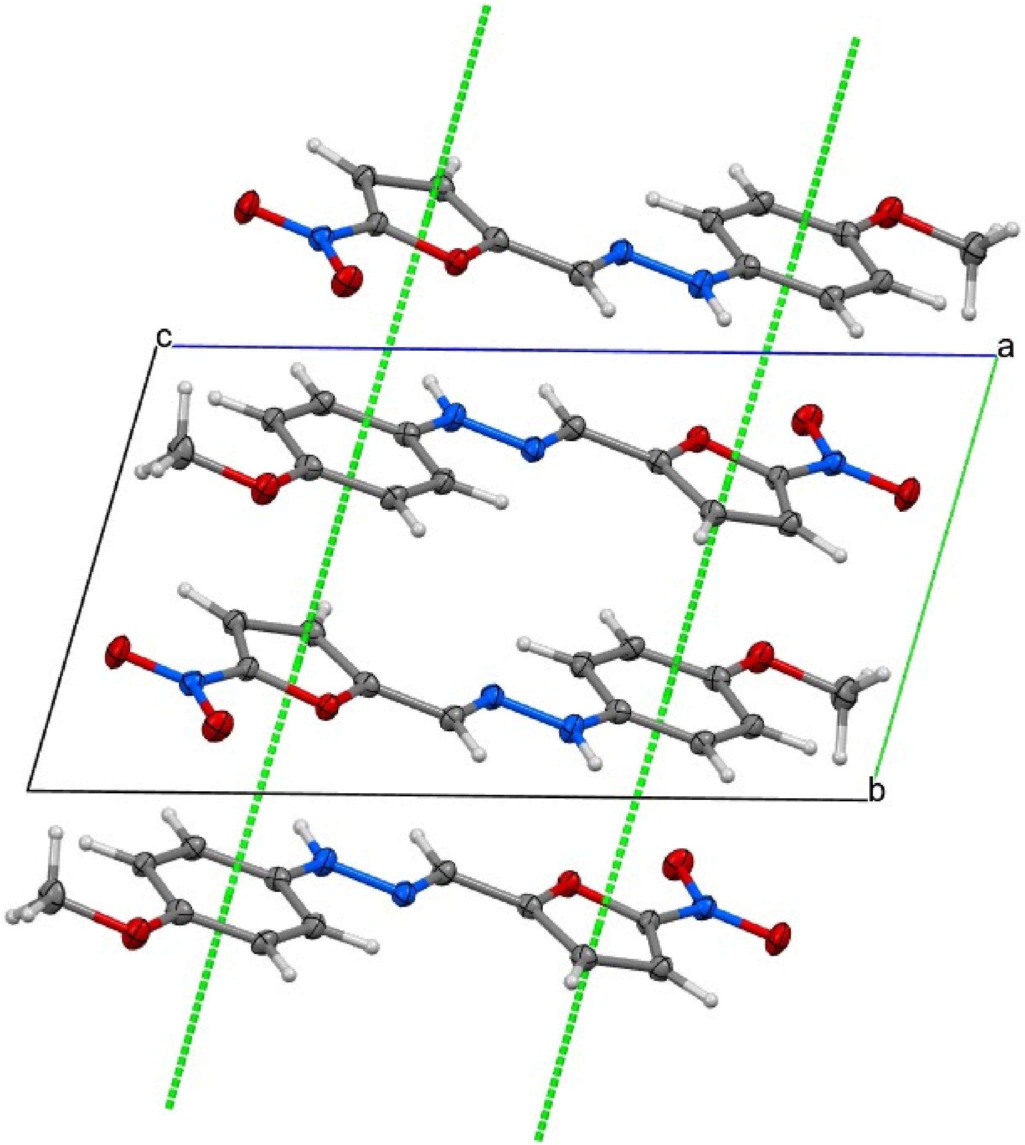

Figure S57. $\pi\cdots\pi$ stacking interactions in the structure of **7**.

8

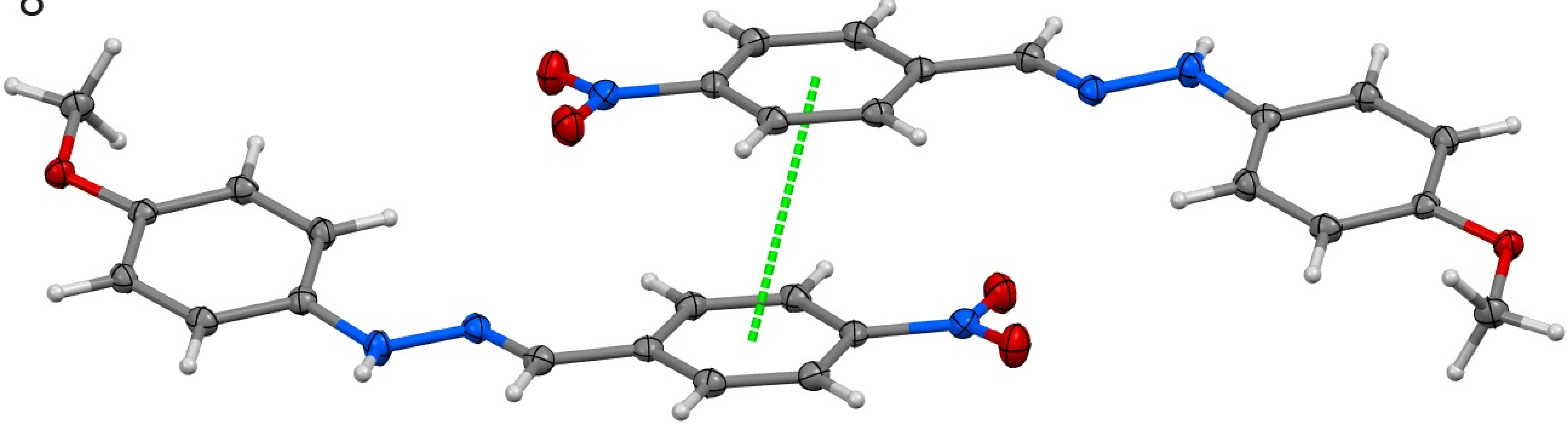

9

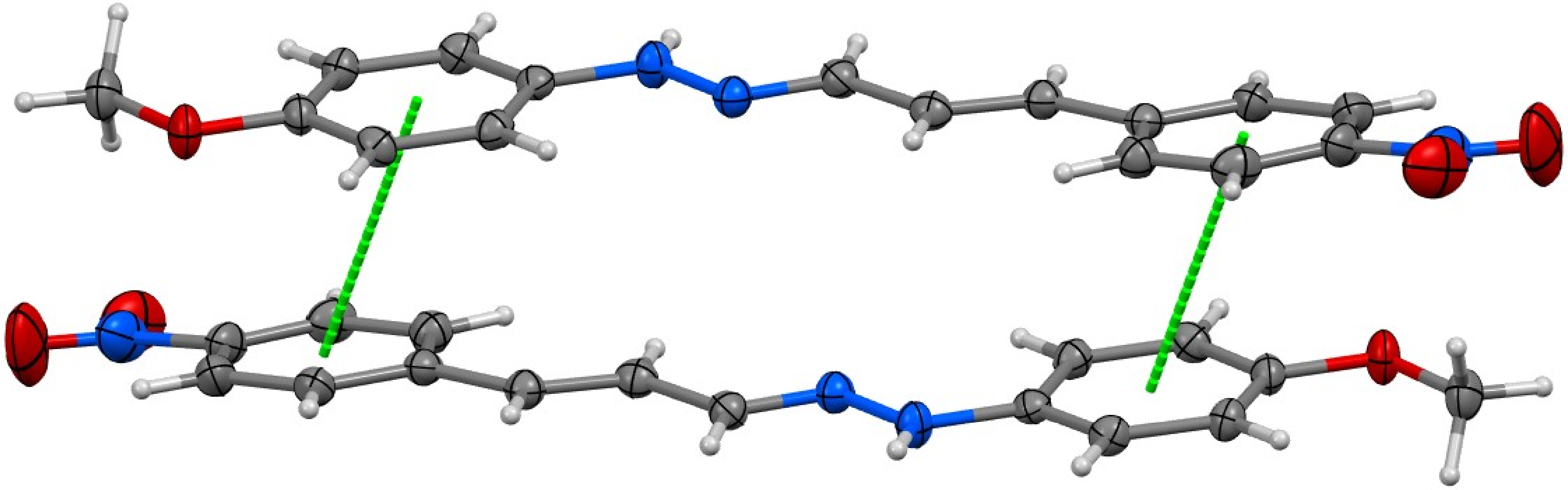

Figure S58. $\pi\cdots\pi$ stacking dimers in the structure of **8** and **9**.

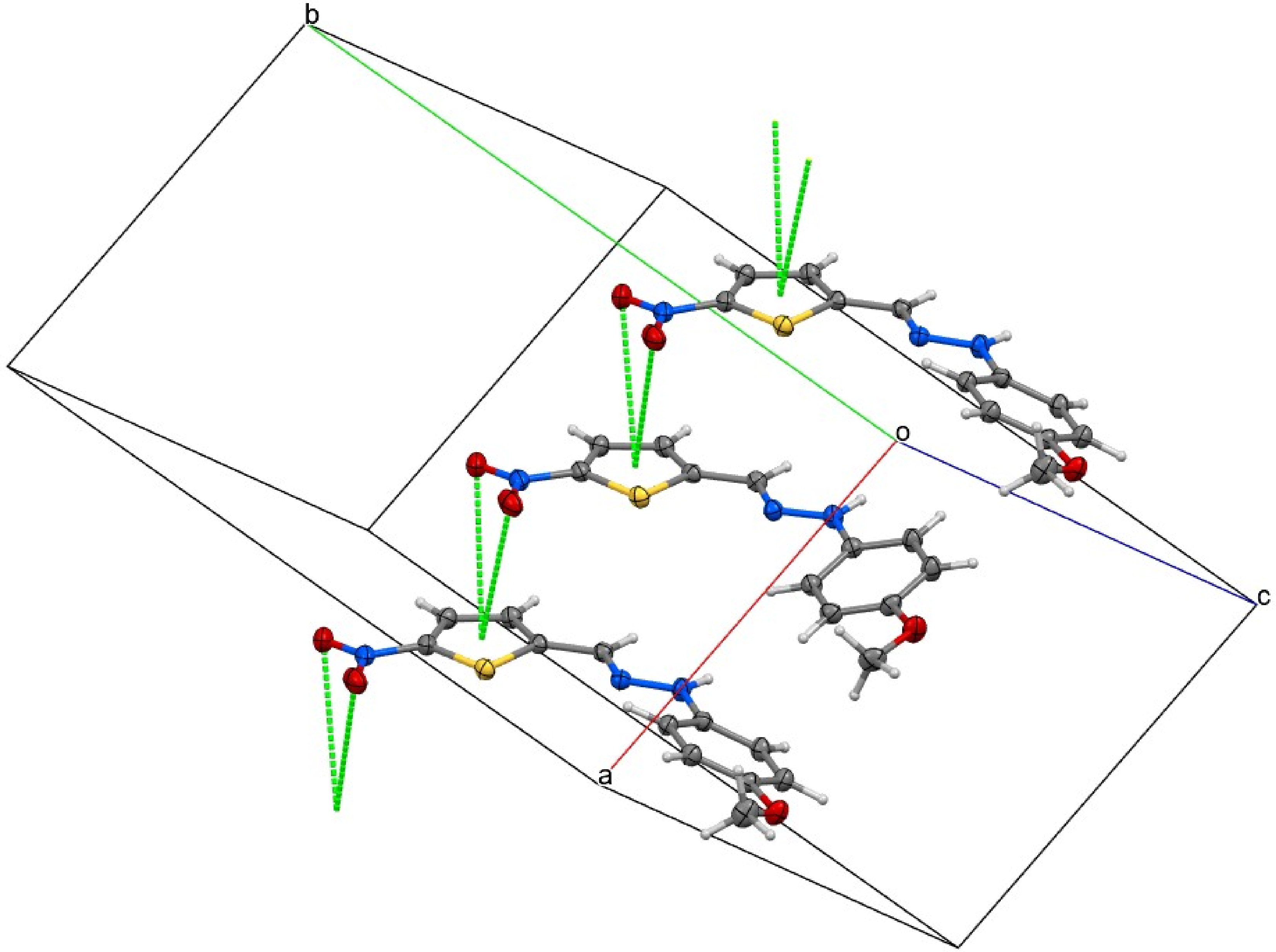

Figure S59. Anion···π interactions in the structure of **10**.

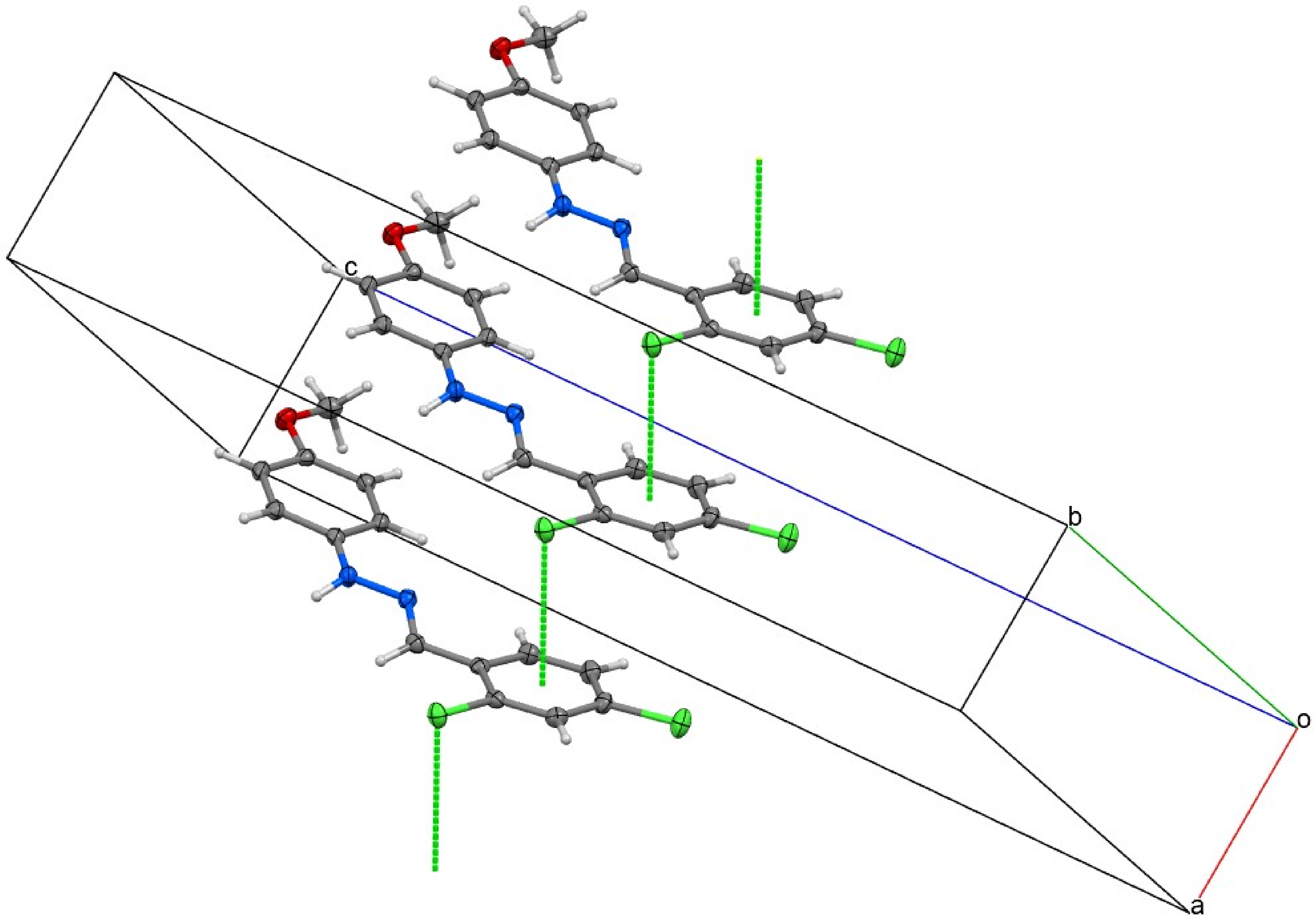

Figure S60. Anion···π interactions in the structure of **5**.

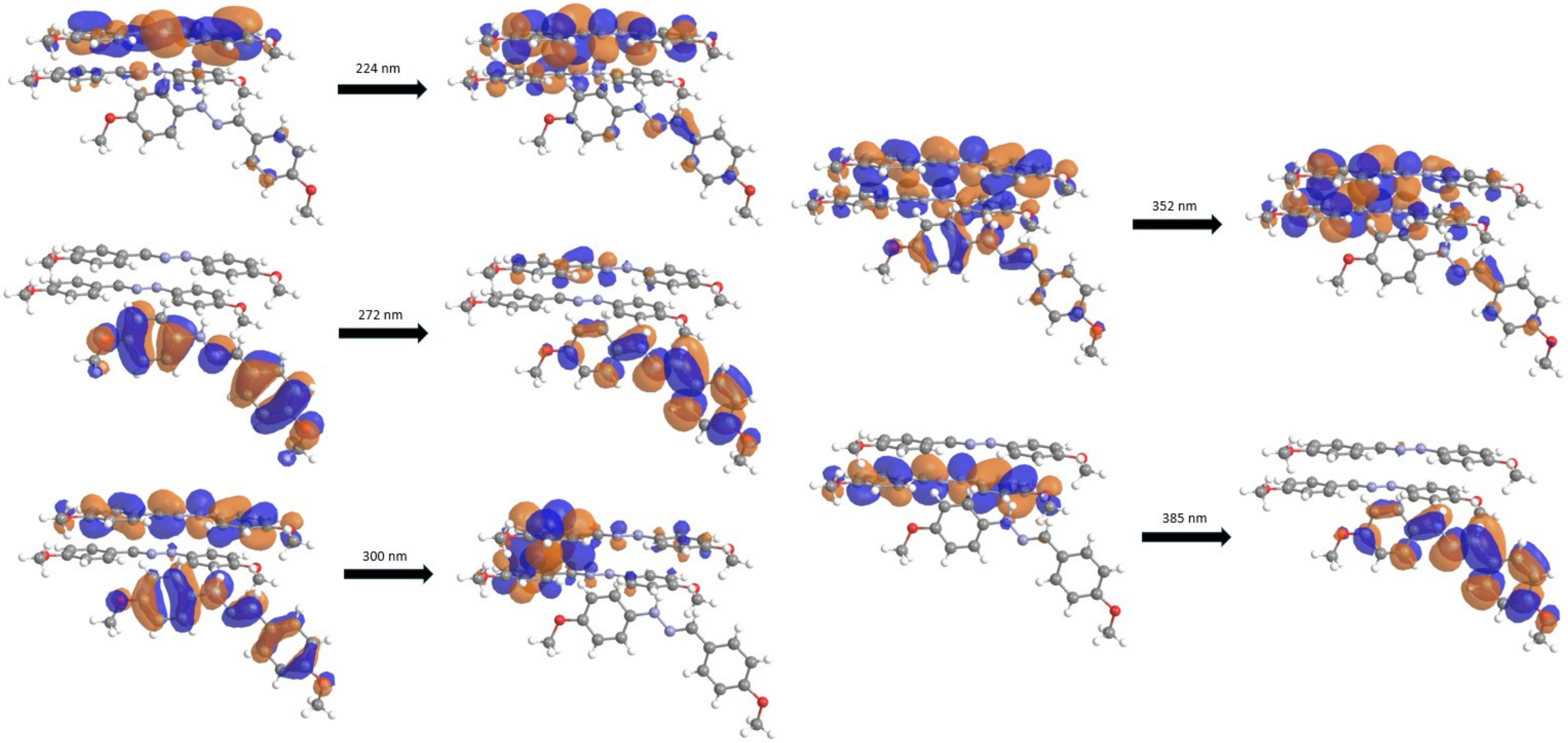

Figure S61. Calculated molecular orbital transition in the studied compound (**1**).

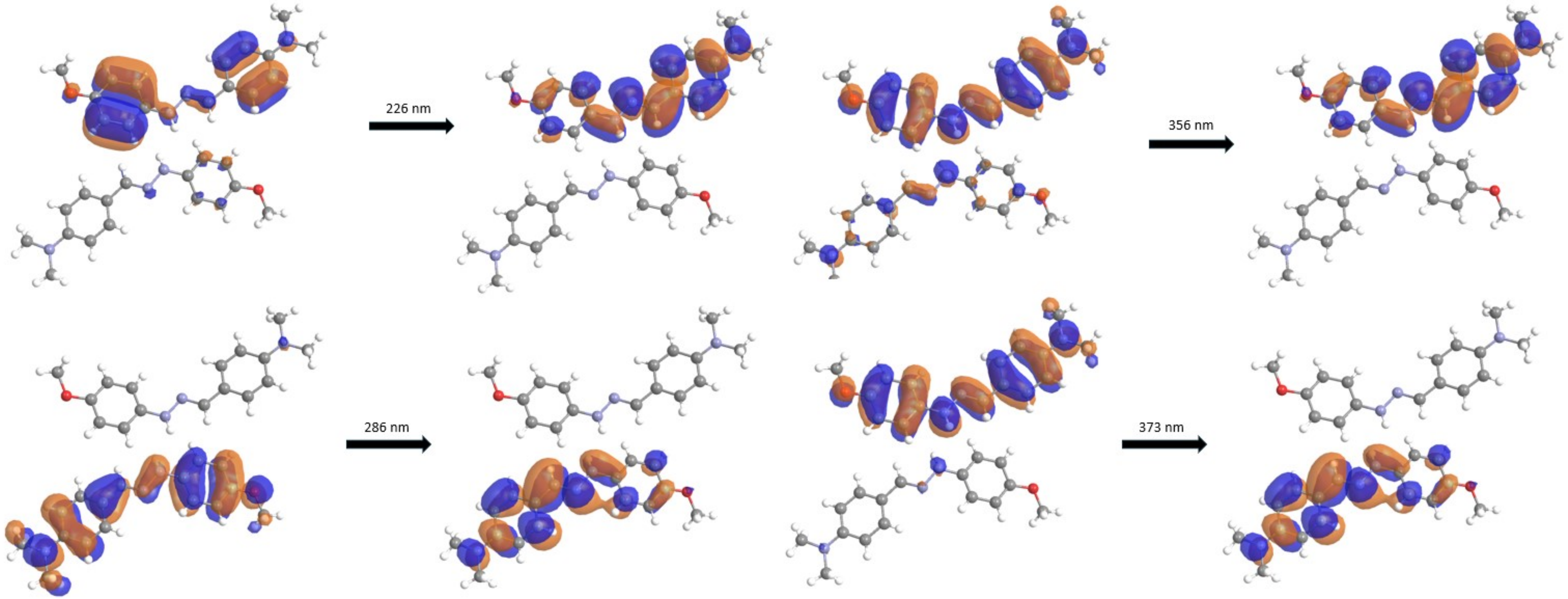

Figure S62. Calculated molecular orbital transition in the studied compound (**2**).

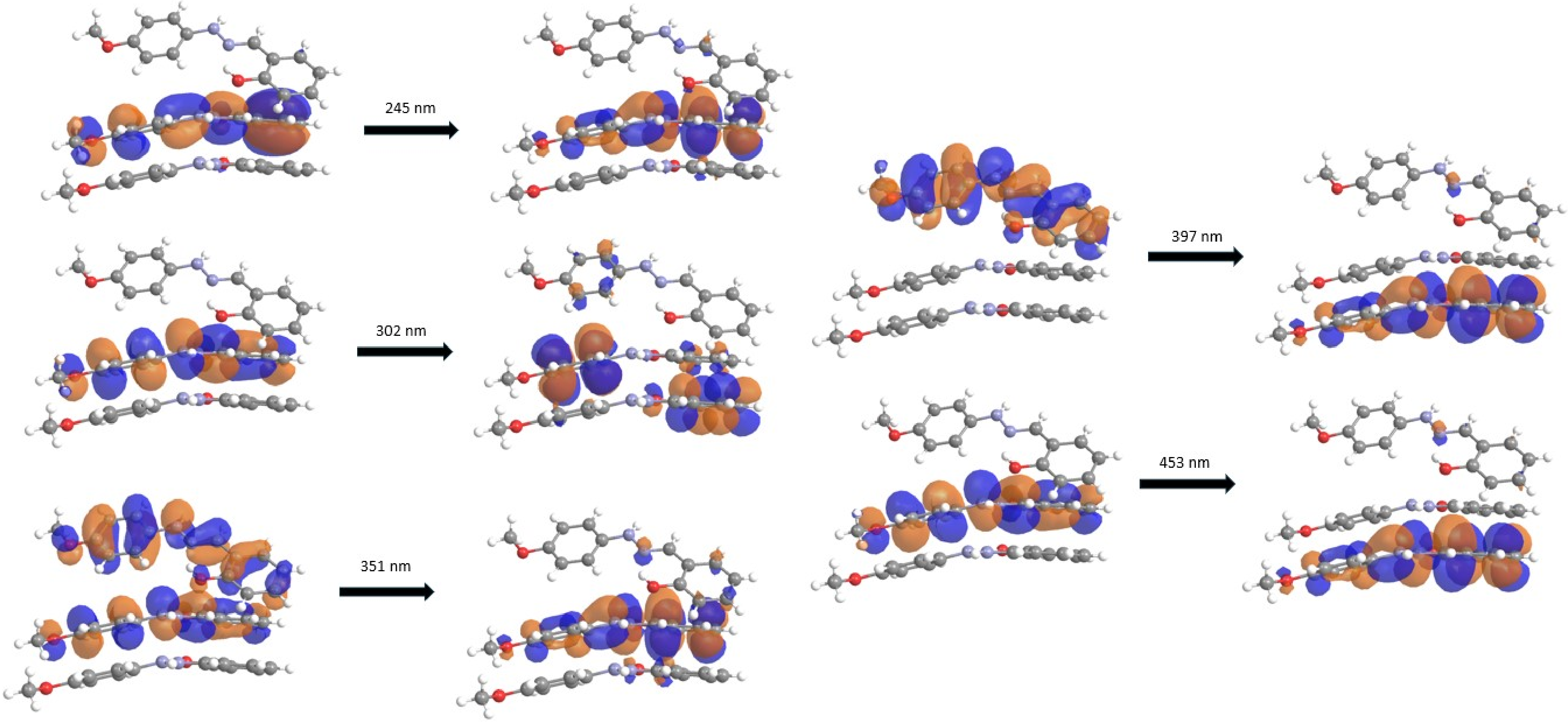

Figure S63. Calculated molecular orbital transition in the studied compound (**3**).

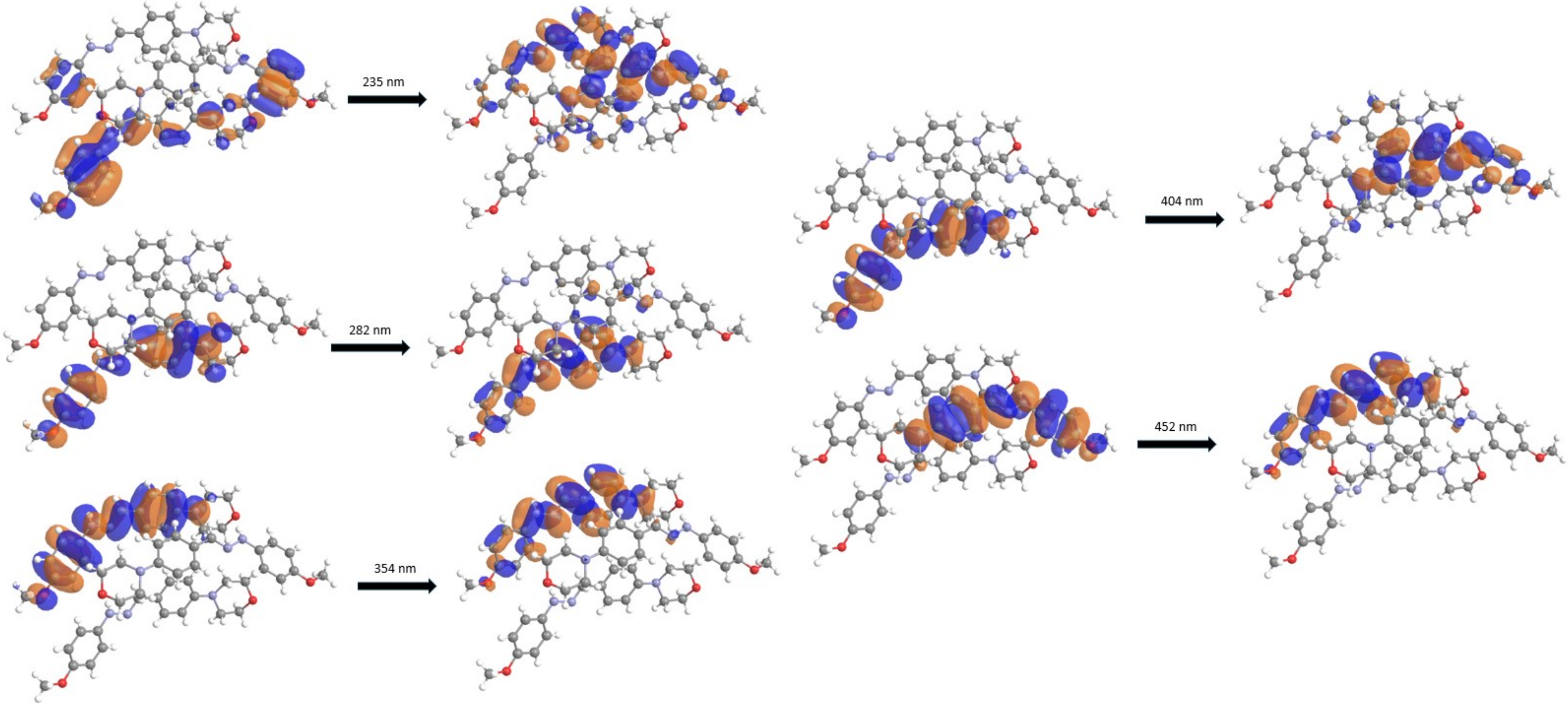

Figure S64. Calculated molecular orbital transition in the studied compound (**4**).

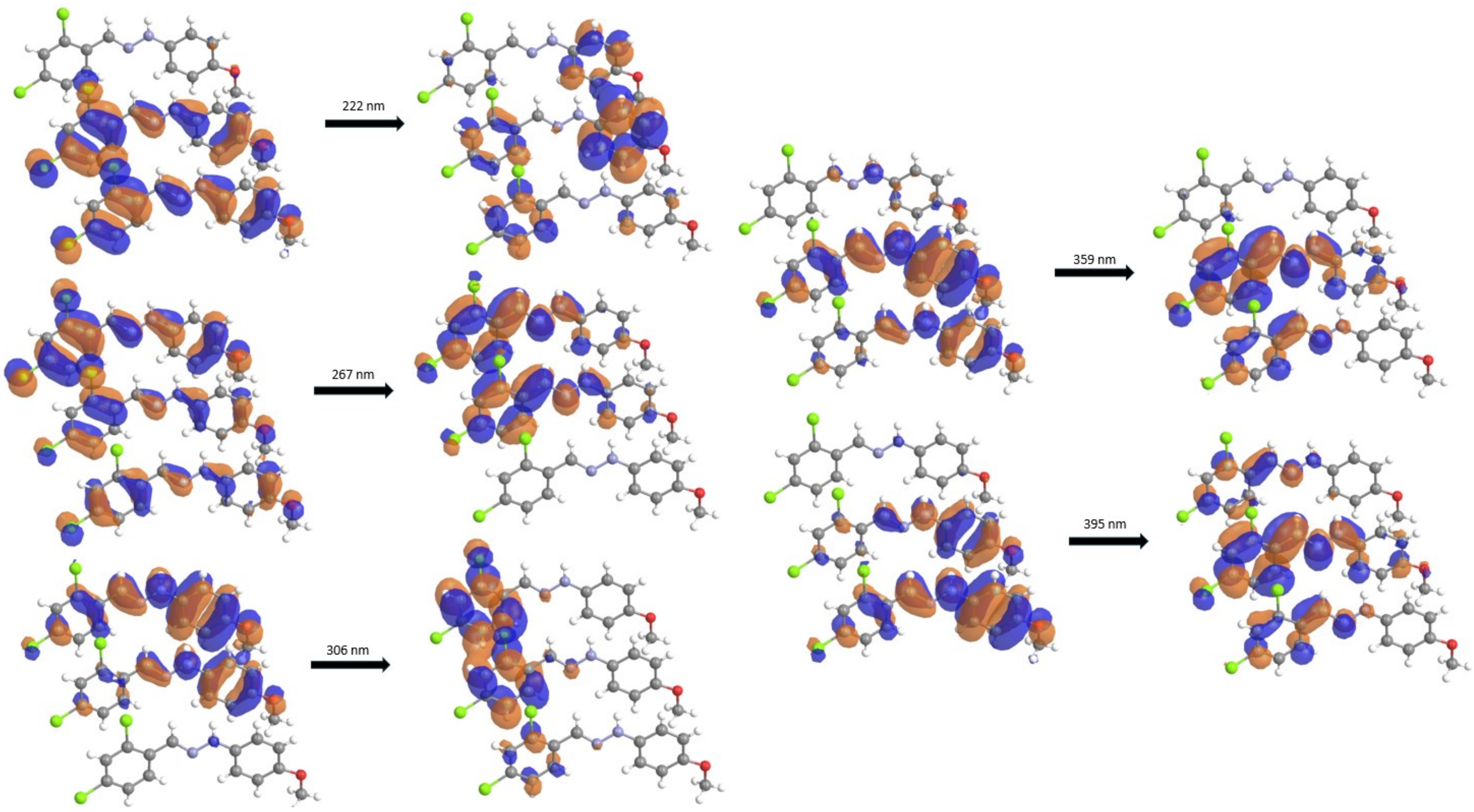

Figure S65. Calculated molecular orbital transition in the studied compound (**5**).

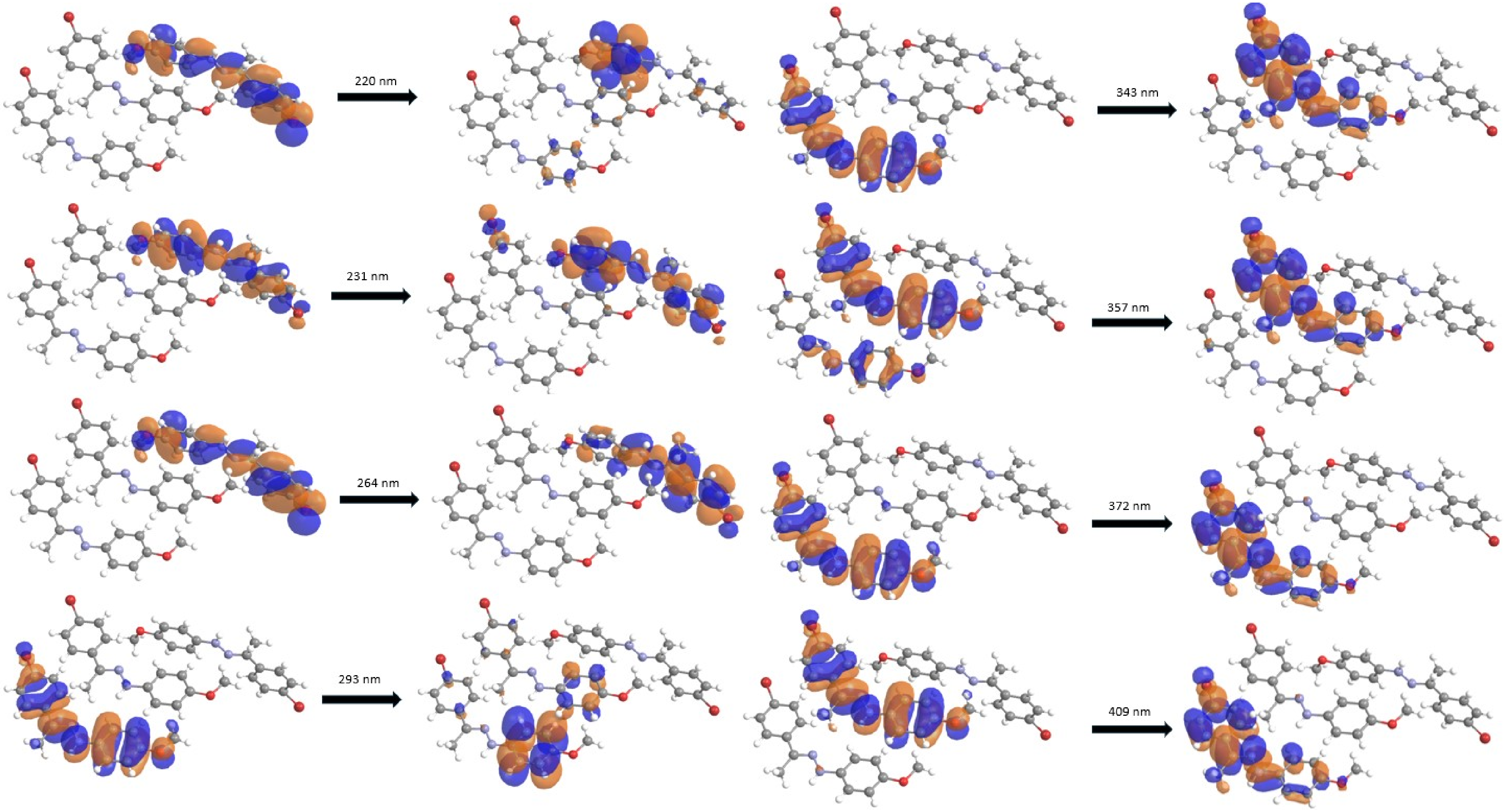

Figure S66. Calculated molecular orbital transition in the studied compound (**6**).

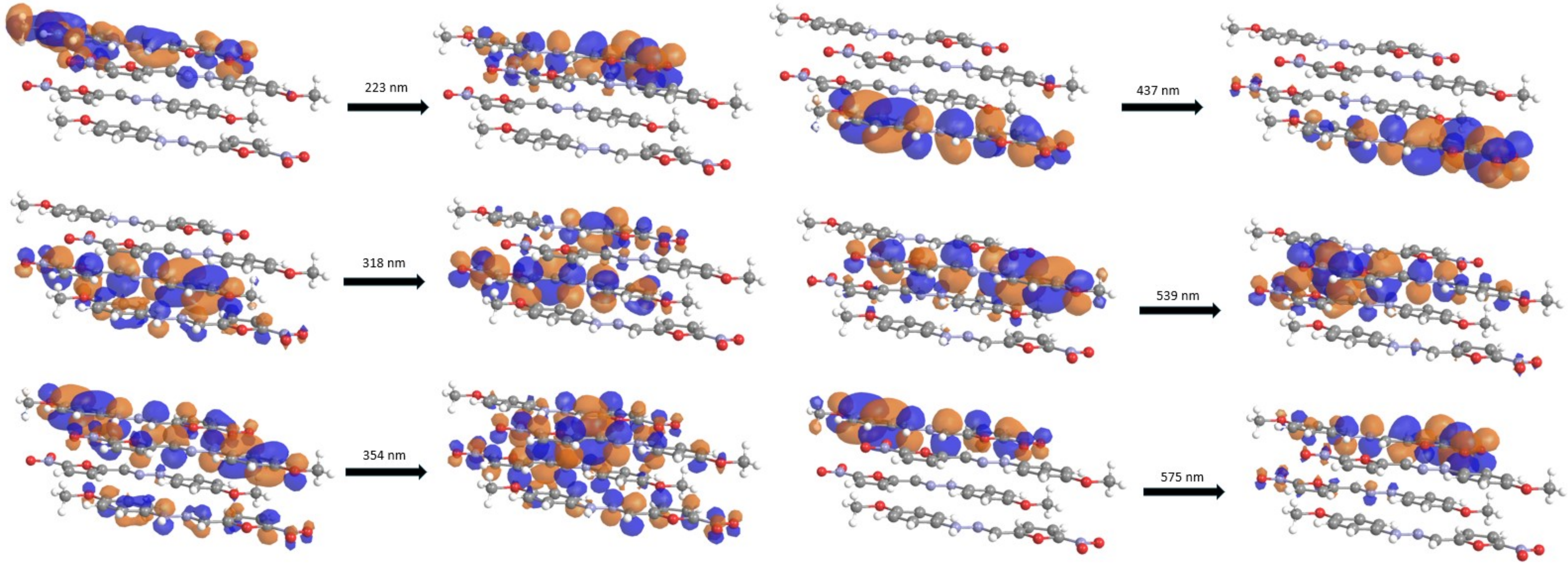

Figure S67. Calculated molecular orbital transition in the studied compound (7).

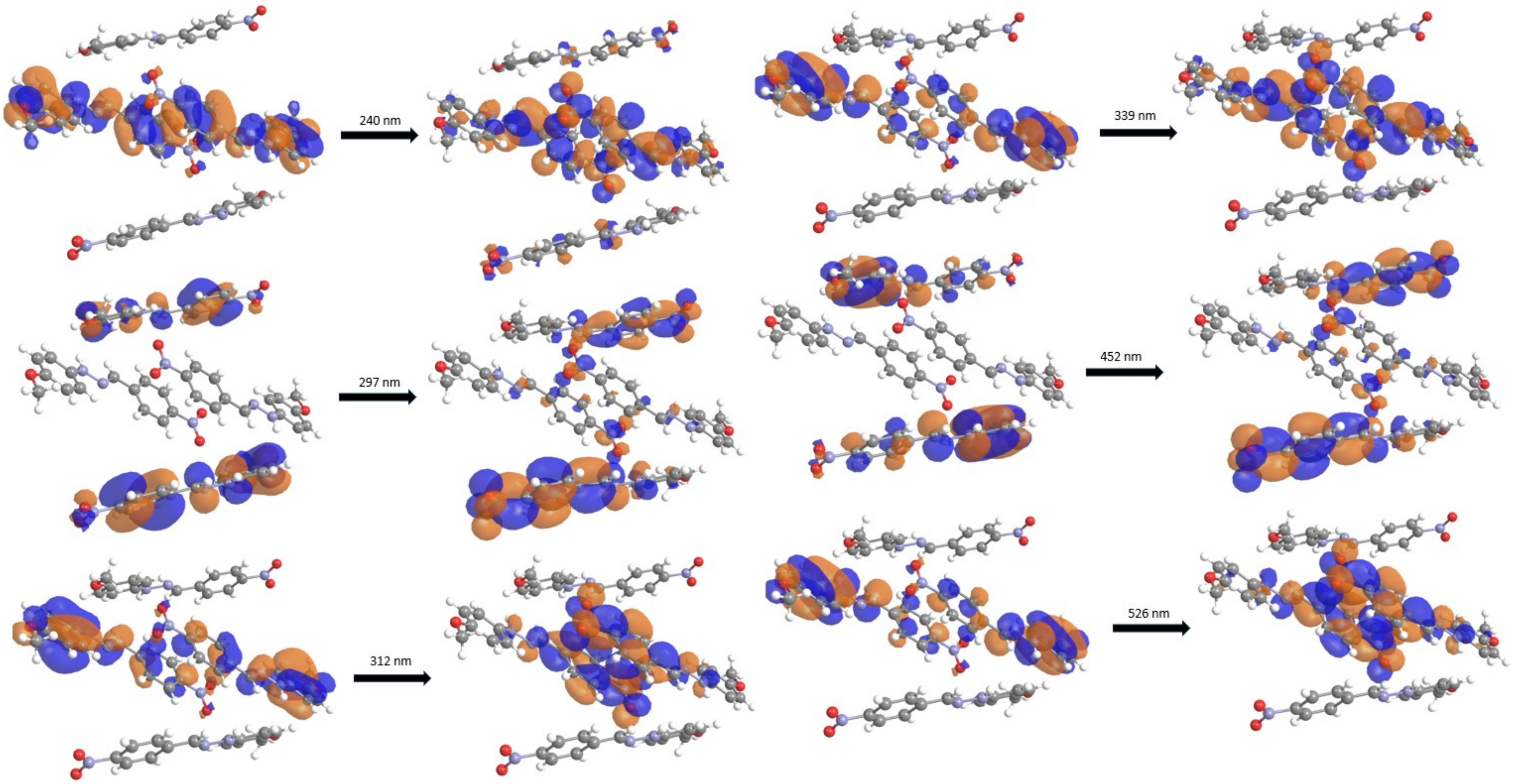

Figure S68. Calculated molecular orbital transition in the studied compound (8).

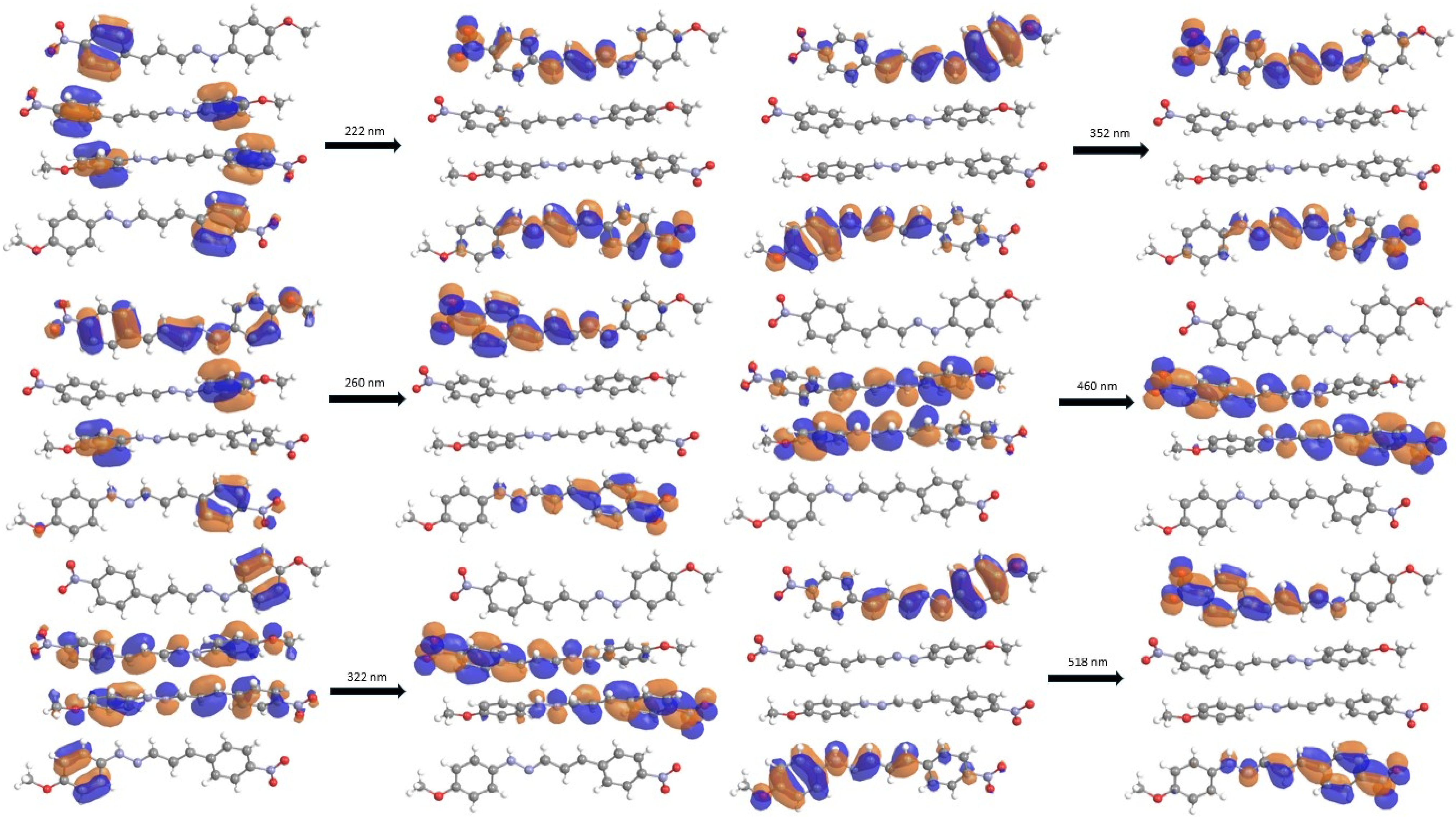

Figure S69. Calculated molecular orbital transition in the studied compound (9).

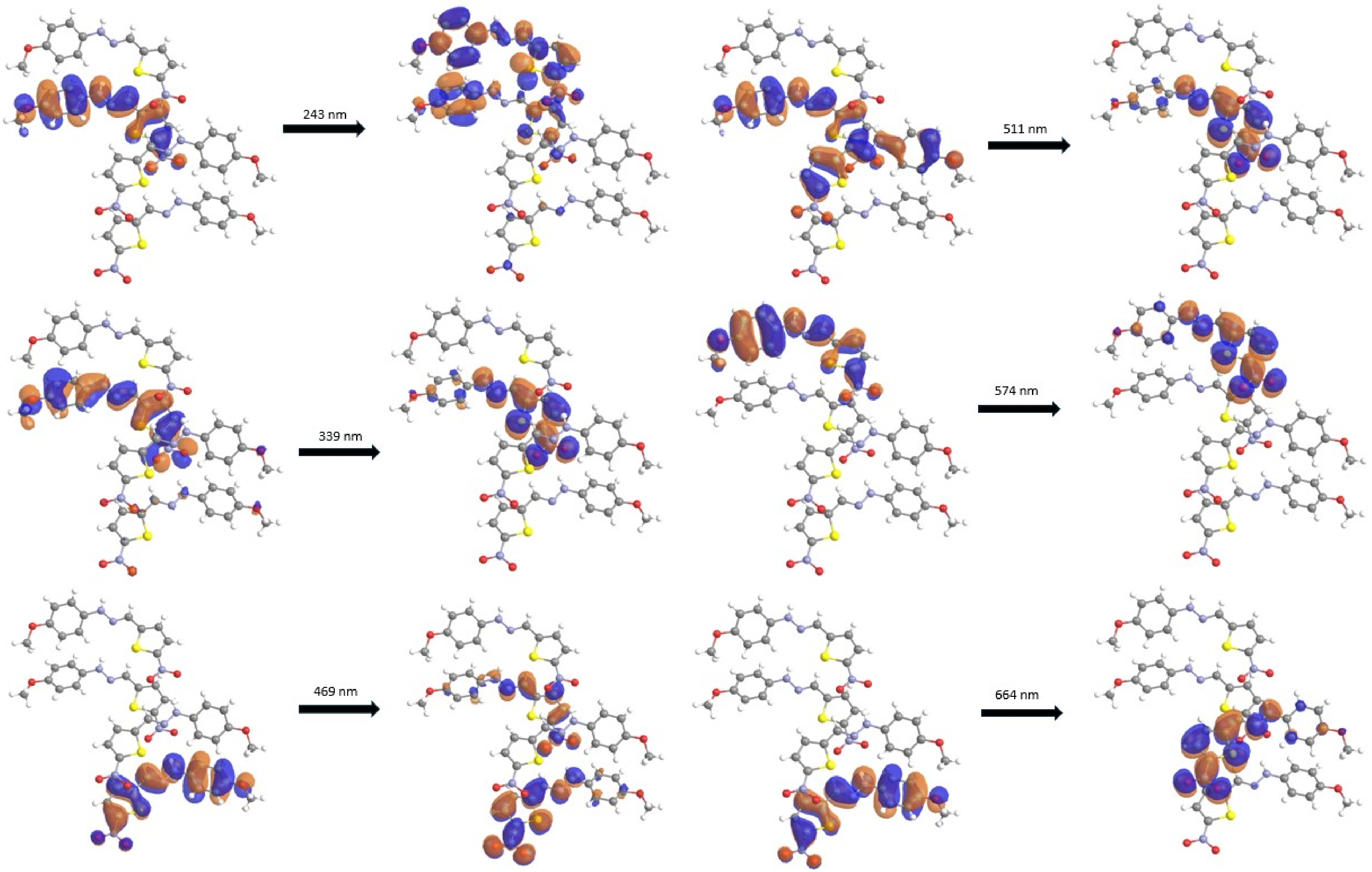

Figure S70. Calculated molecular orbital transition in the studied compound (10).

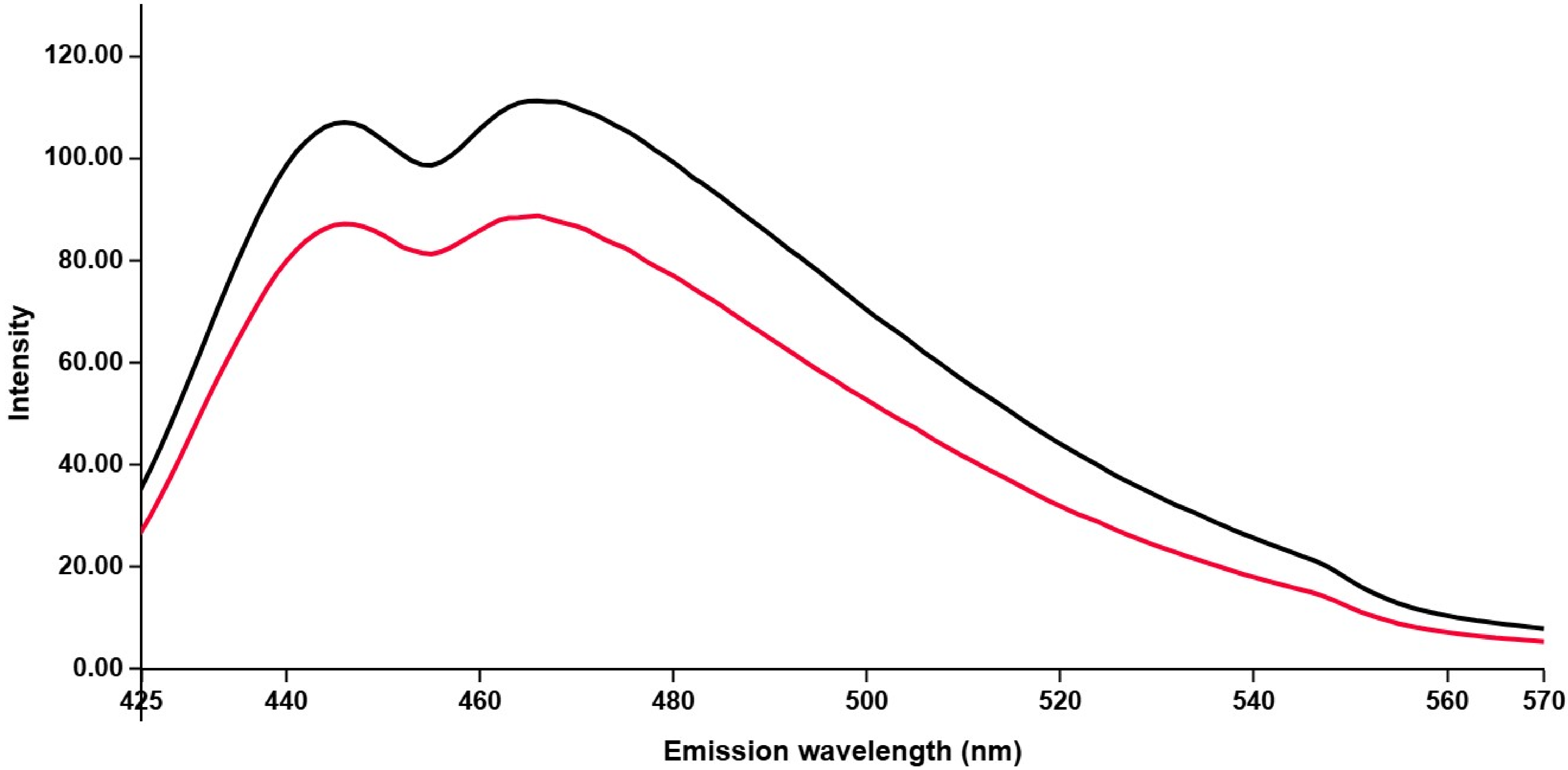

Figure S71. 2D fluorescence emission spectra for compound 1 at two excitation wavelengths: black line at λ_{ex} = 415 nm and red line at λ_{ex}= 364 nm, showing intensity as a function of emission wavelength.

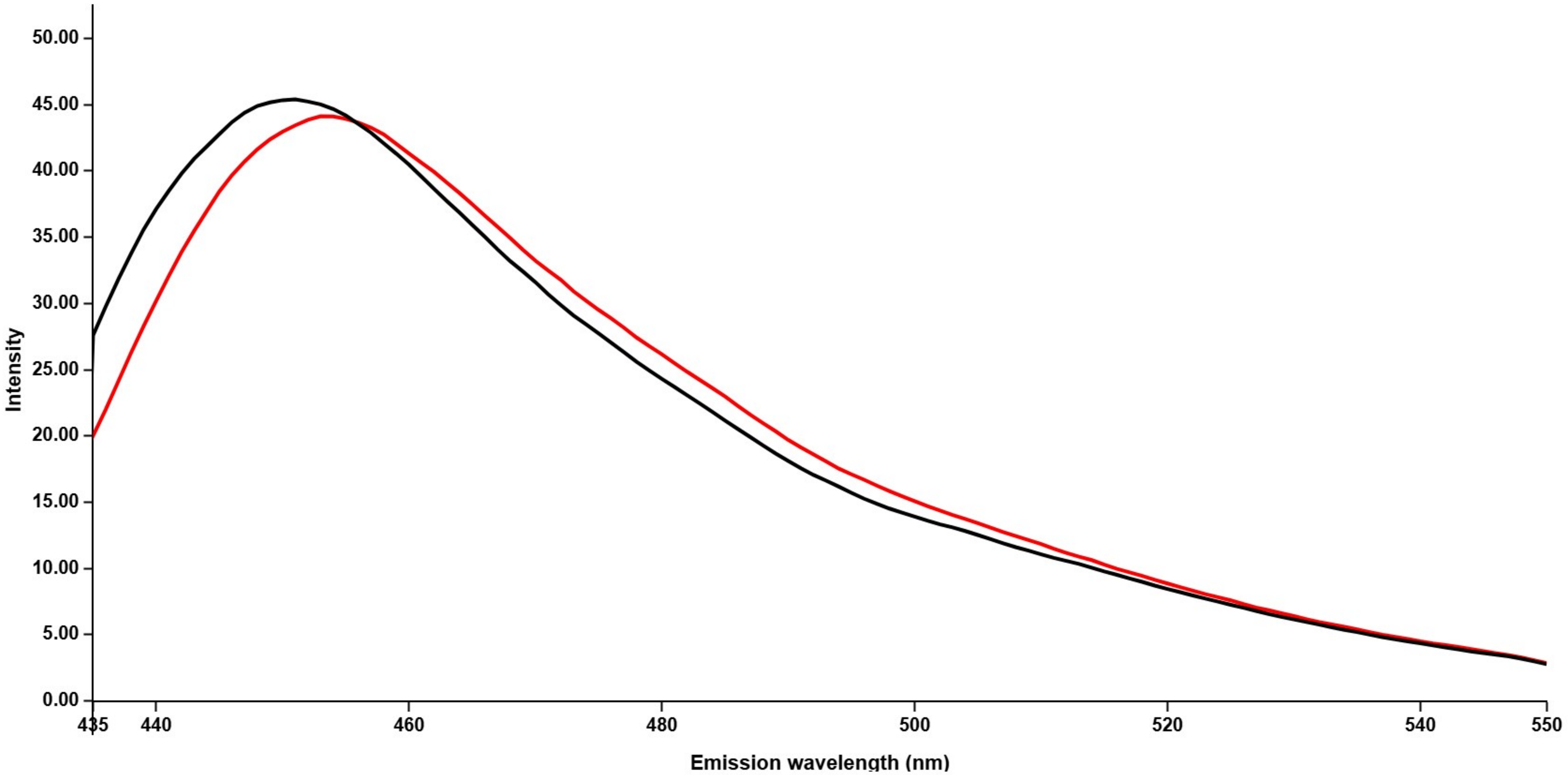

Figure S72. 2D fluorescence emission spectra for compound 2 at two excitation wavelengths: black line at λ_{ex} = 428 nm and red line at λ_{ex}= 359 nm, showing intensity as a function of emission wavelength.

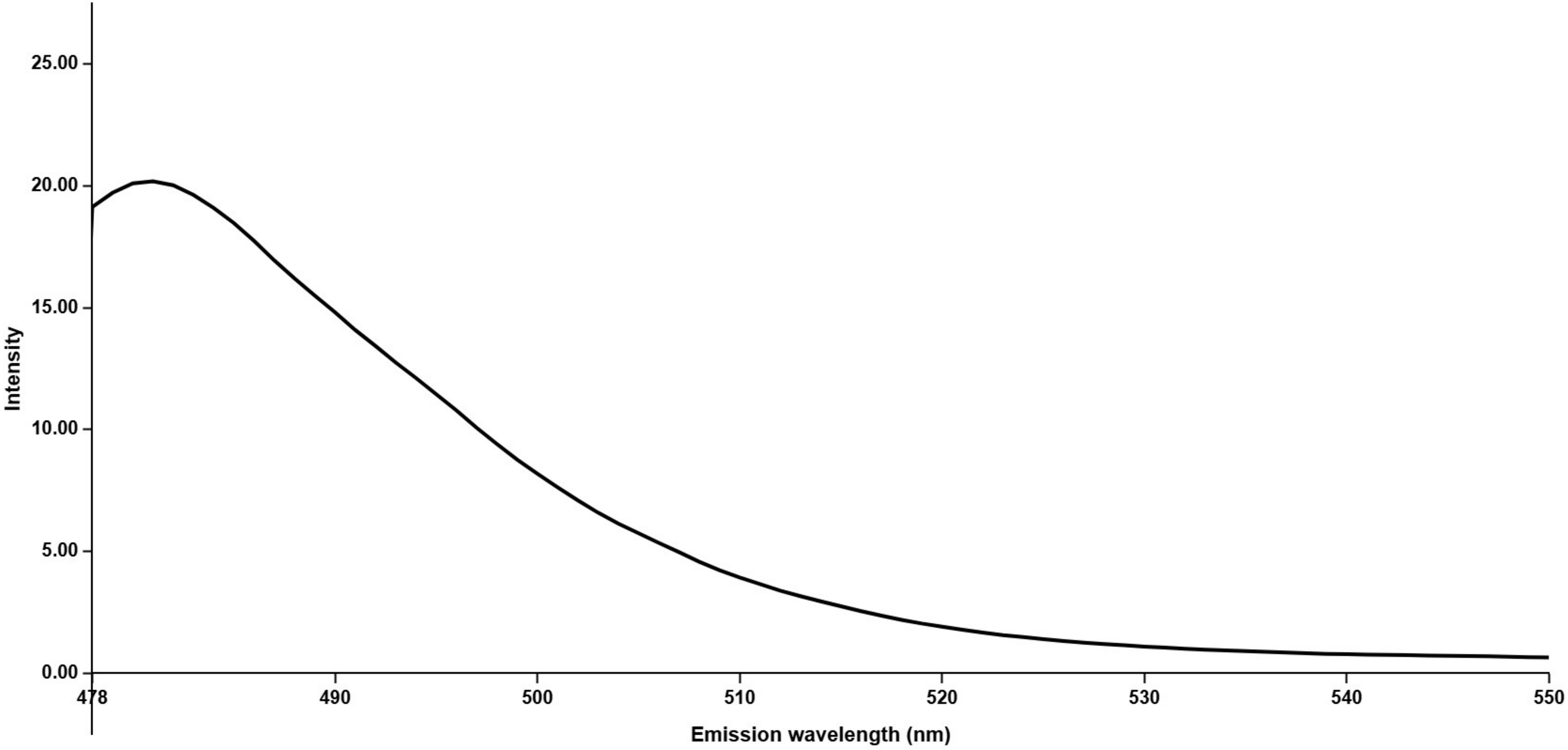

Figure S73. 2D fluorescence emission spectra for compound 3 at an excitation wavelength of λ_{ex} = 468 nm, showing intensity as a function of emission wavelength.

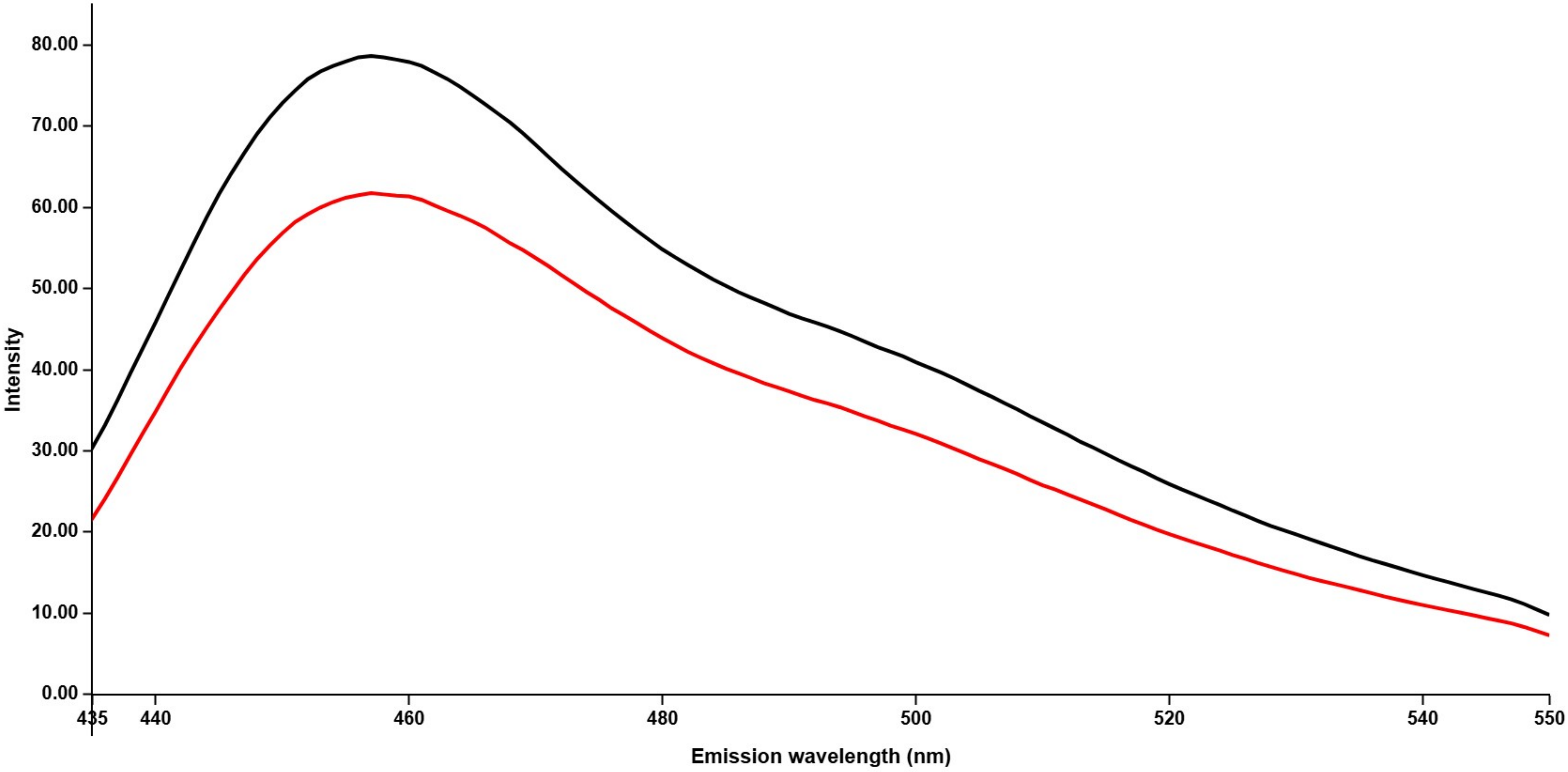

Figure S74. 2D fluorescence emission spectra for compound 4 at two excitation wavelengths: black line at λ_{ex} = 424 nm and red line at λ_{ex}= 366 nm, showing intensity as a function of emission wavelength.

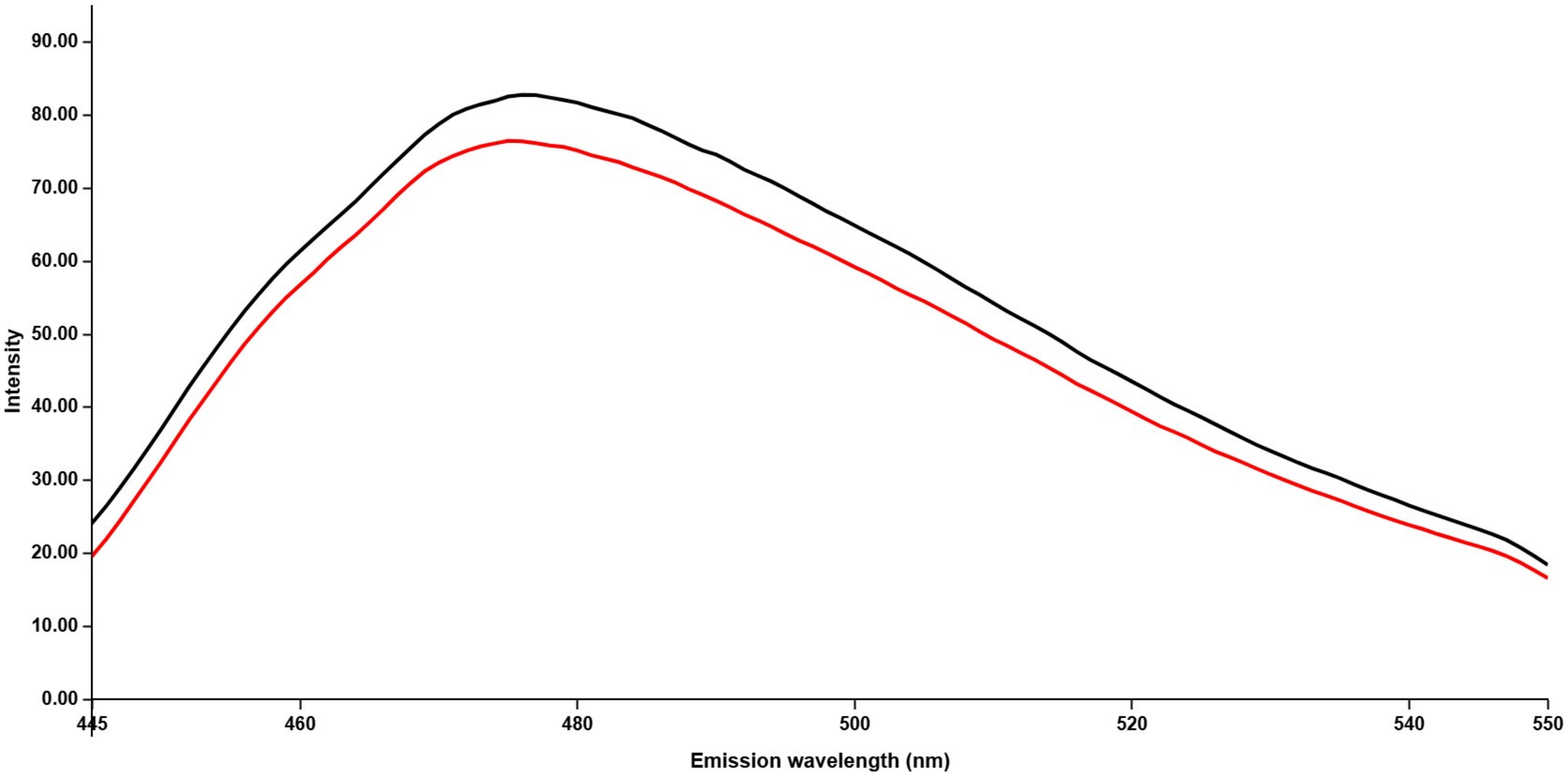

Figure S75. 2D fluorescence emission spectra for compound 5 at two excitation wavelengths: black line at λ_{ex} = 431 nm and red line at λ_{ex}= 359 nm, showing intensity as a function of emission wavelength.

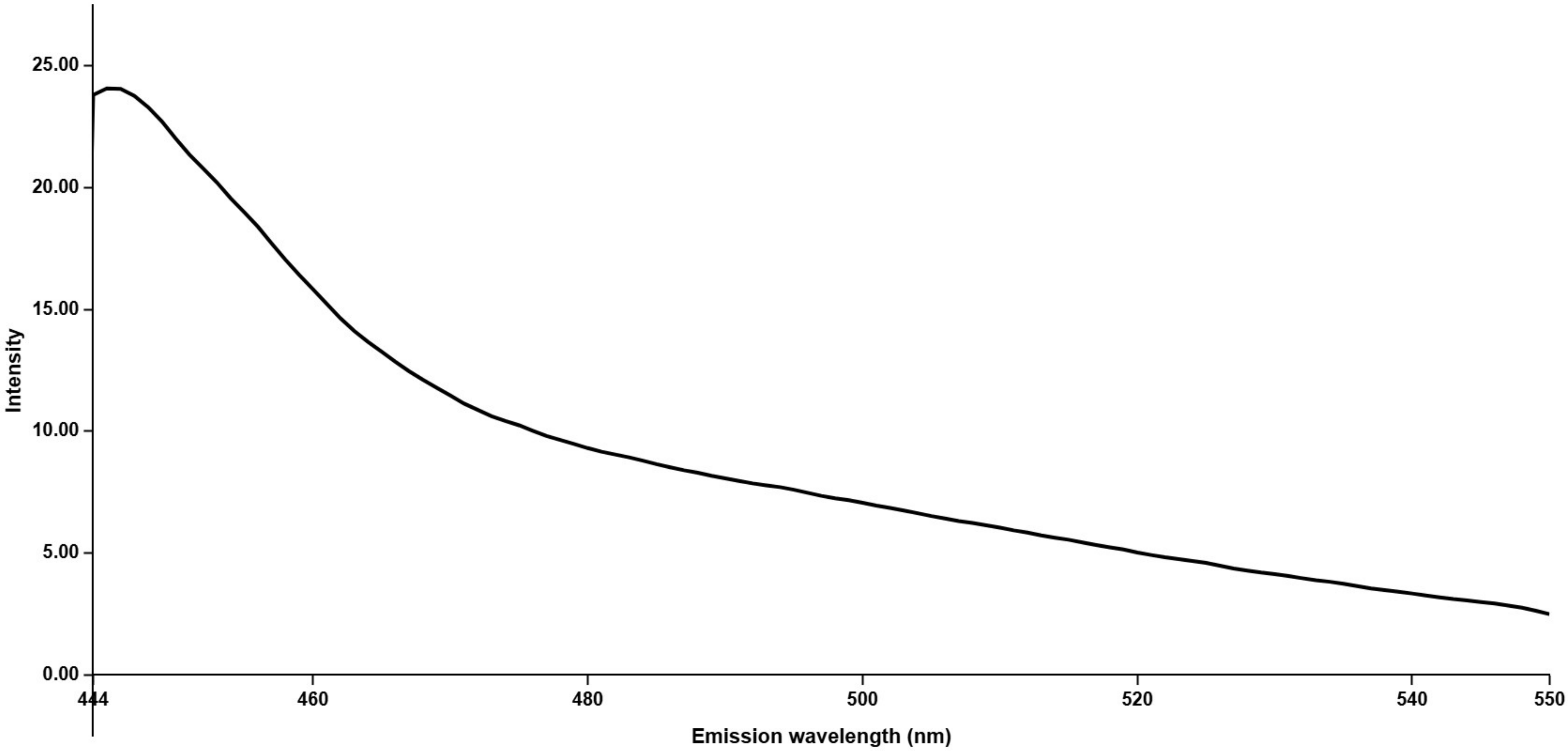

Figure S76. 2D fluorescence emission spectra for compound 6 at an excitation wavelength of λ_{ex} = 434 nm, showing intensity as a function of emission wavelength.

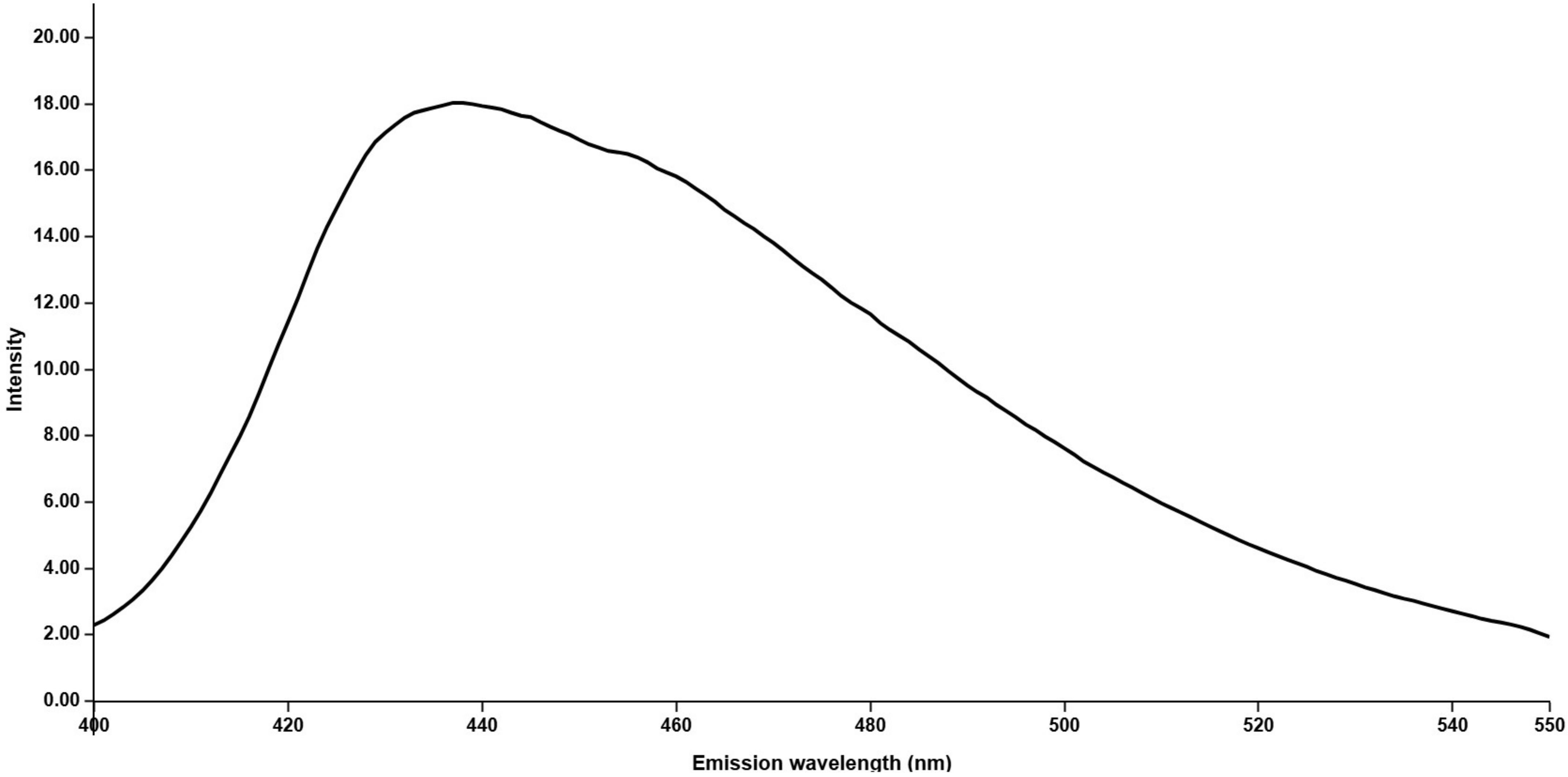

Figure S77. 2D fluorescence emission spectra for compound 6 at an excitation wavelength of λ_{ex} = 364 nm, showing intensity as a function of emission wavelength.

Table S1. Crystal data and structure refinement details for studied compounds.

Compound(CCDC number)	**1** (2350550)	**2** (2350546)	**3** (23505440)	**4** (2350548)	**5** (2350551)	**6** (2350543)	**7** (2350542)	**8** (2350545)	**9** (2350547)	**10** (2350549)
Empirical formula	$C_{15}H_{16}N_2O_2$	$C_{16}H_{19}N_3O$	$C_{14}H_{14}N_2O_2$	$C_{18}H_{21}N_3O_2$	$C_{14}H_{12}Cl_2N_2O$	$C_{15}H_{15}BrN_2O$	$C_{12}H_{11}N_3O_4$	$C_{14}H_{13}N_3O_3$	$C_{16}H_{15}N_3O_3$	$C_{12}H_{11}N_3O_3S$
Formula weight	256.30	269.34	242.27	311.38	295.16	319.20	261.24	271.27	297.31	277.30
Temperature (K)	99.9(5)	100.01(13)	99.9(5)	99.9(6)	99.9(5)	100.02(10)	99.9(5)	99.9(5)	99.9(6)	105(7)
Crystal system	orthorhombic	monoclinic	monoclinic	orthorhombic	monoclinic	orthorhombic	triclinic	monoclinic	monoclinic	monoclinic
Space group	$P2_12_12_1$	$P2_1$	$P2_1/c$	$P2_12_12_1$	$P2_1/c$	$P2_12_12_1$	P-1	$P2_1/c$	$P2_1/c$	$P2_1/n$
a (Å)	5.19160(10)	11.95080(10)	26.7493(5)	5.97860(10)	4.61560(10)	5.30400(10)	6.4229(3)	14.3070(3)	16.2030(2)	9.92380(10)
b (Å)	8.53580(10)	7.29470(10)	5.80660(10)	7.24420(10)	9.49270(10)	8.79880(10)	7.1501(2)	7.29090(10)	13.03460(10)	23.4635(3)
c (Å)	29.4619(5)	16.1641(2)	7.7294(2)	36.2940(5)	30.1240(4)	29.2208(3)	13.0240(4)	13.4037(2)	6.76630(10)	11.4920(2)
α (°)	90	90	90	90	90	90	74.165(2)	90	90	90
β (°)	90	97.8960(10)	95.511(2)	90	93.5470(10)	90	84.153(3)	114.076(2)	94.0550(10)	105.8050(10)
γ (°)	90	90	90	90	90	90	86.204(3)	90	90	90
Volume (Å^3)	1305.59(4)	1395.79(3)	1195.00(4)	1571.90(4)	1317.34(4)	1363.70(3)	571.97(4)	1276.52(4)	1425.46(3)	2574.71(6)
Z	4	4	4	4	4	4	2	4	4	8
ρ_{calc} (g/cm^3)	1.304	1.282	1.347	1.316	1.488	1.555	1.517	1.412	1.385	1.431
μ (mm^{-1})	0.709	0.652	0.744	0.702	4.371	4.050	0.986	0.845	0.808	2.326
F(000)	544.0	576.0	512.0	664.0	608.0	648.0	272.0	568.0	624.0	1152.0
Crystal size (*mm^3*)	0.221 × 0.085 × 0.052	0.251 × 0.033 × 0.018	0.176 × 0.126 × 0.054	0.238 × 0.074 × 0.025	0.36 × 0.09 × 0.05	0.207 × 0.031 × 0.028	0.324 × 0.066 × 0.034	0.287 × 0.195 × 0.102	0.17 × 0.136 × 0.02	0.16 × 0.12 × 0.07
Radiation	Cu Kα (λ = 1.54184)	Cu Kα (λ = 1.54184)	Cu Kα (λ = 1.54184)	Cu Kα (λ = 1.54184)	Cu Kα (λ = 1.54184)	Cu Kα (λ = 1.54184)	Cu Kα (λ = 1.54184)	Cu Kα (λ = 1.54184)	Cu Kα (λ = 1.54184)	Cu Kα (λ = 1.54184)
2θ range for data collect. (°)	10.792 to 158.232	5.52 to 158.88	9.966 to 158.894	9.748 to 159.114	5.878 to 158.858	6.05 to 159.344	7.082 to 159.52	13.24 to 158.876	8.716 to 159.158	7.536 to 158.642
Index ranges	-6 ≤ h ≤ 6, -10 ≤ k ≤ 8, -35 ≤ l ≤ 36	-15 ≤ h ≤ 15, -7 ≤ k ≤ 9, -20 ≤ l ≤ 20	-32 ≤ h ≤ 32, -5 ≤ k ≤ 7, -9 ≤ l ≤ 8	-7 ≤ h ≤ 5, -9 ≤ k ≤ 9, -46 ≤ l ≤ 45	-4 ≤ h ≤ 5, -11 ≤ k ≤ 11, -32 ≤ l ≤ 38	-6 ≤ h ≤ 5, -10 ≤ k ≤ 11, -37 ≤ l ≤ 35	-7 ≤ h ≤ 8, -8 ≤ k ≤ 8, -16 ≤ l ≤ 16	-17 ≤ h ≤ 16, -8 ≤ k ≤ 9, -16 ≤ l ≤ 15	-19 ≤ h ≤ 20, -16 ≤ k ≤ 15, -8 ≤ l ≤ 7	-12 ≤ h ≤ 12, -24 ≤ k ≤ 29, -14 ≤ l ≤ 13
Reflections collected/ independent	13101/2692	27539/4881	12265/2425	29983/3310	13626/2694	13843/2847	2527/2527	12901/2536	27673/3013	26227/5353
R_{int}	0.0318	0.0377	0.0265	0.0644	0.0261	0.0285	-	0.0220	0.0324	0.0231
Data/restraints/parameters	2692/0/177	4881/1/373	2425/0/170	3310/0/212	2694/0/176	2847/0/177	2527/0/177	2536/0/182	3013/0/203	5353/0/351
Goodness-of-fit on F^2	1.047	1.067	1.198	1.194	1.086	1.042	1.115	1.080	1.275	1.063
Final *R* indexes [I>2σ(I)]	R_1 = 0.0286, wR_2 = 0.0734	R_1 = 0.0351, wR_2 = 0.0937	R_1 = 0.0478, wR_2 = 0.1290	R_1 = 0.0598, wR_2 = 0.0936	R_1 = 0.0240, wR_2 = 0.0659	R_1 = 0.0209, wR_2 = 0.0507	R_1 = 0.0377, wR_2 = 0.1186	R_1 = 0.0351, wR_2 = 0.0972	R_1 = 0.0612, wR_2 = 0.1734	R_1 = 0.0307, wR_2 = 0.0870
R indexes (all data)	R_1 = 0.0303, wR_2 = 0.0746	R_1 = 0.0377, wR_2 = 0.0990	R_1 = 0.0511, wR_2 = 0.1303	R_1 = 0.0637, wR_2 = 0.0953	R_1 = 0.0249, wR_2 = 0.0664	R_1 = 0.0218, wR_2 = 0.0510	R_1 = 0.0399, wR_2 = 0.1211	R_1 = 0.0368, wR_2 = 0.0985	R_1 = 0.0649, wR_2 = 0.1747	R_1 = 0.0340, wR_2 = 0.0890
Largest diff. peak and hole (e•Å^{-3})	0.19/-0.18	0.25/-0.22	0.28/-0.26	0.27/-0.22	0.22/-0.21	0.26/-0.27	0.23/-0.32	0.41/-0.40	0.44/-0.27	0.29/-0.28

Table S2. Selected bond lengths (Å) for the studied compounds.

Compound	1	2* (C1-C16)	2*(C21-C36)	3	4	5	6	7	8	9	10*(C1-C12)	10*(C21-C32)
C1—O1	1.423(2)	1.425(3)	1.421(3)	1.427(3)	1.427(3)	1.4267(15)	1.419(3)	1.4284(15)	1.4323(13)	1.429(3)	1.4253(15)	1.4276(16)
C2—O1	1.373(2)	1.381(2)	1.383(3)	1.381(2)	1.373(3)	1.3691(14)	1.371(3)	1.3715(15)	1.3807(13)	1.375(3)	1.3772(14)	1.3774(14)
C5—N1	1.414(2)	1.400(3)	1.412(3)	1.407(3)	1.399(3)	1.4058(15)	1.403(3)	1.3996(16)	1.3997(14)	1.403(3)	1.4049(15)	1.4042(15)
N1—N2	1.343(2)	1.376(2)	1.379(2)	1.359(2)	1.367(3)	1.3571(14)	1.360(3)	1.3346(15)	1.3548(13)	1.352(3)	1.3352(14)	1.3408(14)
C8—N2	1.302(2)	1.287(3)	1.287(3)	1.293(3)	1.287(3)	1.2830(15)	1.296(3)	1.2965(17)	1.2888(14)	1.291(3)	1.2947(15)	1.2948(15)
C8—C9	1.448(2)	1.462(3)	1.453(3)	1.452(3)	1.463(3)	1.4651(16)	1.485(4)	1.4314(17)	1.4579(15)	1.435(4)	1.4355(17)	1.4337(16)

*Z'=2

Table S3. Selected bond angles (°) for the studied compounds.

Compound	1	2* (C1-C16)	2* (C21-C36)	3	4	5	6	7	8	9	10*(C1-C12)	10*(C21-C32)
C1—O1—C2	116.85(13)	116.88(16)	116.87(18)	116.57(17)	117.1(2)	116.89(9)	118.1(2)	116.82(10)	117.10(8)	116.8(2)	117.50(10)	117.63(10)
C3—C2—O1	124.80(15)	124.50(19)	124.8(2)	115.61(19)	115.2(2)	124.92(11)	125.0(3)	115.98(11)	124.53(9)	115.5(2)	124.62(11)	124.70(11)
C7—C2—O1	115.83(14)	115.55(18)	115.43(19)	124.53(19)	125.3(2)	115.75(10)	115.4(2)	124.64(11)	115.42(9)	124.5(2)	115.69(11)	115.44(11)
C4—C5—N1	122.80(15)	122.92(18)	122.1(2)	123.04(19)	121.5(2)	122.39(10)	122.6(2)	122.14(11)	123.12(10)	122.0(2)	121.61(11)	121.60(11)
C6—C5—N1	118.52(14)	118.48(18)	118.66(19)	117.31(19)	119.4(2)	118.47(10)	118.1(2)	118.41(11)	117.50(9)	118.3(2)	118.74(11)	118.98(11)
C5—N1—N2	119.85(14)	119.09(17)	116.91(17)	120.76(18)	118.7(2)	118.78(10)	119.6(2)	120.78(11)	120.68(9)	120.9(2)	119.52(10)	119.89(10)
N1—N2—C8	117.90(14)	114.91(17)	115.72(19)	116.55(18)	117.2(2	117.81(10)	118.3(2)	117.10(11)	115.75(9)	116.3(2)	118.72(10)	117.60(10)
N2—C8—C9	120.48(14)	122.12(19)	122.67(19)	122.0(2)	121.5(2)	119.31(10)	116.1(2)	117.88(11)	121.98(10)	121.0(3)	117.77(11)	119.02(11)

*Z'=2

Table S4. Interplane angles (φ) between phenylene and phenylene/furan/thiophene ring mean planes, and selected torsion angles (χ) in the studied compounds.

Compound	φ (°)	χ [C5-N1-N2-C8] (°)	χ [N1-N2-C8-C9] (°)	χ [N2-C8-C9-C10] (°)	χ [C8-C9-C10-C11] (°)
**1**	2.6	-174.55(14)	-179.40(14)	-	-
**2* (C1-C16)**	15.1	167.7(2)	175.24(19)	-	-
**2* (C21-C36)**	28.5	-155.0(2)	179.4(2)	-	-
**3**	16.5	-162.61(19)	-179.30(18)	-	-
**4**	13.1	-174.8(2)	179.3(2)	-	-
**5**	10.1	-168.37(10)	-176.07(9)	-	-
**6**	4.7	173.5(2)	177.9(2)	-	-
**7**	3.4	177.54(11)	179.94(10)	-	-
**8**	14.4	167.35(10)	177.22(9)	-	-
**9**	7.2	-178.6(2)	179.3(2)	-175.4(3)	-175.8(3)
**10*(C1-C12)**	20.5	-179.93(11)	-178.06(10)	-	-
**10*(C21-C32)**	24.9	-177.28(11)	177.01(11)	-	-

*Z'=2

Table S5. Classical hydrogen bonds in studied compounds [$G_d^a(n)$ – unitary graph set].

D-H•••A	d(D—H) (Å)	d(H•••A) (Å)	d(D•••A) (Å)	<(DHA) (°)	$G_d^a(n)$
			**2**		
N1—H1•••N1$^{(i)}$	0.90(3)	2.77(3)	3.255(3)	115(2)	C(2)
N1—H1•••N21	0.90(3)	3.21(3)	3.712(3)	118(2)	D(2)
N21—H21•••N1	0.92(3)	2.90(2)	3.255(3)	105(2)	D(2)
N21—H21•••N2$^{(ii)}$	0.92(3)	3.29(2)	4.169(3)	163(2)	D(2)
			**3**		
N1—H1•••O2$^{(iii)}$	0.89(3)	2.51(3)	3.069(2)	121.2(18)	C(7)
N1—H1•••O2$^{(iv)}$	0.89(3)	2.53(2)	3.127(2)	125(2)	C(7)
O2—H2•••N2	0.93(3)	1.82(3)	2.637(2)	146(2)	S(6)
			**4**		
N1—H1•••O2$^{(v)}$	0.86(3)	2.51(3)	3.264(3)	148(3)	C(12)
			**7**		
N1—H1•••O4$^{(vi)}$	0.891(17)	2.153(16)	2.9916(14)	156.7(16)	$R_2^2(18)$
			**8**		
N1—H1•••O1$^{(iv)}$	0.865(14)	2.263(14)	3.0100(12)	144.7(13)	C(7)
			**10**		
N1—H1•••O2$^{(vii)}$	0.864(16)	2.561(15)	3.3236(15)	147.7(12)	C(9)
N1—H1•••O3$^{(vii)}$	0.864(16)	2.372(16)	3.1855(16)	157.2(13)	C(9)
N21—H21•••O22$^{(viii)}$	0.877(16)	2.520(15)	3.3074(15)	149.8(12)	C(9)
N21—H21•••O23$^{(viii)}$	0.877(16)	2.354(16)	3.1740(16)	155.8(12)	C(9)

Symmetry transformations used to generate equivalent atoms: (i) -*x*+1, *y*+0.5, -*z*+1; (ii) -*x*+1, y-0.5, -*z*+1; (iii) *x*, *y*+1, *z*; (iv) *x*, -*y*+0.5, *z*+0.5; (v) *x*-0.5, -*y*+1.5, -*z*+1; (vi) -*x*, -*y*+2, -*z*+1; (vii) *x*-0.5, -*y*+0.5, *z*+0.5; (viii) *x*+0.5, -*y*+1.5, *z*-0.5.

Table S6. Stacking interactions in the studied compounds. Each ring, R, is indicated by one membered atom. The d is a distance between centroid of I, Cg(I), and centroid of J, Cg(J), α is a dihedral angle between planes of rings I and J, β is an angle between Cg(I)-Cg(J) vector and normal to plane I, d_p is a perpendicular distance of Cg(J) on the ring I plane.

I•••J	d (Å)	α (°)	β (°)	d_p (Å)
		**7**		
R(C2)•••R(C9)$^{(i)}$	3.4582(8)	3.43(7)	15.0	3.3402(6)
R(C2)•••R(C9)$^{(ii)}$	3.7360(8)	3.43(7)	27.1	3.3268(6)
		**8**		
R(C9)•••R(C9)$^{(i)}$	3.6324(6)	0.03(5)	23.4	3.3334(4)
		**9**		
R(C2)•••R(C11)$^{(i)}$	3.8321(15)	7.22(13)	24.7	3.4820(11)

Symmetry transformations used to generate equivalent atoms: (i) -*x*+1, -*y*+1, -*z*+1; (ii) -*x*+1, -*y*+2, -*z*+1.

Table S7. Absorption data from UV-Vis diffuse reflectance spectra (experimental) and quantum-mechanical calculations (theoretical) for the studied compounds.

Compound	Wavelength of absorption maxima (nm)		Oscillator strength
	Experimental	Theoretical	
**1**	395	224	0.212
		272	0.324
		300	0.106
		353	1.014
	450	385	0.013
**2**	243	226	0.189
	286	286	0.496
	328	356	0.670
	470	373	0.005
**3**	236	245	0.544
	299	302	0.080
	356	351	0.775
	415	397	0.010
		453	0.003
**4**	251	235	0.170
	396	282	0.728
		354	1.508
	477	404	0.014
		408	0.009
**5**	253	222	0.193
		267	0.553
	341	306	0.062
	400	359	1.206
	508	395	0.007
**6**	242	220	0.156
		231	0.155
	294	264	0.449
		293	0.078
	337	343	0.087
		357	1.039
	378	372	0.079
	480	409	0.019
**7**	228	223	0.054
	290	318	0.421
	331	354	0.237
	410	437	1.000
	598	539	0.191
		575	0.096
**8**	251	240	0.191
	365	297	0.444
		312	0.312
		339	0.205
	523	452	1.100
		526	0.552
**9**	275	222	0.154
		260	0.202
	334	322	1.260
	391	352	0.343
		460	1.539
	514	518	0.361
**10**	241	243	0.144
	290	339	0.214
	446	469	0.471
		511	0.462
	678	574	0.163
		664	0.005
